# Titmice are a better indicator of bird density in Northern European than in Western European forests

**DOI:** 10.1002/ece3.8479

**Published:** 2022-02-12

**Authors:** Mira H. Kajanus, Jukka T. Forsman, Maximilian G. R. Vollstädt, Vincent Devictor, Merja Elo, Aleksi Lehikoinen, Mikko Mönkkönen, James T. Thorson, Sami M. Kivelä

**Affiliations:** ^1^ Department of Ecology and Genetics University of Oulu Oulu Finland; ^2^ Natural Resources Institute Finland Oulu Finland; ^3^ Center for Macroecology, Evolution and Climate GLOBE Institute University of Copenhagen Copenhagen Denmark; ^4^ ISEM, Univ Montpellier, CNRS, EPHE, IRD Montpellier France; ^5^ Department of Biological and Environmental Science University of Jyväskylä Jyväskylä Finland; ^6^ Finnish Museum of Natural History Helsinki Finland; ^7^ National Marine Fisheries Service, NOAA Seattle Washington USA

**Keywords:** citizen science, long‐term monitoring, macroecology, spatial Gompertz model, surrogate, VAST

## Abstract

Population sizes of many birds are declining alarmingly and methods for estimating fluctuations in species’ abundances at a large spatial scale are needed. The possibility to derive indicators from the tendency of specific species to co‐occur with others has been overlooked. Here, we tested whether the abundance of resident titmice can act as a general ecological indicator of forest bird density in European forests. Titmice species are easily identifiable and have a wide distribution, which makes them potentially useful ecological indicators. Migratory birds often use information on the density of resident birds, such as titmice, as a cue for habitat selection. Thus, the density of residents may potentially affect community dynamics. We examined spatio‐temporal variation in titmouse abundance and total bird abundance, each measured as biomass, by using long‐term citizen science data on breeding forest birds in Finland and France. We analyzed the variation in observed forest bird density (excluding titmice) in relation to titmouse abundance. In Finland, forest bird density linearly increased with titmouse abundance. In France, forest bird density nonlinearly increased with titmouse abundance, the association weakening toward high titmouse abundance. We then analyzed whether the abundance (measured as biomass) of random species sets could predict forest bird density better than titmouse abundance. Random species sets outperformed titmice as an indicator of forest bird density only in 4.4% and 24.2% of the random draws, in Finland and France, respectively. Overall, the results suggest that titmice could act as an indicator of bird density in Northern European forest bird communities, encouraging the use of titmice observations by even less‐experienced observers in citizen science monitoring of general forest bird density.

## Graphical abstract

Methods for estimating fluctuations in forest bird species’ abundances at a large spatial scale are needed, and conspicuous and widely distributed titmice are potentially good ecological indicators. We found that titmouse abundance and other forest bird density had a linear positive association in Finland. These results, together with a random draw analysis, suggested that titmice could be used as indicators of Northern forest bird communities allowing the use of less‐experienced observers in bird monitoring.

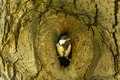

## 1 INTRODUCTION

Species abundances are dynamic and sensitive to environmental change (Hughes, 2000; Lemoine et al., 2007) and abundances of many animals are declining globally at increasing rates as a response to global change (Brondizio et al., 2019). Birds are among the best studied taxa in this respect, and alarming evidence is accumulating about recent declines in population sizes for many species (Bowler et al., 2019; Rosenberg et al., 2019). In the face of this biological crisis, we need methods for estimating population abundances at extensive spatial scales more than ever. To get spatially extensive abundance data, citizen science approach is a well‐established method (Devictor et al., 2010; Jiguet et al., 2012). However, for the citizen science approach to work reliably, easy methods, that nonexperts can consistently use are required (McKinley et al., 2017; Silvertown, 2009). Hence, using the abundance of easily identifiable and conspicuous species as an indicator when estimating abundance of a target species group may facilitate the use of citizen science approach in abundance estimation in general.

Using indicator species has yielded promising results in community ecology (Caro & O'Doherty, 1999; Fleishman et al., 2005; Menon & Shahabuddin, 2021; Sattler et al., 2013). The most effective ecological indicators are usually species that are closely related to the target species (Fleishman et al., 2005; Sattler et al., 2013). For example, Lindenmayer et al. (2014) propose that a specific marsupial species could be used as an indicator for the abundance of other species of the same taxonomic group. However, simultaneously using multiple species is proposed to be a more effective ecological indicator for species diversity than merely one indicator species (Fleishman et al., 2005; Grenyer et al., 2006; Morelli et al., 2014; Padoa‐Schioppa et al., 2006; Sattler et al., 2013) and the same probably holds for abundance too. This is because a group of indicator species could capture a wider variation of ecological traits in target species than a single indicator species (Gregory et al., 2005; Morelli, 2015).

The use of indicator species to estimate target species occurrence or abundance can be based on similar responses to abiotic factors (Caro & O'Doherty, 1999; Sætersdal et al., 2003) or biotic associations between the indicator and target species groups (Møller et al., 2017; Sergio et al., 2006). Even though negative interspecific interactions, such as competition, can negatively affect species co‐occurrence and abundance (Forsman et al., 2008; Goldberg & Barton, 1992), shared habitat preference and positive interspecific interactions may promote co‐occurrence of species and create aggregations of individuals (Basile et al., 2021; Forsman et al., 2009). Positive interactions among species include, for example, facilitation (Gross, 2008), active attraction to heterospecifics (Forsman et al., 2009; Mönkkönen et al., 1990; Thomson et al., 2003), and social information use (i.e., the use of inadvertent cues produced by other species when making decisions on resource use; Gross, 2008; Seppänen et al., 2007; Vazquez & Simberloff, 2003), all of which can lead to positive associations between species abundances. Through positive interactions, such as social information use across species, species’ resource use and interactions with other species may be altered, thereby changing community dynamics (Forsman et al., 2002, 2009; Mönkkönen et al., 2017; Seppänen et al., 2007; Wisz et al., 2013).

In this study, we examine the effectiveness of using a group of species to estimate the density of target species at extensive geographical and temporal scales. Our study system is the European forest bird community, where we study whether the conspicuous and easy‐to‐identify species of titmice (family Paridae) could indicate density of breeding forest birds. In Asia, species richness of titmice has been found to be a poor indicator of overall bird species richness (Møller et al., 2017). However, Møller et al. (2017) observed a maximum of two titmouse species, while titmice are more diverse in Europe (del Hoyo et al., 2007). Additionally, other bird species groups, such as woodpeckers (family Picidae), are suggested to be efficient ecological indicators for bird species diversity (Menon & Shahabuddin, 2021; Mikusiński et al., 2001). Even with no covariation between titmouse species richness and overall species richness, abundances can still covary. Focusing on abundances offers a finer grain indicator than co‐occurrences or species diversity. Hence, titmice may have more potential for indicating bird abundance than species richness.

Titmice inhabit broadleaf, conifer and mixed forests, and semi‐forested habitats (Eck & Martens, 2006; del Hoyo et al., 2007; Suhonen et al., 1994), and have a wide distribution globally, occurring on all continents except South America, Australia, and Antarctica (del Hoyo et al., 2007; Gill et al., 2005). European titmouse species include both generalist (e.g., great tit [*Parus major*]) and specialist (e.g., coal tit [*Periparus ater*]) species (del Hoyo et al., 2007), suggesting that their ecology overlaps with many other forest bird species and that titmice could serve as an indicator group for bird density in forest habitats in general. Additionally, titmice are conspicuous due to their highly active foraging, social, and vocal behavior (del Hoyo et al., 2007), making them easy to observe. As a group, titmice are often easily recognized by the general public due to their highly similar appearance across species (del Hoyo et al., 2007) and their common visits to garden bird feeders. Additionally, identifying individuals to species level is mainly easy due to the variation of plumage color (Eck & Martens, 2006; del Hoyo et al., 2007), body size (Alatalo & Moreno, 1987; Eck & Martens, 2006; del Hoyo et al., 2007), and crestedness among species (Eck & Martens, 2006; del Hoyo et al., 2007). Overall, the ecology of titmice makes them potentially a more efficient indicator group for forest bird abundance than, for example, woodpeckers or cuckoos, which are less abundant and diverse and consist of mainly specialist species (Menon & Shahabuddin, 2021; Mikusiński et al., 2001; Møller et al., 2017).

As titmouse species are mainly residents (del Hoyo et al., 2007), they experience the environmental changes at the breeding grounds throughout the year, including nonbreeding season. Therefore, they can be more sensitive to changes in habitat quality during a certain period of their life cycle. Resident species also have the advantage of acquiring a lot of information on the environment and establishing their breeding grounds prior to the arrival of migratory birds. Forsman et al. (2009) found that migratory birds use the density of resident titmice with similar ecological niches (i.e., potential competitors) as a cue for habitat selection, which results in a positive association between titmice and migratory birds. Owing to this, titmice may be good indicators of habitat quality for migratory birds, and it may pay off for migratory species to use titmouse density as a cue for habitat selection (Forsman et al., 2009; Mönkkönen et al., 1990), despite the costs of interspecific competition (Forsman et al., 2008; Gustafsson, 1987; Mönkkönen et al., 2004; Sasvári et al., 1987). However, there may be a threshold density of titmice, above which negative effects of interspecific competition between individuals may exceed the benefits of social information use (Forsman et al., 2008; Mönkkönen et al., 2004; Seppänen et al., 2007). Thus, the avoidance of competition may lead to negative associations between titmice and other forest birds at least at high densities.

Here, we examine the association between titmouse abundance (given as biomass) and the density of other birds in forest habitats at a macroecological scale, while controlling for environmental factors (temperature and precipitation). We analyze long‐term citizen science breeding bird survey data from Northern and Western Europe by using a dynamic species distribution model (Thorson, 2019; Thorson & Barnett, 2017). We hypothesize that the abundance of resident titmice acts as a general indicator of bird density in European forests, if similar habitat preferences or social information use, as described by Forsman et al. (2009) generally takes place in forest bird communities. To assess the performance of titmice as indicators of forest bird density, we compare the titmouse group against randomly drawn species groups from the same community (see Andelman & Fagan, 2000; Cabeza et al., 2008; Tognelli, 2005). A suitable ecological indicator should perform better than randomly drawn groups of species in representing the density of forest birds.

## 2 METHODS

### 2.1 Bird surveys

We used breeding forest bird surveys from citizen science programs in Finland (The Finnish Museum of Natural History, LUOMUS) and France (the French Breeding Bird Survey, FBBS). These citizen science data sets (sensu Jiguet et al., 2012) consist of point counts that were carried out similarly each year during the surveys and were performed by experienced volunteer ornithologists with excellent species identification skills. Despite some minor differences (e.g., different number of sampling points per sampling route/plot [see below], two annual surveys in France due to longer breeding season) in survey design between Finland and France, the general methodological similarity makes the two data sets comparable across space and time. Data from 2001 to 2013 were used from both countries. At each point count in both countries, all visually and acoustically observed birds were recorded during a five‐minute observation period independently of the distance from the observer. Most of the observations are based on sounds and binoculars are used to identify distant individuals. Unlimited observation distance (Blondel et al., 1981) ensured that no observations were excluded during sampling due to unreliable distance estimation.

In Finland, the survey area consisted of routes, established by the observers themselves, where the observer performed the point counts on 20 points located a minimum of 250 m apart (Koskimies & Väisänen, 1991; Laaksonen & Lehikoinen, 2013). Each observation is transformed into pairs including observations of (i) singing or displaying, (ii) other calls, (iii) sightings (male, female, pair, brood, or nest), (iv) flying bird, and (v) flying flock. Flocks are transformed into pairs, normally by dividing by two (male and female) plus the mean species‐specific brood size in case of brood flocks. The census unit is a pair of birds, not an individual; thus, a male and a female seen separately or together, or a parent with offspring, is transformed into one pair (Koskimies & Väisänen, 1991). Thus, the observed numbers of individuals were multiplied by two to get the total number of individuals. In France, 2 km × 2 km plots were randomly distributed across the landscape in the beginning of the survey (Jiguet et al., 2012). Within each plot, there were 10 random points a minimum of 300 m apart where the point counts were performed by counting each observed individual as such, and not as a breeding pair (Jiguet et al., 2012). For detailed descriptions of the sampling designs, see Koskimies and Väisänen (1991) and Jiguet et al. (2012), for Finland and France, respectively.

In both countries, all sampling route, plot, and point locations remained constant during the study period. However, not all routes and plots included in the data were sampled for all years of the study period. In Finland, 76 routes, including 939 unique points (Appendix 1: Figure A1), were sampled during breeding time in spring (May–June). The data yielded a total of 63,156 observed forest birds (breeding pairs) and 75 species (see Supporting Information 1 for a list of species: Table S1.1), including six titmouse species. The French data were collected over two sampling periods during each breeding season (April–June) to warrant observations for both early and late breeding species (Jiguet et al., 2012). 1169 plots were sampled, including 4342 unique points (Appendix 1: Figure A2), yielding to a total of 349,886 observed forest birds and 63 species (see Supporting Information 1 for a list of species: Table S1.1), including six titmouse species. The data from the two sampling periods in France were summed to comprise one annual data set for each point.

Both surveys are designed for monitoring birds breeding in terrestrial habitats. We only considered data from forest habitats in this study (see Supporting Information 1 for a list of forest classifications: Table S1.2). We used our expertise for restricting the bird data to only include species breeding and/or foraging in forests. Different sampling designs compared to those used in the Finnish and French breeding bird surveys are generally suggested for counting raptors, grouse, waders, and waterfowl (Andersen, 2007; Conway & Nadeau, 2010; Cummins et al., 2015; Hansen et al., 2015; Lor & Malecki, 2002; Pakkala et al., 1983). Thus, raptors, grouse, waders, and waterfowl were excluded from the analyses. The data of each point count in each sampled year were subdivided into two groups: (1) titmouse observations (referred to as titmouse group hereafter) and (2) all other (nontitmice) forest bird species (referred to as forest birds hereafter).

### 2.2 Environmental data

Both titmouse and forest bird density may be positively correlated with environmental productivity, for example, because of resource limitations (Forsman & Mönkkönen, 2003; Hawkins et al., 2003; Mönkkönen et al., 2006; Pautasso et al., 2011). This potentially confounding effect must be considered in statistical analyses, which is why we summarized variation in mean monthly precipitation (mm) and temperature (°C) to a principal component (PC), derived from a principal component analysis (PCA), that serves as a proxy for environmental productivity. We used climate data from the CHELSA–database (Karger et al., 2017) with one‐kilometer resolution. Data were downloaded and handled with ArcGIS Desktop 10.6. software (ESRI, 2019). The derived environmental PC was then used to predict forest bird density at each site (i.e., route or plot), such that we account for this relationship while testing for positive correlation between titmouse abundance and forest bird density.

Mean monthly precipitation and mean monthly temperature were calculated for the geographical centroid of each route and plot, in Finland and France, respectively. As the sampling designs differ between the two countries, we specified a unique radius for each country for extracting the climate data to ensure that the data represent the entire potential area (i.e., all unique points along the route/plot) from which the bird data were collected. In Finland, we used a five‐kilometer radius around the centroid of the route (the extreme points in the Finnish routes may be >10 km apart). In France, a one‐kilometer radius around the centroid of each 2 km × 2 km plot was used to obtain the climate data. For some routes and plots, precipitation (mm) or temperature (°C) values varied a lot due to steep altitudinal gradients within the considered area. To make sure that the climate data accurately represented the conditions at the actual sampling points, we excluded all cases where the monthly ranges of precipitation exceeded 50 mm or temperature variation exceeded 11°C among the grid cells included within the used radius. We calculated the sum of the mean monthly precipitation (mm) and the mean of the mean monthly temperature (°C) for each sampling route or plot for each year and used the year‐specific values in the analysis. We ran a PCA for the annual precipitation and temperature data to derive an index (PC score) to represent the environmental conditions at each site and year. Environmental productivity increases with higher precipitation and temperature (Boisvenue & Running, 2006; Field et al., 1998). Therefore, we used the principal component (i.e., PC1 or PC2) that was positively correlated with both precipitation and temperature as the proxy for productivity (see Supporting Information 1 for details: Figure S1.1) in each country.

Differences in forest structures potentially affect abundances of bird species (Fraixedas et al., 2015; Lehikoinen et al., 2017). Thus, to avoid bias from the heterogeneity of different habitat types, we first subset the habitat types into main habitat classes according to their structural differences. Second, we estimated habitat‐specific Shannon–Wiener diversity indices (*H*′; Shannon & Weaver, 1949). Third, we combined habitats having similar Shannon entropies into the main habitat classes. Consequently, we defined four habitat classes in Finland and five in France (Supporting Information 2) and then repeated the analysis explained below separately for each of the habitat class‐specific subsets of the data (see Supporting Information 2 for results).

### 2.3 Spatial Gompertz model for analyzing forest bird density

Recently, species distribution models (SDMs) have been used to identify indicators for biodiversity (Morelli et al., 2014; Valerio et al., 2016; Vallecillo et al., 2016). The strength of using SDMs in making conservation decisions lies in the possibility to combine spatial environmental and biotic data (Guisan et al., 2013). We applied a dynamic SDM, a spatio‐temporal model that captures localized density dependence in the interannual dynamics for a response variable, while also incorporating covariates to explain residual variation in density. Specifically, we tailored the dynamic SDM so that temporal abundance changes were described by a Gompertz model, where per‐capita productivity is a linear function of log‐transformed total bird density. The Gompertz model is appropriate for modeling temporal abundance changes as it can be used in describing population dynamics of natural populations (Saitoh et al., 1997) and it is widely used in time‐series analysis (Dennis & Taper, 1994). Specifically, we used a “spatial Gompertz” model that includes spatial correlations among localized densities for nearby sites (Thorson, Skaug, et al., 2015). Models incorporating dynamic spatial structure and process errors, such as the spatial Gompertz model, enhance the identification of species codistributions (Kareiva, 1990; Nadeem et al., 2016; Thorson, Skaug, et al., 2015). Thus, the use of dynamic SDMs enables to estimate to which degree a predictor variable (titmouse abundance) explains variation in the response variable (forest bird density) while accounting for spatio‐temporal variation and environmental factors, which facilitates estimation of the effectiveness of an ecological indicator.

We used observed titmouse abundance, given as biomass, as a predictor variable for forest bird density (excluding titmice) in bird communities in Finland and France. A simple spatial Gompertz model was built by using the Vector‐Autoregressive Spatio‐Temporal model (VAST), release number 2.0.1 (available as an R package *VAST*; Thorson & Barnett, 2017, Thorson, 2019). The spatial Gompertz model can be defined in VAST with particular settings (i.e., univariate model with constant intercepts across years and autoregressive process for spatio‐temporal variation; see Thorson, Skaug, et al., 2015) that were used here. The observation data were converted to biomass (g) by multiplying the species‐specific numbers of individuals observed by species‐specific body mass estimates from del Hoyo et al. (2014). Abundance of forest birds, given as biomass, was set as a response variable in the univariate spatio‐temporal model. VAST uses forest bird abundance, together with the sampling area, to model forest bird population density (biomass per unit area), *d*, at location *s* and year *t*, *d*(*s*, *t*) (see Table 1 for definition of all symbols). We set the statistical sampling area to be (circular) 0.031 km^2^ here because we assume that the vast majority of observations have been made within this distance from the observer. Although the radius for the sampling area is set subjectively, it does not affect the conclusions. The radius only affects the scale of the density estimates (i.e., biomass per unit area), and not the estimated relative densities among sampling locations, only relative differences among sampling locations contributing to inferences.

**TABLE 1:** Symbols used for indices, data, fixed effects, random effects, and derived quantities

Symbol	Description	Dimensions
Index		
*i*	Sample	–
*s*	Spatial location (“knot”)	–
*t*	Time interval (year)	–
Data		
*b*	Data for observed forest bird abundance (i.e., biomass; g)	*n_i_*
*a*	Area sampled	1
*tit*	Covariate data for observed titmouse abundance (i.e., biomass; g)	$n_{i}\times n_{t}$
PC	Covariate data for environmental principal component (PC1 or PC2)	$n_{i}\times n_{t}$
control	Covariate data for observed control group species abundance (i.e., biomass; g)	$n_{i}\times n_{t}$
*x*	Number of locations (“knots”) in the spatial mesh used in spatial interpolation	*n*
Fixed effects		
*β*	Intercept for expected forest bird density	*n_i_*
$\sigma_{m}^{2}$	Variance in expected forest bird abundance	*n_i_*
*γ*_1_	Estimated effect of titmouse abundance covariate	1
*γ*_2_	Estimated effect of [titmouse abundance]^2^ covariate	1
*γ*_3_	Estimated effect of environmental principal component (PC1 or PC2) covariate	1
*γ*_4_	Estimated effect of a control group (from a random draw) abundance covariate	1
*ρ_ε_*	Temporal autoregressive correlation in spatio‐temporal variation of forest bird density	1
Random effects		
*ω*	Spatial variation in expected forest bird density	*n_s_*
*ε*	Spatio‐temporal variation in expected forest bird density	$n_{s}\times n_{t}$
$\sigma_{\omega }^{2}$	Variance parameter for spatial variation of expected forest bird density	1
$\sigma_{\varepsilon }^{2}$	Variance parameter for spatio‐temporal variation of expected forest bird density	1
Derived quantities		
*d*	Expected forest bird density (g per km^2^)	$n_{s}\times n_{t}$
***R***	Matrix of spatial correlations in expected forest bird density	$n_{s}\times n_{s}$

Titmouse abundance was used as a covariate in the analysis. In order to consider a possible nonlinear effect of titmice on forest bird density, we also included the square of titmouse abundance as a covariate. Hence, observed titmouse abundance, its square, and the environmental PC (i.e., PC1 or PC2; see Section 2.2) were used as dynamic covariates that vary among sites and years in the analysis. All covariates were standardized prior to analysis (i.e., subtracted by mean and divided by standard deviation). The intercept for expected density of forest birds (*β*) was estimated as a fixed effect independently for each year. Spatio‐temporal variation in expected forest bird density (*ε*) was estimated as a random effect following a Matérn correlation function across space and a first‐order autoregressive process (1‐year lag) across time (see Thorson & Barnett, 2017 for details).

VAST divides the spatial domain into a user specified number (*x*) of spatial knots *s* (that have a specific location) and then predicts density of forest birds *d*(*s*, *t*) for each location *s* and year *t*. The predicted density estimate and covariate values for the observation point of sample *i* are assumed to be equal to the predicted density and covariate values at the nearest location *s_i_* (Thorson, 2019). Thus, covariates are considered at the same spatial scale as the density (Thorson, 2019). For Finland, we defined the number of knots as equal to the number of sampled points (*x* = 939). For France, we used *x* = 1000 in the analysis because tests indicated that the results of the analysis were independent of the number of spatial knots, provided that *x* ≥ 1000 (Supporting Information 1: Table S1.3). Using *x* equal to the number of sampled points (*n* = 4342) was not reasonable for France, because computation time considerably increases with increasing *x*.

We used a generalized linear mixed model for modeling localized densities. Lognormal and gamma distributions for abundance were tested for data from both countries. Gamma distribution models fitted better to the data than models using lognormal distribution (ΔAIC Finland = 115.66, ΔAIC France = 1756.7, in favor of the gamma distribution model) and were therefore used in all subsequent analyses. Hence, the estimation model for the expected abundance (i.e., biomass) of forest birds was:

(1) $$\text{Pr(}b_{i}=B)=\text{gamma}\left\{\left.B\right|a\times d(s_{i},t_{i}),\sigma_{m}^{2}\right\}$$

where *b_i_* is the observed forest bird abundance in sample *i*. gamma(*B*|*d*, *σ* ^2^) describes the gamma probability density function for value *B*, with the mean of *d* and variance of $\sigma_{m}^{2}$, *d*(*s* *_i_, t* *_i_*) being the expected density (g/km^2^) of forest birds at location *s* and year *t*, and *a* is the constant sampling area.

Density of forest birds *d*(*s* *_i_, t*_*i*_) was modeled with a log‐linked linear predictor as:

(2) $$\log\left[d\left(s_{i},t_{i}\right)\right]=\beta \left(t_{i}\right)+\omega \left(s_{i}\right)+\varepsilon \left(s_{i},t_{i}\right)+\gamma_{1}tit\left(s_{i},t_{i}\right)+\gamma_{2}tit^{2}\left(s_{i},t_{i}\right)+\gamma_{3}\text{PC(}s_{i},t_{i})$$

where *β*(*t_i_*) is the intercept for expected density of forest birds at year *t*, *ω*(*s_i_*) is the spatial variation for expected forest bird density across locations *s* (*s* = 1, …, *x*), *ε*(*s_i_* *, t_i_*) is the spatio‐temporal variation for expected forest bird density across locations *s* (*s* = 1, …, *x*) and years *t* (*t* = 2001, …, 2013), and PC(*s_i_*, *t_i_*) is the score of the environmental PC in location *s* in year *t*. The annual intercept *β*(*t_i_*) is specified with each year as a fixed effect. *γ*_1_, *γ*_2_, and *γ*_3_ describe the estimated effects of the three covariates, titmouse abundance (*γ*_1_), its square (*γ*_2_) and environmental PC (PC1 or PC2; *γ*_3_), respectively, on expected density of forest birds. *tit*(*s_i_*, *t_i_*), *tit* ^2^(*s_i_*, *t_i_*), and PC(*s_i_*, *t_i_*) are observed titmouse abundance, square of observed titmouse abundance, and environmental PC score, respectively, that explain variation in forest bird density at a spatial location *s_i_* and in year *t*.

Spatial and spatio‐temporal variation in expected forest bird density *d*(*s_i_*, *t_i_*) were modeled by Gaussian random fields (GRF). The value of the random field at a given observation point of sample *i* was assumed to be equal to the value at the nearest location *s_i_*. We defined the Gaussian process for the spatial variation as:

(3) $$\omega \left(s\right)∼\text{MVN(}0,\sigma_{\omega }^{2}R)$$

where *ω*(*s*) is the spatial variation (GRF) in expected forest bird density at location *s*. MVN describes a multivariate normal probability density function with the mean of zero and an estimated variance parameter $\sigma_{\omega }^{2}$ for spatial variation *ω*(*s*). ***R*** is a spatial correlation matrix between expected forest bird density *d* among locations *s* and assumed stationary, representing the impact of estimated spatial variation *ω*(*s*) on forest bird density. We assumed that spatial autocorrelation is higher for nearby locations than for distant locations. Therefore, spatial autocorrelation was specified using the stochastic partial differentiation equations (SPDE; Lindgren et al., 2011) approximation to a Matérn function (Lindgren et al., 2011), producing a decaying spatial autocorrelation with increasing distance between locations.

Similarly, we used GRF to specify the spatio‐temporal variation:

(4) $$\varepsilon \left(s,t\right)∼\left\{\begin{array}{cc} \text{MVN(}0,R) & \text{if}\;t=t_{1}\\ \text{MVN(}\rho_{\varepsilon }\varepsilon \left(s,t‐1\right),\sigma_{\varepsilon }^{2}R) & \text{if}\;t>t_{1} \end{array}\right.$$

where *ε*(*s*, *t*) is the spatio‐temporal variation in expected forest bird density at location *s* and in year *t*. ***R*** represents the stationary spatial correlation matrix between expected forest bird density *d* among locations *s* and was defined by the Matérn function. *ρ_ε_* is the temporal autocorrelation of spatio‐temporal covariation in expected forest bird density. $\sigma_{\varepsilon }^{2}$ is an estimated variance parameter of *ε*(*s, t*).

For the forest bird density, we estimate the annual intercepts *β*(*t*), the variance in expected forest bird abundance $\sigma_{m}^{2}$, the effect of the three density covariates *γ*_1_, *γ*_2_, *γ*_3_, and the two estimated parameters of the Matérn function governing geometric anisotropy and decorrelation distance as fixed effects. The smoothness parameter for the Matèrn function was fixed to one (*v* = 1). Spatial *ω*(*s*) and spatio‐temporal *ε*(*s, t*) variation and their variance parameters $\sigma_{\omega }^{2}$ and $\sigma_{\varepsilon }^{2}$ were treated as random effects. We used SPDEs to approximate the Gaussian random fields as implemented in the software package R‐INLA (Lindgren, 2012; see Thorson, Skaug, et al., 2015 for details). Parameters were estimated by maximizing the marginal likelihood of fixed effects, given the observed data (Thorson & Barnett, 2017) by using Template Model Builder (TMB; Kristensen et al., 2016) in R version 3.6.0 (R Core Team, 2019). The marginal likelihood and its gradient for fixed effects were calculated using the Laplace approximation (Skaug & Fournier, 2006). The maximum likelihood estimate (MLE) of fixed effects was estimated using a nonlinear optimizer within R statistical environment (R Core Team, 2019). We then estimated the values for random effects that maximize the joint log‐likelihood, given the MLE of fixed effects, using empirical Bayes method in TMB. TMB also estimates standard errors for all fixed and random effects using a generalization of the delta‐method (Kass & Steffey, 1989). More detailed description of the computation is available in Thorson, Skaug, et al. (2015) and the R code for the analysis is provided in the Supporting Information (R code 1). We inferred all the parameters whose 95% confidence intervals did not encompass zero to be statistically significant.

We tested whether there was a need to include the quadratic term of titmouse abundance among the covariates by comparing models with and without the quadratic titmouse effect with Akaike information criterion (AIC) and by checking the statistical significance of the quadratic term. We chose the quadratic model for inferences if that model had a lower AIC value than the model lacking the quadratic effect and the estimated quadratic effect was statistically significant. Otherwise, we based our inferences on the model including only a linear effect of titmouse abundance on forest bird density. The final model fit was assessed by visually inspecting residual plots, produced by VAST and R package “DHARMa” (Hartig, 2020), and assessing the match between predicted and observed densities of forest birds. The diagnostic plots indicated that the models fitted the data well for both countries (Supporting Information 1: Figure S1.2–S1.5). We derived the 95% confidence intervals for the log‐predicted density of forest birds from intercept (*β*) and titmouse covariate parameter (*γ*_1_, *γ*_2_) values that were sampled from a multivariate normal distribution including their variances and covariances. We investigated the influence of extreme data points to the results by repeating the analyses with data where the observations with the lowest 2.5% and the highest 2.5% of forest bird abundance were removed.

### 2.4 Spatial Gompertz model for random species groups

We assessed the effectiveness of the titmouse group as an ecological indicator for forest bird density by evaluating the performance of titmice compared with randomly drawn species sets. Six species were randomly sampled from the observed forest bird data. The group of sampled species represented a control indicator group (referred to as control group hereafter) for the six titmouse species observed in the two countries. The forest birds for each control group comprised of all the other bird species in the community excluding the control group species. The analysis is computationally intensive, which is why we restricted the random sampling to 300 control groups for each country.

To facilitate comparisons among the performances of titmice and control groups, we only included the linear effect of a control group on forest bird density (excluding the species in the respective control group). Therefore, we first applied the spatial Gompertz model to estimate the effect of titmouse abundance on forest bird density without the quadratic term of titmice for both countries. The “titmouse model” for density of forest birds *d*(*s* *_i_* *, t* *_i_*) with a log‐linked linear predictor was identical to Equation 2, but eliminating the quadratic effect of titmice, *γ*_2_ = 0.

Prior to the analysis of each of the data sets with a randomly drawn control group, we removed samples where no forest birds were observed (<0.01% of all observations in both countries), to have a 100% bird encounter probability. Standardized control group abundance (given as biomass) and standardized environmental PC (i.e., PC1 or PC2; see Section 2.2) were used as varying among sites and years. The “control species model” for density of forest birds *d*(*s_i_*, *t_i_*) was again identical to Equation 2, but replacing variable *tit*(*s_i_*, *t_i_*) with control(*s_i_*, *t_i_*) and eliminating the quadratic effect of titmice, *γ*_2_ = 0. Spatial and spatio‐temporal variation in expected forest bird density *d*(*s_i_*, *t_i_*) for each, the titmouse group and the control groups, were modeled similarly as described above (Equations 3 and 4).

The use of some control groups did not result in model convergence, so we excluded the control groups with nonconverged models from further consideration. To infer statistical significance for each converged control group model, we compared the 95% confidence interval of the *γ*_4_ estimate of the control group to zero. We only compared the control group models with a statistically significant *γ*_4_ parameter estimate to the point estimate of *γ*_1_ (titmouse estimate). The *γ*_4_ estimates were considered to be significantly different from the *γ*_1_ estimate when the 95% confidence intervals of the *γ*_4_ estimate did not encompass the *γ*_1_ estimate. The R codes are provided in supplementary material (see R code 2 and 3).

## 3 RESULTS

### 3.1 Spatial Gompertz model for titmouse abundance and forest bird density

**FIGURE 1:**
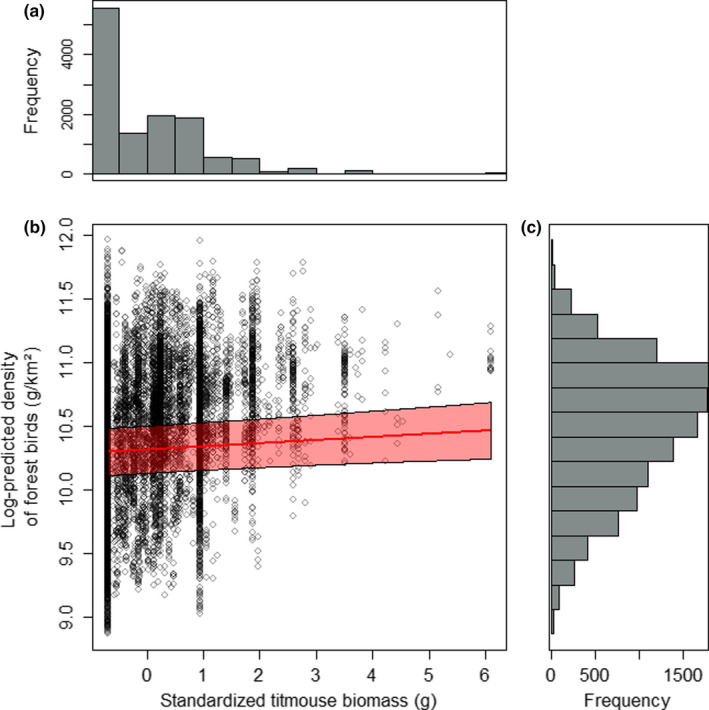
Frequency distribution of standardized titmouse abundance (given as biomass; g) in Finland (a). The relationship between log‐predicted density of forest birds (g/km^2^) and standardized titmouse abundance (given as biomass; g) in Finland in 2001 (i.e., first study year; *β* = 10.315, *γ*_1_ = 0.025; see Table 1 for definition of all symbols) (b). Circles are predicted forest bird densities for the sampling points and the fitted line with 95% confidence intervals derives from the spatial Gompertz model (see Section 2.3 for details) visualizing the linear relationship between predicted forest bird density and titmouse abundance. There was minor variance among years in the intercept (10.184 < *β* < 10.418), so the elevation of the line varies among years, but the slope remains the same. Frequency distribution of log‐predicted density of forest birds (g/km^2^) in Finland (c)

In Finland, the spatial Gompertz models including the linear and quadratic titmouse abundance effects were nearly equally good (ΔAIC = 2.0). However, as the quadratic term of titmouse abundance was not statistically significant (*γ*_2_ = −0.0005, [95% confidence interval: −0.013, 0.012]; see Appendix 1 for results of the quadratic model: Table A1) in the quadratic model, we base our inferences on the simpler (i.e., linear) model (Table 2; Figure 1). There was a positive association between titmouse abundance and forest bird density (Table 2; Figure 1). The effect of environmental PC was not different from zero at the 95% confidence level (Table 2). Additionally, standard deviations for both spatial (*σ_ω_*) and spatio‐temporal (*σ_ε_*) variation were high and clearly different from zero (Table 2). Spatial variation was stronger than spatio‐temporal variation over the study period (see also Appendix 1: Figure A3). We identified four structurally different habitat class‐specific subsets of data for Finland (deciduous, spruce and pine forest, and deciduous bush; Supporting Information 2: Table S2.1 and Figure S2.1). For these separate analyses, deciduous forest and spruce forest had a positive relationship between titmouse abundance and forest bird density, but the relationship was statistically significant only in deciduous forest (Supporting Information 2: Tables S2.3 and S2.4, and Figure S2.3). The models for deciduous bush and pine forest habitats did not converge, most likely due to the relatively small sizes of these data sets. The removal of extreme observations did not change the results (Supporting Information 1: Table S1.4 and Figure S1.6).

**TABLE 2:** Parameter estimates and their 95% confidence intervals for the model including only a linear relationship between titmouse abundance (measured in biomass) and forest bird density in Finland 2001–2013; parameter estimates and their 95% confidence limits (Lower/Upper 95% CI) for the effects of titmouse abundance (*γ*_1_; see Table 1), environmental PC (*γ*_3_), standard deviation of spatial variation (*σ_ω_*) and spatio‐temporal variation (*σ_ε_*)

Parameter	Estimate	Lower 95% CI	Upper 95% CI
**Titmouse abundance (*γ*_1_)**	**0.025**	**0.005**	**0.045**
Environmental PC (*γ*_3_)	0.005	−0.073	0.083
Standard deviation of spatial variation (*σ_ω_*)	1.810	1.489	2.131
Standard deviation of spatio‐temporal variation (*σ_ε_*)	0.447	0.336	0.557

Note

Parameter estimates are in log‐scale and parameters that are different from zero at 95% confidence level are highlighted in bold. Variance components are not highlighted because they are inevitably non‐negative.

**FIGURE 2:**
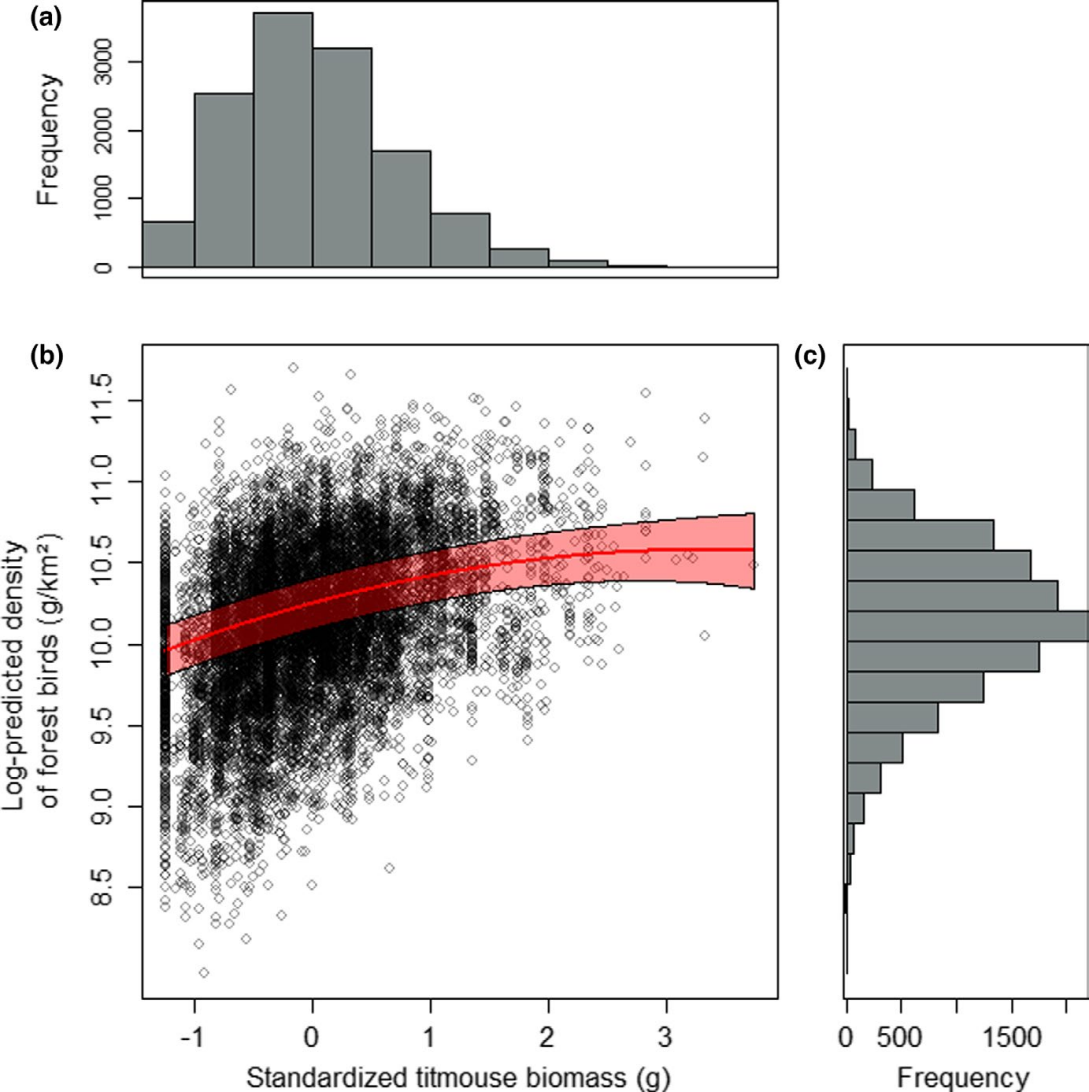
Frequency distribution of standardized titmouse abundance (given as biomass; g) in France (a). The relationship between log‐predicted density of forest birds (g/km^2^) and standardized titmouse abundance (given as biomass; g) in France in 2001 (i.e., first study year; *β* = 10.251, *γ*_1_ = 0.198, *γ*_2_ = −0.030; see Table 1 for definition of all symbols) (b). Circles are predicted forest bird densities for the sampling points and the fitted line with 95% confidence intervals derives from the spatial Gompertz model (see Section 2.3 for details) visualizing the quadratic relationship between predicted forest bird density and titmouse abundance. There was minor variance among years in the intercept (10.145 < *β* < 10.259), so the elevation of the line varies among years, but the curve remains the same. Frequency distribution of log‐predicted density of forest birds (g/km^2^) in France (c)

In France, the spatial Gompertz model including titmouse abundance, squared titmouse abundance, and environmental PC best fitted to the data as indicated by the AIC comparison (ΔAIC = 12.2; see Appendix 1 for results of the linear model: Table A2). The predicted forest bird density increased with titmouse abundance, the positive association becoming weaker toward higher titmouse abundance (Table 3; Figure 2). Standard deviation for spatial (*σ_ω_*) variation was higher than for spatio‐temporal (*σ_ε_*) variation in forest bird density (Table 3; see also Appendix 1: Figure A4). There was no association between forest bird density and environmental PC (Table 3). French data were classified into five structurally different habitat types (coniferous, mixed, deciduous and young forest, and coppice; Supporting Information 2: Table S2.2 and Figure S2.2). All habitat‐specific models that converged indicated a positive relationship between titmouse abundance and forest bird density (Supporting Information 2: Tables S2.5–S2.8 and Figures S2.4–S2.7). The linear relationship was statistically significant in coniferous and mixed forest, and the quadratic relationship in deciduous and young forest. The relatively small sizes of the data sets resulted in wide confidence intervals in each habitat type, excluding deciduous forest. The model for coppice did not converge, likely for the same reason. The results remained unchanged even when the observations with the lowest 2.5% and the highest 2.5% of forest bird abundance were removed (Supporting Information 1: Table S1.5 and Figure S1.7).

**TABLE 3:** Parameter estimates and their 95% confidence intervals for the model including a quadratic relationship between titmouse abundance (measured in biomass) and forest bird density in France 2001–2013; parameter estimates and their 95% confidence limits (Lower/Upper 95% CI) for the effects of titmouse abundance (*γ*_1_; see Table 1), quadratic term of titmouse abundance (*γ*_2_), environmental PC (*γ*_3_), standard deviation of spatial variation (*σ_ω_*), and spatio‐temporal variation (*σ_ε_*)

Parameter	Estimate	Lower 95% CI	Upper 95% CI
**Titmouse abundance (*γ*_1_)**	**0.198**	**0.173**	**0.222**
**[Titmouse abundance]^2^ (*γ*_2_)**	**−0.030**	**−0.045**	**−0.014**
Environmental PC (*γ*_3_)	−0.019	−0.047	0.008
Standard deviation of spatial variation (*σ_ω_*)	0.641	0.579	0.703
Standard deviation of spatio‐temporal variation (*σ_ε_*)	0.295	0.269	0.320

Note

Parameter estimates are in log‐scale and parameters that are different from zero at 95% confidence level are highlighted in bold. Variance components are not highlighted because they are inevitably non‐negative.

### 3.2 Spatial Gompertz models for random species groups

**FIGURE 3:**
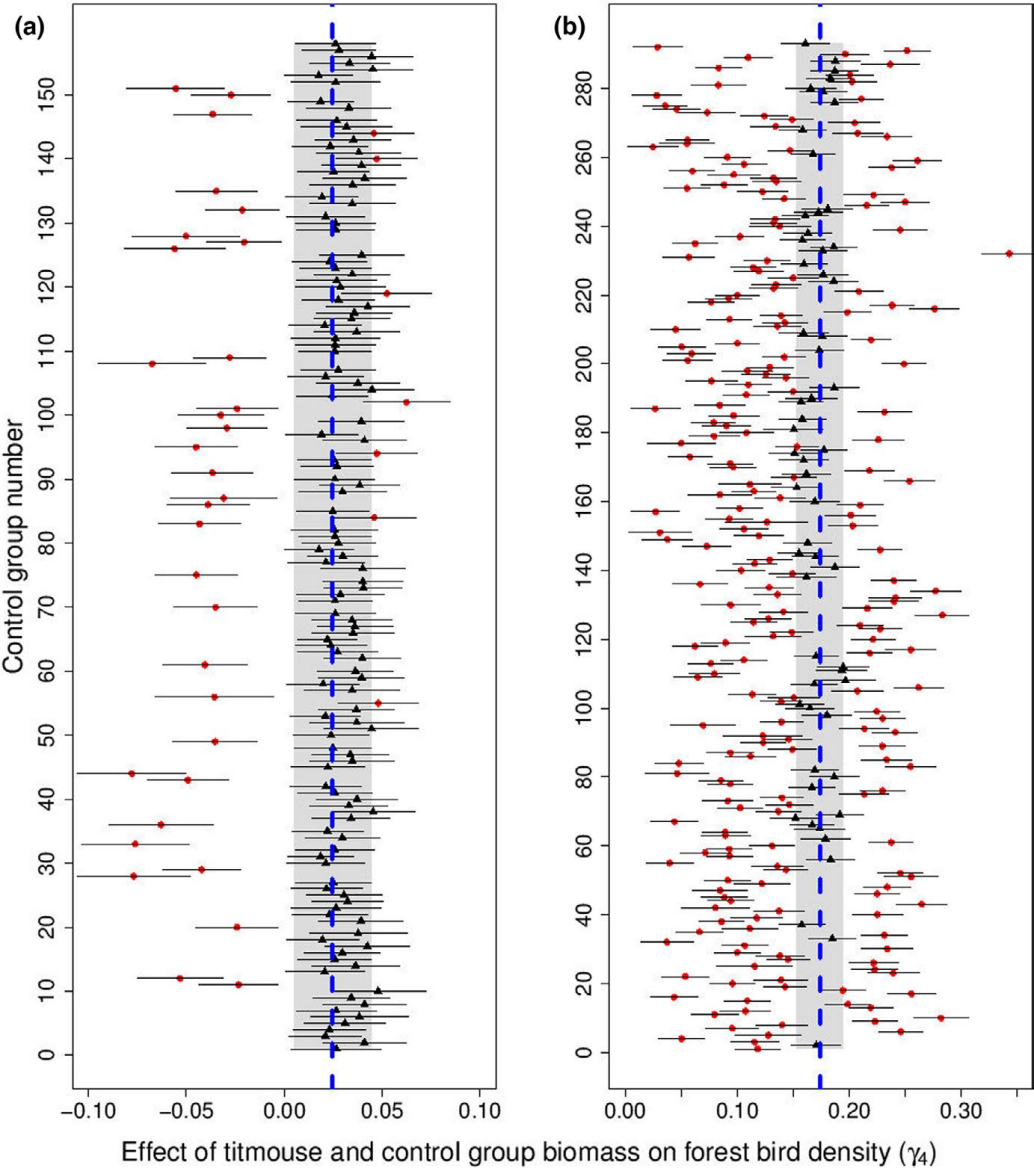
VAST estimates from the models that converged and had a significant parameter estimate (a: *n* = 158; b: *n* = 293) for the associations between abundance (given as biomass) of each randomly drawn control group and forest bird density (*γ*_4_) with error bars showing the 95% confidence intervals in Finland (a) and in France (b). Red filled circles represent those estimates that were statistically different from the titmouse estimate (*γ*_1_; a: *n* = 39; b: *n* = 235) and the black triangles depict those estimates that were not significantly different from the titmouse estimate (a: *n* = 119; b: *n* = 58) at 95% confidence level (see Section 2.4 for details). The estimate for the association between titmouse abundance (given as biomass) and forest bird density is shown with the blue dashed line (a: *γ*_1_ = 0.025; b: *γ*_1_ = 0.174), and the gray shaded area shows the 95% confidence intervals for the titmouse estimates

In Finland, 297 out of the 300 data sets with randomly drawn control groups resulted in model convergence. 158 parameter estimates of the association between abundance of the control group and density of forest birds were different from zero at the 95% confidence level (see Supporting Information 1 for a list of species and parameter estimates: Table S1.6). These associations between control groups and forest birds were overall positive (mean *γ*_4_ = 0.017, median *γ*_4_ = 0.026). Only seven (4.4%) out of these 158 significant models had in turn a significantly stronger positive association with forest birds than titmice and 32 (20.3%) control groups had a significantly weaker association (i.e., the 95% confidence intervals did not encompass the titmouse estimate; Figure 3a; Supporting Information 1: Table S1.6). The associations between the control groups and forest birds ranged between a minimum of *γ*_4_ = −0.078 and a maximum of *γ*_4_ = 0.062. In Finland, the control group that had the strongest positive association with the total density of forest birds consisted of *Muscicapa striata*, *Turdus pilaris*, *Picoides tridactylus*, *Cuculus canorus*, *Phylloscopus collybita*, and *Oriolus oriolus*.

In France, the analysis of the spatial Gompertz model including only linear titmouse effect on forest bird density resulted in a significantly positive titmouse covariate effect (*γ*_1_ = 0.174, [0.153, 0.195]; Appendix 1: Table A2). All 300 analyzed models for the control groups converged. 293 out of the models resulted in a statistically significant parameter estimate for the association between the control group and forest birds, and all considered associations were positive (mean *γ*_4_ = 0.147, median *γ*_4_ = 0.142; see Supporting Information 1 for a list of species and parameter estimates: Table S1.7). 24.2% (71) out of these 293 significant control groups had a significantly stronger and 56.0% (164) a significantly weaker association with forest birds than titmice (i.e., the 95% confidence intervals did not overlap with the titmouse estimate; Figure 3b; Supporting Information: Table S1.7). The estimates among the control groups ranged between a minimum of *γ*_4_ = 0.025 and a maximum of *γ*_4_ = 0.343. *Turdus philomelos*, *Fringilla coelebs*, *Turdus merula*, *Dendrocopos major*, *Cuculus canorus*, and *Phoenicurus ochruros* were the species of the best performing control group in France.

## 4 DISCUSSION

Bird density showed a positive association with titmouse abundance (given in biomass) in European forest bird communities when controlling for gradients in temperature and precipitation and spatio‐temporal autocorrelations. This positive relationship was linear in Finland, but nonlinear in France, where the positive association at low titmouse abundance leveled off at higher titmouse abundance. In Finland, titmice appear to be generally better indicators of forest bird density than groups of species drawn randomly from the same community. In France, many randomly drawn species groups were equally good or even better indicators of forest bird density than titmice. Overall, titmice seem to be a potential ecological indicator of forest bird density at the macroecological scale in Northern Europe, while the performance of the titmouse indicator group is not as clear in Western Europe.

There was a linear and positive association between titmouse abundance and forest bird density in Finland. This implies that shared habitat preferences as well as positive interspecific interactions, where forest birds choose a breeding habitat near titmouse species (i.e., heterospecific attraction), may underlie the result. Heterospecific attraction has been suggested to result in passerine aggregations in Northern Europe (Forsman et al., 2009; Mönkkönen et al., 1990; Thomson et al., 2003) and may be a consequence of acquiring and using interspecific social information on habitat quality (e.g., food availability or predator density) from species that breed earlier in the year (Forsman et al., 2002, 2009). The proportion of migratory species is higher in Northern Europe than in Western Europe (Newton, 2008), and thus, social information provided by titmice can be more important for migratory and later breeding birds in Finland than in France. While our results cannot prove heterospecific attraction, the results are consistent with the prediction of the heterospecific attraction hypothesis (Forsman et al., 2002, 2009; Mönkkönen et al., 1990, 1996, 2004). Furthermore, heterospecific attraction has mainly been studied among specific species, whereas here we studied the entire forest songbird community. However, as a result of a lower number of observations and a poorer spatial coverage of the data in Finland than in France, for example, a possible nonlinear association between titmice and forest birds in Finland may have remained unobserved.

The nonlinear, approximately asymptotic, relationship between titmouse abundance and forest bird density in France suggests that positive associations become weaker with increasing titmouse abundance. This pattern likely arises from stronger interspecific competition at high levels of titmouse abundance. Alternatively, the strength of the association could be explained by change in habitat quality. Titmice include both generalist and specialist species, and the generalists (e.g., great tit [*Parus major*]) may persist with higher abundances in habitats of lower quality (i.e., less resources), while other forest bird species may decline in the same conditions. Thus, the strength of the association between titmice and other forest birds may decrease after a certain threshold in the quality of the habitat. However, habitat quality variation is an unlikely explanation for our result because it is not likely that the highest observed abundances of titmice, where the association between titmice and other forest birds levels off, were observed at low‐quality habitats. Instead, it seems plausible that the highest titmouse abundances occur in high‐quality habitats. The asymptotic relationship between titmouse abundance and forest bird density reported here also parallels the low‐density end of the unimodal relationship between *Parus* and *Fringilla* species in Central Europe (Mönkkönen et al., 2004). Indeed, high densities of titmice could negatively affect the fitness of other passerines (Forsman et al., 2008; Gustafsson, 1987; Sasvári et al., 1987), leading to avoidance of habitats with high titmouse densities because of increased competition. Thus, interspecific competition is the most likely process leading to the observed asymptotic relationship between titmouse abundance and other forest bird density. Also, when using multiple species instead of a single one as an ecological indicator, the density of the indicator species within the community increases in relation to the target species. Therefore, density‐dependent factors should be considered when multiple species are used as an indicator.

Species densities are directly and indirectly affected by environmental factors. The overall lower titmouse densities in harsher environments in Northern Europe (Forsman & Mönkkönen, 2003) reduce competition even at high local titmouse densities. This may favor social information use (Forsman et al., 2009), where migrant species seek a breeding habitat with high resident titmouse densities. Higher productivity of the environment facilitates overall higher titmouse densities in Western and Central Europe than in Northern Europe (Forsman & Mönkkönen, 2003). Consequently, the negative effects of competition between titmice and forest birds may outweigh the positive effects of social information use at high densities (Mönkkönen et al., 2004), leading to weaker associations between titmice and forest birds in Western and Central Europe. However, spatial variation in temperature and precipitation (proxies of productivity) did not explain our results. Productivity varies a lot within Europe, increasing toward the south (Boisvenue & Running, 2006; Field et al., 1998), and densities of resident species are known to increase with higher temperature and precipitation (Forsman & Mönkkönen, 2003). Thus, we expected environmental principal component (PC) summarizing temperature and precipitation variation to be positively correlated with species densities. Nevertheless, environmental PC may only describe the potential environmental favorability at each location and the lack of this environmental effect could indicate missing variables that would describe the actual local environmental conditions. Our supplementary analysis (Supporting Information 2) for subsets of the data accounted for potentially important environmental factors affecting forest bird density, such as specific forest types (e.g., spruce, pine and broad‐leaved) or different tree heights (Fraixedas et al., 2015; Lehikoinen et al., 2017). Although the indicator value of titmice was the highest in deciduous forests, which reflects the main habitat preference of many titmouse species (del Hoyo et al., 2007), titmice had a positive relationship with forest bird density in all forest types and in the pooled data including all forest types. This suggests that titmice could be used as an indicator of forest bird density independently of forest type.

The use of indicator species to study population trends or biodiversity is a common practice but has received some criticism (Andelman & Fagan, 2000; Cabeza et al., 2008; Favreau et al., 2006). This emphasizes that selecting a suitable species or species group as a potential indicator requires careful consideration. In Finland, we found stronger evidence on the efficiency of titmice as an indicator group, when comparing the performance of titmice against randomly drawn species groups. There was an extremely low proportion of randomly drawn species groups (7 out of 158, i.e., 4.4%) performing significantly better than titmice. Hence, we found strong evidence for titmouse abundance to be a suitable indicator for total forest bird density in Northern Europe. In France, a large proportion (71 out of 293, i.e., 24.2%) of control groups outperformed the titmouse group as an indicator, suggesting that the relationship between titmouse abundance and total bird density is more complex in Western Europe.

Even though many of the randomly drawn species groups performed better as an indicator than titmice, in France, the species sets included in those random species groups cannot be observed as easily as titmice. Similarly, other commonly used indicator bird species groups, such as woodpeckers (Menon & Shahabuddin, 2021; Mikusiński et al., 2001) or cuckoos (Møller et al., 2017), are relatively less abundant and diverse, and more habitat specialized than many titmouse species, potentially making them less suitable indicators for forest bird abundances. Titmice have many features of a suitable ecological indicator group: cost‐efficient observations, well‐known biology, conspicuous behavior, almost global distribution (Caro & O'Doherty, 1999; del Hoyo et al., 2007; Gill et al., 2005; Landres et al., 1988), and ecological traits broadly overlapping with those of a wide range of the target species. Thus, using titmice as an indicator group seems practical, this practicality potentially outweighing the better performance of some random and other common species groups. Also, many resident titmouse populations have declined in Finland, and two earlier common species are now threatened (willow tit [*Poecile montanus*] and crested tit [*Lophophanes cristatus*]; Hyvärinen et al., 2019). In the light of our results, the decline in titmouse populations could be an early warning signal of a more widespread decline of forest bird populations in near future. Together the practicality and the wide distribution of titmice makes them potentially useful indicators in some biogeographical realms, yet caution is needed when extrapolating our results outside of Northern Europe.

## 5 CONCLUSIONS

In most of the current citizen science bird monitoring programs, observers must visually and acoustically identify all birds, demanding a high level of species identification skills. If overall forest bird densities could be estimated using titmouse abundance as an indicator, as suggested by our results, it would open the possibility to use also less‐experienced observers (i.e., observers with limited species identification skills) in citizen science to support current bird density estimation methods. This could increase the spatial and temporal extents of the current bird monitoring schemes, especially in Northern Europe. These cost‐effective data sets may increase the efficiency of planning conservation areas and actions, which is one of the most urgent global issues in applied ecology. The globally wide distribution and conspicuous behavior of the titmouse group may open new possibilities for planning forest bird conservation at a macroecological scale. More locally, focusing on titmice could be a cost‐effective approach to monitor the consequence of local disturbance or conservation plans. For instance, the level and velocity in the restoration of a given forested ecosystem (e.g., following fire or logging) could be reflected by the dynamics of titmouse populations more easily than using an exhaustive survey of the entire bird community.

## CONFLICT OF INTEREST

The authors declare that they have no known competing financial interests or personal relationships that could have appeared to influence the work reported in this paper.

## AUTHOR CONTRIBUTION

**Mira H. Kajanus:** Conceptualization (equal); Data curation (equal); Formal analysis (lead); Investigation (lead); Methodology (equal); Validation (lead); Visualization (equal); Writing – original draft (lead). **Jukka T. Forsman:** Conceptualization (equal); Funding acquisition (lead); Investigation (supporting); Project administration (lead); Supervision (lead); Writing – review & editing (lead). **Maximilian G. R. Vollstädt:** Conceptualization (supporting); Data curation (equal); Formal analysis (supporting); Investigation (equal); Methodology (equal); Visualization (equal); Writing – review & editing (equal). **Vincent Devictor:** Data curation (supporting); Resources (equal); Writing – review & editing (equal). **Merja Elo:** Conceptualization (supporting); Writing – review & editing (equal). **Aleksi Lehikoinen:** Data curation (supporting); Resources (equal); Writing – review & editing (equal). **Mikko Mönkkönen:** Conceptualization (supporting); Writing – review & editing (equal). **James T. Thorson:** Conceptualization (supporting); Methodology (lead); Software (lead); Supervision (supporting); Writing – review & editing (equal). **Sami M. Kivelä:** Conceptualization (equal); Data curation (equal); Methodology (equal); Project administration (equal); Supervision (lead); Visualization (supporting); Writing – review & editing (lead).

### OPEN RESEARCH BADGES

This article has earned an Open Data Badges for making publicly available the digitally‐shareable data necessary to reproduce the reported results. The data is available at https://doi.org/10.5061/dryad.cjsxksn6x.

## Supporting information

Supplementary Material

Supplementary Material

Supplementary Material

Supplementary Material

Supplementary Material

Supplementary Material

## Data Availability

Data for the main analyses are available in Dryad Digital Repository (https://doi.org/10.5061/dryad.cjsxksn6x). Data sets for the analyses of the randomly drawn species groups are available upon request from the corresponding author. Metadata (Metadata) and R codes (R code [Link], [Link], [Link]) are provided in the Supporting Information.
