# Supplementary material for: Titmice are a better indicator of bird density in Northern European than in Western European forests
Source: Ecol Evol. 2022 Feb 12;12(2):e8479. doi: 10.1002/ece3.8479 (PMC8840900; doi:10.1002/ece3.8479)
Supplement: Supplementary file 1 — Supplementary Material [file ECE3-12-e8479-s006.docx]

**Supporting Information 1.**

Kajanus, M.H., Forsman, J.T., Vollstädt, M.G.R., Devictor, V., Elo, M., Lehikoinen, A., Mönkkönen, M., Thorson, J.T., and Kivelä, S.M. 2021. Titmice are a better indicator of bird density in Northern European than in Western European forests. *Ecology and Evolution*.

**Table S1.1.** List of scientific name, English name and species code of the species observed in Finland and France. Seven species of titmice are listed at the top of the table.

| SCIENTIFIC NAME | ENGLISH NAME | SPP. CODE | FINLAND | FRANCE |
| --- | --- | --- | --- | --- |
| Titmouse species |  |  |  |  |
| *Cyanistes caeruleus* | Eurasian Blue Tit | PARCAE | x | x |
| *Lophophanes cristatus* | European Crested Tit | PARCRI | x | x |
| *Parus cinctus* | Siberian Tit | PARCIN | x |  |
| *Parus major* | Great Tit | PARMAJ | x | x |
| *Poecile montanus* | Willow Tit | PARMON | x | x |
| *Periparus ater* | Coal Tit | PARATE | x | x |
| *Poecile palustris* | Marsh Tit | PARPAL |  | x |
| Other species |  |  |  |  |
| *Acrocephalus dumetorum* | Blyth's Reed-warbler | ACRDUM | x |  |
| *Aegithalos caudatus* | Long-tailed Tit | AEGCAU | x | x |
| *Anthus trivialis* | Tree Pipit | ANTTRI | x | x |
| *Apus apus* | Common Swift | APUAPU | x | x |
| *Bombycilla garrulus* | Bohemian Waxwing | BOMGAR | x |  |
| *Caprimulgus europaeus* | European Nightjar | CAPEUR | x |  |
| *Carduelis chloris* | European Greenfinch | CARCHL | x | x |
| *Carduelis flammea* | Common Redpoll | CARMEA | x |  |
| *Carduelis spinus* | Eurasian Siskin | CARSPI | x |  |
| *Carpodacus erythrinus* | Common Rosefinch | CARERY | x |  |
| *Certhia brachydactyla* | Short-toed Treecreeper | CERBRA |  | x |
| *Certhia familiaris* | Eurasian Treecreeper | CERFAM | x | x |
| *Cinclus cinclus* | White-throated Dipper | CINCIN | x |  |
| *Coccothraustes coccothraustes* | Hawfinch | COCCOC |  | x |
| *Columba oenas* | Stock Dove | COLOEN | x | x |
| *Columba palumbus* | Common Wood-Pigeon | COLPAL | x | x |
| *Corvus corax* | Common Raven | CORRAX | x |  |
| *Corvus corone* | Hooded Crow | CORONE | x | x |
| *Corvus frugilegus* | Rook | CORFRU |  | x |
| *Corvus monedula* | Eurasian Jackdaw | CORMON | x | x |
| *Cuculus canorus* | Common Cuckoo | CUCCAN | x | x |
| *Dendrocopos leucotos* | White-backed Woodpecker | DENLEU |  |  |
| *Dendrocopos major* | Great Spotted Woodpecker | DENMAJ | x | x |
| *Dendrocopos medius* | Middle Spotted Woodpecker | DENMED |  | x |
| *Dendrocopos minor* | Lesser Spotted Woodpecker | DENMIN | x | x |
| *Dryocopus martius* | Black Woodpecker | DRYMAR | x | x |
| *Emberiza hortulana* | Ortolan Bunting | EMBHOR | x |  |
| *Emberiza pusilla* | Little Bunting | EMBPUS | x |  |
| *Emberiza rustica* | Rustic Bunting | EMBRUS | x |  |
| *Erithacus rubecula* | European Robin | ERIRUB | x | x |
| *Ficedula hypoleuca* | European Pied Flycatcher | FICHYP | x |  |
| *Ficedula parva* | Red-brested Flycatcher | FICPAR | x |  |
| *Fringilla coelebs* | Common Chaffinch | FRICOE | x | x |
| *Fringilla montifringilla* | Brambling | FRIMON | x |  |
| *Garrulus glandarius* | Eurasian Jay | GARGLA | x | x |
| *Hippolais icterina* | Icterine Warbler | HIPICT | x |  |
| *Hippolais polyglotta* | Melodious Warbler | HIPPOL |  | x |
| *Jynx torquilla* | Eurasian Wryneck | JYNTOR | x | x |
| *Lanius collurio* | Red-backed Shrike | LANCOL | x | x |
| *Lanius excubitor* | Great-grey Shrike | LANEXC | x |  |
| *Loxia curvirostra* | Red Crossbill | LOXCUR | x | x |
| *Loxia leucoptera* | Two-barred Crossbill | LOXLEU | x |  |
| *Loxia pytyopsittacus* | Parrot Crossbill | LOXPYT | x |  |
| *Lullula arborea* | Wood Lark | LULARB | x | x |
| *Luscinia luscinia* | Thrush Nightingale | LUSLUS | x |  |
| *Luscinia megarhynchos* | Common Nightingale | LUSMEG |  | x |
| *Luscinia svecica* | Bluethroat | LUSSVE | x |  |
| *Motacilla cinerea* | Gray Wagtail | MOTCIN |  | x |
| *Muscicapa striata* | Spotted Flycatcher | MUSSTR | x | x |
| *Nucifraga caryocatactes* | Spotted Nutcracker | NUCCAR | x |  |
| *Oriolus oriolus* | Eurasian Golden Oriole | ORIORI | x | x |
| *Passer montanus* | Eurasian Tree Sparrow | PASMON | x | x |
| *Perisoreus infaustus* | Siberian Jay | PERINF | x |  |
| *Phoenicurus ochruros* | Black Redstart | PHOOCH |  | x |
| *Phoenicurus phoenicurus* | Common Redstart | PHOPHO | x | x |
| *Phylloscopus bonelli* | Western Bonelli's Warbler | PHYBON |  | x |
| *Phylloscopus borealis* | Arctic Warbler | PHYBOR | x |  |
| *Phylloscopus collybita* | Common Chiffchaff | PHYCOL | x | x |
| *Phylloscopus sibilatrix* | Wood Warbler | PHYSIB | x | x |
| *Phylloscopus trochiloides* | Greenish Warbler | PHYDES | x |  |
| *Phylloscopus trochilus* | Willow Warbler | PHYLUS | x | x |
| *Pica pica* | Eurasian Magpie | PICPIC | x | x |
| *Picoides tridactylus* | Eurasian three-toed Woodpecker | PICTRI | x |  |
| *Picus canus* | Grey-headed Woodpecker | PICCAN | x |  |
| *Picus viridis* | Eurasian Green Woodpecker | PICVIR |  | x |
| *Pinicola enucleator* | Pine Grosbeak | PINENU | x |  |
| *Prunella modularis* | Dunnock | PRUMOD | x | x |
| *Pyrrhula pyrrhula* | Eurasian Bullfinch | PYRPYR | x | x |
| *Regulus ignicapilla* | Firecrest | REGIGN |  | x |
| *Regulus regulus* | Goldcrest | REGREG | x | x |
| *Serinus serinus* | European Serin | SERSER |  | x |
| *Sitta europaea* | Eurasian Nuthatch | SITEUR |  | x |
| *Streptopelia turtur* | European Turtle-Dove | STRTUR |  | x |
| *Sturnus vulgaris* | European Starling | STUVUL | x | x |
| *Sylvia atricapilla* | Eurasian Blackcap | SYLATR | x | x |
| *Sylvia borin* | Garden Warbler | SYLBOR | x | x |
| *Sylvia cantillans* | Subalpine Warbler | SYLCAN |  | x |
| *Sylvia curruca* | Lesser Whitethroat | SYLCUR | x | x |
| *Sylvia melanocephala* | Sardinian Warbler | SYLMEL |  | x |
| *Tarsiger cyanurus* | Red-flanked Bluetail | TARCYA | x |  |
| *Troglodytes troglodytes* | Eurasian Wren | TROTRO | x | x |
| *Turdus iliacus* | Redwing | TURILI | x |  |
| *Turdus merula* | Eurasian Blackbird | TURMER | x | x |
| *Turdus philomelos* | Song Thrush | TURPHI | x | x |
| *Turdus pilaris* | Fieldfare | TURPIL | x | x |
| *Turdus viscivorus* | Mistle Thrush | TURVIS | x | x |
| *Upupa epops* | Eurasian Hoopoe | UPUEPO |  | x |

**Table S1.2.** Forest types that were included in the analysis in each country.

| COUNTRY | FOREST TYPE |
| --- | --- |
| Finland | Spruce forest |
|  | Pine forest |
|  | Young coniferous forest |
|  | Deciduous forest |
|  | Mixed forest |
|  | Pine swamp |
|  | Deciduous bush |
|  |  |
| France | Coniferous forest |
|  | Deciduous forest |
|  | Mixed forest |
|  | Coppice |
|  | Young forest |

**Table S1.3.** Results of the sensitivity analysis on the number (*x*) of spatial locations (“knots”; *s*) set in VAST for analyzing French forest bird data. The table shows the parameter estimate for the three covariates (titmouse abundance ${(\gamma}_{1})$, quadratic term of titmouse abundance $(\gamma_{2})$ and environmental PC $(\gamma_{3})$), standard deviation of spatial variation (*σ_ω_*), standard deviation of spatio-temporal variation (*σ_ε_*) and their standard errors in parentheses.

| Number of knots | Titmouse estimate | [Titmouse]^2^ estimate | Environmental PC estimate | Standard deviation of spatial variation | Standard deviation of spatio-temporal variation |
| --- | --- | --- | --- | --- | --- |
| 1500* | 0.152 (0.010) | -0.025 (0.006) | -0.024 (0.014) | 0.697 (0.027) | 0.303 (0.012) |
| 1400 | 0.159 (0.011) | -0.026 (0.007) | -0.021 (0.014) | 0.691 (0.028) | 0.302 (0.013) |
| 1300 | 0.174 (0.011) | -0.027 (0.007) | -0.018 (0.014) | 0.697 (0.031) | 0.310 (0.013) |
| 1200 | 0.183 (0.011) | -0.028 (0.007) | -0.018 (0.014) | 0.686 (0.030) | 0.304 (0.013) |
| 1100 | 0.193 (0.012) | -0.031 (0.007) | -0.038 (0.014) | 0.678 (0.028) | 0.292 (0.012) |
| 1000 | **0.198 (0.012)** | **-0.030 (0.008)** | **-0.019 (0.014)** | **0.641 (0.032)** | **0.295 (0.013)** |
| 900 | 0.214 (0.013) | -0.031 (0.008) | -0.039 (0.014) | 0.684 (0.036) | 0.299 (0.015) |
| 800 | 0.219 (0.013) | -0.033 (0.009) | -0.019 (0.015) | 0.602 (0.030) | 0.282 (0.012) |
| 500 | 0.236 (0.016) | -0.024 (0.011) | -0.032 (0.015) | 0.465 (0.030) | 0.256 (0.011) |

*Notes*: Model that was used for inferences is highlighted in **bold**.

**Table S1.4.** Model results for analysis of the Finnish data, where 2.5% of the lowest and the highest forest bird abundance data points have been removed. Parameter estimates and their 95% confidence intervals for the model including only a linear relationship between titmouse abundance (measured as biomass) and forest bird density in Finland 2001–2013; parameter estimates and their 95% confidence limits (LOWER/UPPER 95% CI) of the effects of titmouse abundance ($\gamma_{1}$; see Table 1), environmental PC $(\gamma_{3})$, standard deviation of spatial variation (*σ_ω_*) and spatio-temporal variation (*σ_ε_*) on forest bird density.

| PARAMETER | ESTIMATE | LOWER 95% CI | UPPER 95% CI |
| --- | --- | --- | --- |
| Titmouse abundance (γ_1_) | 0.026 | 0.008 | 0.045 |
| Environmental PC (γ_3_) | 0.011 | -0.065 | 0.087 |
| Standard deviation of spatial variation (*σ_ω_*) | 1.685 | 1.252 | 2.117 |
| Standard deviation of spatio-temporal variation (*σ_ε_*) | 0.446 | 0.306 | 0.586 |

*Notes*: Parameter estimates are in log-scale and parameters that are different from zero at 95% confidence level are highlighted in **bold**. Variance components are not highlighted because they are inevitably non-negative.

**Table S1.5.** Model results for analysis of the French data, where 2.5% of the lowest and the highest forest bird abundance data points have been removed. Parameter estimates and their 95% confidence intervals for the model including a quadratic relationship between titmouse abundance (measured as biomass) and forest bird density in France 2001–2013; parameter estimates and their 95% confidence limits (LOWER/UPPER 95% CI) for the effects of titmouse abundance ($\gamma_{1}$; see Table 1), quadratic term of titmouse abundance ${(\gamma}_{2})$, environmental PC $(\gamma_{3})$, standard deviation of spatial variation (*σ_ω_*) and spatio-temporal variation (*σ_ε_*) on forest bird density.

| PARAMETER | ESTIMATE | LOWER 95% CI | UPPER 95% CI |
| --- | --- | --- | --- |
| Titmouse abundance (γ_1_) | **0.148** | **0.126** | **0.169** |
| [Titmouse abundance]^2^ (γ_2_) | **-0.023** | **-0.037** | **-0.008** |
| Environmental PC (γ_3_) | -0.014 | -0.040 | 0.012 |
| Standard deviation of spatial variation (*σ_ω_*) | 0.514 | 0.464 | 0.565 |
| Standard deviation of spatio-temporal variation (*σ_ε_*) | 0.234 | 0.212 | 0.256 |

*Notes*: Parameter estimates are in log-scale and parameters that are different from zero at 95% confidence level are highlighted in **bold**. Variance components are not highlighted because they are inevitably non-negative.

**Table S1.6.** List of randomly drawn species (SP. 1-6), estimates (EST.) and their 95% confidence intervals (LOW CI/UPP CI) for the effect of the control group abundance (measured in biomass) on forest bird density ${(\gamma}_{4})$ in Finland in all converged and statistically significant (i.e. models with estimates different from the titmouse estimate $\gamma_{1}$ at the 95% confidence level) control groups (*n* = 158). Linear effect of titmouse abundance on forest bird density ${(\gamma}_{1})$ is on the top of the table.

| MODEL | SP. 1 | SP. 2 | SP. 3 | SP. 4 | SP. 5 | SP. 6 | EST. | LOW CI | UPP CI |
| --- | --- | --- | --- | --- | --- | --- | --- | --- | --- |
| Titmice | PARATE | PARCAE | PARCRI | PARMAJ | PARMON | PARPAL | 0,025 | 0,005 | 0,045 |
|  |  |  |  |  |  |  |  |  |  |
| 1 | TURVIS | PHYSIB | EMBHOR | DENMIN | CUCCAN | REGREG | 0,027 | 0,004 | 0,050 |
| 2 | CINCIN | JYNTOR | TURPIL | CAPEUR | PICCAN | SYLATR | 0,041 | 0,020 | 0,062 |
| 3 | BOMGAR | COLOEN | PICCAN | LUSLUS | REGREG | AEGCAU | 0,021 | 0,003 | 0,039 |
| 4 | PHYSIB | LUSSVE | COLOEN | ACRDUM | LULARB | PHYDES | 0,023 | 0,005 | 0,041 |
| 5 | ERIRUB | COLOEN | PHYCOL | PICTRI | CARSPI | COLPAL | 0,031 | 0,010 | 0,052 |
| 6 | CARMEA | CINCIN | HIPICT | PHYLUS | PHYCOL | PERINF | 0,038 | 0,014 | 0,063 |
| 7 | TROTRO | CARERY | LOXPYT | COLPAL | FRIMON | LOXLEU | 0,027 | 0,006 | 0,047 |
| 8 | PHYDES | TURPIL | EMBHOR | PICTRI | TARCYA | PICPIC | 0,041 | 0,020 | 0,062 |
| 9 | HIPICT | PICCAN | CORONE | FRIMON | COLOEN | LULARB | 0,034 | 0,015 | 0,054 |
| 10 | PHYBOR | LANEXC | PHYDES | CERFAM | FICHYP | PHYLUS | 0,048 | 0,024 | 0,072 |
| 11 | AEGCAU | CARSPI | PASMON | MUSSTR | PHOPHO | PRUMOD | -0,023 | -0,043 | -0,003 |
| 12 | ERIRUB | FRIMON | TARCYA | SYLCUR | FICPAR | PRUMOD | -0,053 | -0,075 | -0,031 |
| 13 | TARCYA | PRUMOD | TURMER | LUSLUS | CARERY | STUVUL | 0,021 | 0,001 | 0,041 |
| 14 | TARCYA | CUCCAN | APUAPU | BOMGAR | EMBRUS | EMBHOR | 0,036 | 0,014 | 0,059 |
| 15 | CORONE | LOXPYT | TURVIS | COLPAL | JYNTOR | ANTTRI | 0,026 | 0,007 | 0,045 |
| 16 | PRUMOD | LULARB | STUVUL | APUAPU | LUSLUS | HIPICT | 0,030 | 0,010 | 0,049 |
| 17 | APUAPU | CORONE | TURMER | CUCCAN | PRUMOD | DENMAJ | 0,042 | 0,021 | 0,064 |
| 18 | PICCAN | PINENU | PHYDES | CARMEA | ACRDUM | DENMAJ | 0,020 | 0,001 | 0,038 |
| 19 | PHYLUS | CARMEA | NUCCAR | PASMON | LULARB | FICPAR | 0,038 | 0,013 | 0,063 |
| 20 | PYRPYR | TURPHI | LOXLEU | DENMAJ | LOXPYT | LOXCUR | -0,024 | -0,045 | -0,003 |
| 21 | PHOPHO | CARERY | PHYCOL | ACRDUM | TURPIL | AEGCAU | 0,039 | 0,018 | 0,061 |
| 22 | CARERY | STUVUL | CORMON | PHYDES | COLOEN | PASMON | 0,023 | 0,004 | 0,043 |
| 23 | TURPHI | CARCHL | CORRAX | COLPAL | CAPEUR | FRICOE | 0,027 | 0,004 | 0,050 |
| 24 | CINCIN | EMBHOR | LANEXC | COLPAL | STUVUL | CORRAX | 0,032 | 0,014 | 0,051 |
| 25 | CARCHL | CARERY | EMBHOR | DENMAJ | EMBPUS | LUSLUS | 0,031 | 0,011 | 0,050 |
| 26 | TARCYA | PYRPYR | LUSLUS | PHYBOR | CAPEUR | COLOEN | 0,022 | 0,003 | 0,040 |
| 27 | BOMGAR | ANTTRI | HIPICT | TURILI | STUVUL | GARGLA | 0,025 | 0,006 | 0,044 |
| 28 | CARSPI | FICHYP | FRICOE | PHYBOR | TROTRO | ANTTRI | -0,077 | -0,106 | -0,048 |
| 29 | PHYCOL | FICPAR | PRUMOD | ERIRUB | CINCIN | PYRPYR | -0,042 | -0,062 | -0,022 |
| 30 | CARERY | GARGLA | FICHYP | EMBRUS | PERINF | CORMON | 0,021 | 0,002 | 0,041 |
| 31 | CARSPI | ACRDUM | REGREG | STUVUL | LANEXC | PHYSIB | 0,019 | 0,002 | 0,035 |
| 32 | STUVUL | CARCHL | TURILI | REGREG | AEGCAU | CORMON | 0,026 | 0,006 | 0,046 |
| 33 | LUSSVE | LANCOL | FRICOE | FICPAR | CARERY | TURPHI | -0,076 | -0,103 | -0,049 |
| 34 | LUSSVE | COLOEN | LUSLUS | CARMEA | PINENU | APUAPU | 0,030 | 0,011 | 0,049 |
| 35 | STUVUL | PERINF | ANTTRI | PICPIC | BOMGAR | MUSSTR | 0,022 | 0,004 | 0,040 |
| 36 | NUCCAR | ACRDUM | PERINF | EMBRUS | TROTRO | FRICOE | -0,063 | -0,090 | -0,036 |
| 37 | CORONE | LANEXC | PYRPYR | ANTTRI | PRUMOD | EMBPUS | 0,034 | 0,014 | 0,054 |
| 38 | CARCHL | PERINF | STUVUL | CUCCAN | BOMGAR | DENMAJ | 0,045 | 0,024 | 0,067 |
| 39 | LOXLEU | EMBPUS | GARGLA | CORONE | PRUMOD | SYLBOR | 0,033 | 0,013 | 0,053 |
| 40 | PINENU | CORONE | LUSSVE | PASMON | PHYLUS | LOXLEU | 0,037 | 0,017 | 0,058 |
| 41 | LANEXC | PASMON | DENMAJ | PICCAN | CARCHL | SYLCUR | 0,026 | 0,007 | 0,045 |
| 42 | COLOEN | PINENU | PYRPYR | LOXPYT | NUCCAR | DENMIN | 0,021 | 0,003 | 0,039 |
| 43 | TURPHI | TURVIS | CARMEA | PHYBOR | REGREG | BOMGAR | -0,049 | -0,070 | -0,028 |
| 44 | REGREG | LOXLEU | BOMGAR | CAPEUR | FRICOE | CARSPI | -0,078 | -0,105 | -0,050 |
| 45 | PICTRI | LULARB | GARGLA | EMBPUS | DENMAJ | TROTRO | 0,022 | 0,004 | 0,041 |
| 46 | ORIORI | TURPIL | SYLCUR | BOMGAR | CAPEUR | CORMON | 0,035 | 0,013 | 0,056 |
| 47 | CARSPI | EMBRUS | GARGLA | LOXLEU | MUSSTR | CORONE | 0,034 | 0,014 | 0,053 |
| 48 | COLOEN | REGREG | PICTRI | SYLATR | APUAPU | TURILI | 0,025 | 0,005 | 0,045 |
| 49 | ERIRUB | CAPEUR | TURPHI | NUCCAR | DENMAJ | PASMON | -0,035 | -0,057 | -0,014 |
| 50 | APUAPU | GARGLA | CARSPI | LANEXC | PICPIC | PHYSIB | 0,024 | 0,005 | 0,043 |
| 51 | CAPEUR | LOXLEU | PHYLUS | PHYBOR | LUSLUS | EMBHOR | 0,045 | 0,021 | 0,069 |
| 52 | EMBRUS | LUSSVE | CERFAM | REGREG | PHYLUS | ACRDUM | 0,037 | 0,013 | 0,061 |
| 53 | LANEXC | AEGCAU | TURVIS | PICPIC | STUVUL | MUSSTR | 0,021 | 0,003 | 0,039 |
| 54 | APUAPU | SYLATR | PICTRI | STUVUL | CORONE | LOXCUR | 0,037 | 0,018 | 0,056 |
| 55 | EMBRUS | TURPIL | STUVUL | PHOPHO | GARGLA | PICCAN | 0,048 | 0,027 | 0,068 |
| 56 | CAPEUR | PHYDES | LOXPYT | EMBHOR | FRIMON | AEGCAU | -0,036 | -0,066 | -0,005 |
| 57 | CORONE | TURPIL | SYLATR | TURILI | LULARB | FRICOE | 0,035 | 0,011 | 0,059 |
| 58 | LULARB | COLOEN | REGREG | LANEXC | CARSPI | MUSSTR | 0,020 | 0,002 | 0,038 |
| 59 | LANEXC | ANTTRI | PRUMOD | TURPIL | PHOPHO | PICPIC | 0,040 | 0,018 | 0,061 |
| 60 | PHYSIB | DENMIN | DRYMAR | CORONE | PASMON | PINENU | 0,036 | 0,017 | 0,056 |
| 61 | PICCAN | SYLATR | PASMON | PRUMOD | CARCHL | TURPHI | -0,041 | -0,062 | -0,019 |
| 62 | PHYBOR | CARERY | DRYMAR | STUVUL | PHYLUS | LOXCUR | 0,040 | 0,020 | 0,059 |
| 63 | PICPIC | COLPAL | CERFAM | PHYCOL | SYLCUR | FICHYP | 0,027 | 0,007 | 0,048 |
| 64 | LULARB | DENMAJ | APUAPU | ERIRUB | STUVUL | FRIMON | 0,024 | 0,006 | 0,042 |
| 65 | DENMIN | CORRAX | EMBRUS | SYLATR | CORONE | COLPAL | 0,022 | 0,007 | 0,037 |
| 66 | STUVUL | EMBHOR | LUSLUS | APUAPU | DENMIN | TARCYA | 0,035 | 0,014 | 0,056 |
| 67 | APUAPU | CARCHL | CERFAM | CORONE | SYLATR | LOXLEU | 0,036 | 0,017 | 0,055 |
| 68 | TURVIS | TURPHI | CORONE | PYRPYR | FRIMON | ACRDUM | 0,035 | 0,014 | 0,055 |
| 69 | LUSSVE | COLPAL | ERIRUB | EMBRUS | REGREG | BOMGAR | 0,026 | 0,006 | 0,046 |
| 70 | EMBRUS | TURPHI | CARCHL | PRUMOD | LOXCUR | LUSLUS | -0,035 | -0,056 | -0,014 |
| 71 | LULARB | FICHYP | JYNTOR | BOMGAR | DRYMAR | PICPIC | 0,026 | 0,007 | 0,045 |
| 72 | REGREG | CAPEUR | LUSSVE | PICCAN | TARCYA | CUCCAN | 0,029 | 0,006 | 0,051 |
| 73 | TURILI | STUVUL | PICTRI | LOXPYT | TURVIS | CORONE | 0,040 | 0,020 | 0,060 |
| 74 | CORONE | ACRDUM | CARSPI | TURILI | PHYSIB | PICTRI | 0,040 | 0,020 | 0,060 |
| 75 | LUSSVE | BOMGAR | PHOPHO | CARMEA | ERIRUB | SYLBOR | -0,045 | -0,066 | -0,024 |
| 76 | LULARB | TURPIL | APUAPU | PHOPHO | ERIRUB | CERFAM | 0,040 | 0,019 | 0,062 |
| 77 | TROTRO | PICPIC | CERFAM | DENMAJ | MUSSTR | FRIMON | 0,021 | 0,002 | 0,041 |
| 78 | CARERY | LUSLUS | BOMGAR | STUVUL | DRYMAR | LULARB | 0,030 | 0,012 | 0,048 |
| 79 | CORONE | SYLCUR | CORRAX | CARCHL | LOXLEU | CORMON | 0,018 | 0,000 | 0,035 |
| 80 | CARSPI | COLOEN | AEGCAU | FICHYP | NUCCAR | DRYMAR | 0,028 | 0,009 | 0,046 |
| 81 | CARMEA | EMBRUS | COLOEN | EMBHOR | PHYSIB | CARCHL | 0,026 | 0,007 | 0,045 |
| 82 | TURPHI | PASMON | PICCAN | TURPIL | LOXPYT | GARGLA | 0,026 | 0,004 | 0,048 |
| 83 | LUSSVE | CARERY | TROTRO | PICTRI | TURPHI | LOXPYT | -0,043 | -0,064 | -0,022 |
| 84 | TURPIL | PERINF | DENMAJ | CAPEUR | EMBPUS | PHYBOR | 0,046 | 0,025 | 0,067 |
| 85 | PERINF | TROTRO | ORIORI | FICHYP | DRYMAR | APUAPU | 0,025 | 0,006 | 0,043 |
| 86 | CINCIN | DENMIN | TURPHI | CARCHL | CARMEA | ORIORI | -0,039 | -0,060 | -0,018 |
| 87 | DENMIN | FRICOE | TURMER | CAPEUR | NUCCAR | PHYCOL | -0,031 | -0,058 | -0,004 |
| 88 | LOXCUR | COLOEN | PHYLUS | TURMER | REGREG | PHYDES | 0,030 | 0,008 | 0,052 |
| 89 | PHYCOL | PYRPYR | TURPIL | APUAPU | CORONE | LOXPYT | 0,039 | 0,018 | 0,059 |
| 90 | NUCCAR | FICHYP | COLPAL | ACRDUM | LOXLEU | ERIRUB | 0,026 | 0,005 | 0,046 |
| 91 | ERIRUB | CARERY | ANTTRI | PHOPHO | SYLCUR | EMBHOR | -0,037 | -0,057 | -0,016 |
| 92 | REGREG | CAPEUR | CORONE | COLPAL | SYLATR | FICPAR | 0,027 | 0,009 | 0,045 |
| 93 | NUCCAR | DRYMAR | PHYBOR | JYNTOR | PICCAN | DENMAJ | 0,025 | 0,007 | 0,044 |
| 94 | DENMAJ | SYLATR | PHYCOL | EMBRUS | PHYLUS | APUAPU | 0,047 | 0,027 | 0,068 |
| 95 | NUCCAR | CAPEUR | LUSLUS | CARMEA | TURPHI | PHYBOR | -0,045 | -0,066 | -0,024 |
| 96 | NUCCAR | PASMON | LOXLEU | SYLATR | PHYBOR | TURPIL | 0,041 | 0,020 | 0,062 |
| 97 | DENMAJ | SYLCUR | CERFAM | PASMON | JYNTOR | EMBRUS | 0,019 | 0,001 | 0,038 |
| 98 | PYRPYR | LANEXC | LOXLEU | REGREG | ANTTRI | JYNTOR | -0,029 | -0,050 | -0,009 |
| 99 | CUCCAN | FICPAR | EMBRUS | PICPIC | PICCAN | TURMER | 0,039 | 0,017 | 0,061 |
| 100 | PICCAN | JYNTOR | PERINF | CERFAM | FRIMON | ANTTRI | -0,032 | -0,054 | -0,011 |
| 101 | PHOPHO | CAPEUR | CARERY | ANTTRI | LANCOL | PRUMOD | -0,024 | -0,045 | -0,003 |
| 102 | **MUSSTR** | **TURPIL** | **PICTRI** | **CUCCAN** | **PHYCOL** | **ORIORI** | **0,062** | **0,040** | **0,084** |
| 103 | CARERY | SYLBOR | STUVUL | JYNTOR | ACRDUM | PICCAN | 0,024 | 0,006 | 0,043 |
| 104 | SYLATR | DRYMAR | TURPIL | CORONE | TURMER | PERINF | 0,045 | 0,024 | 0,066 |
| 105 | LANCOL | CORONE | PHYLUS | PHYSIB | ERIRUB | CARCHL | 0,038 | 0,017 | 0,059 |
| 106 | LOXCUR | PICPIC | LULARB | TROTRO | APUAPU | BOMGAR | 0,021 | 0,002 | 0,040 |
| 107 | LULARB | CARCHL | JYNTOR | ACRDUM | STUVUL | CARMEA | 0,028 | 0,009 | 0,046 |
| 108 | PHYBOR | FRICOE | PRUMOD | JYNTOR | PICCAN | PICTRI | -0,067 | -0,095 | -0,040 |
| 109 | PRUMOD | EMBHOR | ERIRUB | TURVIS | LANEXC | LUSLUS | -0,028 | -0,046 | -0,009 |
| 110 | CARSPI | CORONE | COLPAL | CORMON | REGREG | CARERY | 0,026 | 0,007 | 0,044 |
| 111 | PINENU | CAPEUR | COLPAL | FRIMON | CINCIN | PHYCOL | 0,026 | 0,006 | 0,046 |
| 112 | PASMON | LUSSVE | CUCCAN | LOXPYT | ANTTRI | GARGLA | 0,026 | 0,003 | 0,049 |
| 113 | COLPAL | DENMAJ | PICCAN | JYNTOR | CUCCAN | CARCHL | 0,037 | 0,015 | 0,059 |
| 114 | NUCCAR | PICTRI | HIPICT | EMBRUS | DENMAJ | LANCOL | 0,021 | 0,002 | 0,039 |
| 115 | CINCIN | CORONE | PHYCOL | PYRPYR | EMBPUS | BOMGAR | 0,034 | 0,015 | 0,054 |
| 116 | ACRDUM | CORONE | PINENU | LUSLUS | COLOEN | PASMON | 0,036 | 0,017 | 0,055 |
| 117 | CINCIN | PHYSIB | PICPIC | TURPIL | FICPAR | LOXCUR | 0,043 | 0,021 | 0,064 |
| 118 | FICHYP | COLOEN | CARCHL | CINCIN | DENMIN | PHYBOR | 0,028 | 0,009 | 0,046 |
| 119 | TURPIL | CUCCAN | TROTRO | DENMIN | LOXPYT | CORMON | 0,052 | 0,030 | 0,075 |
| 120 | EMBRUS | SYLBOR | CAPEUR | LANCOL | PICTRI | CUCCAN | 0,029 | 0,006 | 0,052 |
| 121 | DRYMAR | CARCHL | REGREG | LUSLUS | CORMON | TURMER | 0,027 | 0,006 | 0,047 |
| 122 | TROTRO | CAPEUR | APUAPU | COLOEN | DENMAJ | CERFAM | 0,035 | 0,016 | 0,054 |
| 123 | LOXCUR | NUCCAR | PHYCOL | CINCIN | STUVUL | LULARB | 0,026 | 0,008 | 0,044 |
| 124 | LUSLUS | DRYMAR | PHOPHO | REGREG | PASMON | PICPIC | 0,023 | 0,004 | 0,042 |
| 125 | LUSLUS | CARMEA | PHYSIB | TROTRO | TURPIL | DENMIN | 0,040 | 0,018 | 0,061 |
| 126 | GARGLA | BOMGAR | FRICOE | TROTRO | TURPHI | CAPEUR | -0,056 | -0,082 | -0,030 |
| 127 | FICPAR | PYRPYR | PINENU | CARMEA | LUSSVE | CERFAM | -0,020 | -0,039 | -0,001 |
| 128 | SYLCUR | FRICOE | NUCCAR | CARCHL | PHYSIB | CAPEUR | -0,050 | -0,077 | -0,023 |
| 129 | LOXLEU | TARCYA | MUSSTR | EMBHOR | CARCHL | NUCCAR | 0,026 | 0,006 | 0,046 |
| 130 | COLPAL | PICCAN | TURVIS | PYRPYR | ORIORI | LANEXC | 0,026 | 0,006 | 0,047 |
| 131 | LUSSVE | CORMON | CORONE | CORRAX | CAPEUR | FRICOE | 0,021 | 0,001 | 0,041 |
| 132 | JYNTOR | BOMGAR | CERFAM | ORIORI | CARSPI | EMBRUS | -0,021 | -0,040 | -0,003 |
| 133 | PHYCOL | FICHYP | GARGLA | CUCCAN | COLPAL | ACRDUM | 0,035 | 0,013 | 0,057 |
| 134 | SYLATR | LUSLUS | CARSPI | MUSSTR | PHYCOL | DRYMAR | 0,019 | 0,001 | 0,038 |
| 135 | LANCOL | LULARB | PRUMOD | CARMEA | PHOPHO | CAPEUR | -0,035 | -0,055 | -0,014 |
| 136 | STUVUL | FICHYP | PHYBOR | CUCCAN | ANTTRI | MUSSTR | 0,035 | 0,013 | 0,057 |
| 137 | PICPIC | LULARB | EMBHOR | TURPIL | PASMON | DENMIN | 0,041 | 0,020 | 0,062 |
| 138 | CARCHL | PHYCOL | PRUMOD | PASMON | PINENU | STUVUL | 0,025 | 0,007 | 0,044 |
| 139 | CAPEUR | COLOEN | TURPIL | CORONE | PINENU | LANEXC | 0,039 | 0,019 | 0,060 |
| 140 | EMBPUS | ORIORI | PICTRI | COLOEN | TURPIL | CARCHL | 0,047 | 0,026 | 0,068 |
| 141 | CORMON | SYLATR | CUCCAN | TROTRO | PICPIC | HIPICT | 0,038 | 0,017 | 0,060 |
| 142 | CERFAM | EMBRUS | ACRDUM | STUVUL | CORMON | LANEXC | 0,024 | 0,004 | 0,043 |
| 143 | LANEXC | CORONE | LANCOL | CARMEA | FICHYP | ACRDUM | 0,035 | 0,016 | 0,055 |
| 144 | PASMON | SYLCUR | GARGLA | TURPIL | PHOPHO | DRYMAR | 0,046 | 0,025 | 0,066 |
| 145 | ORIORI | CARSPI | COLPAL | TURMER | PHYLUS | TARCYA | 0,032 | 0,009 | 0,055 |
| 146 | MUSSTR | COLPAL | CAPEUR | AEGCAU | LUSLUS | FICPAR | 0,027 | 0,007 | 0,047 |
| 147 | FICHYP | EMBHOR | AEGCAU | ERIRUB | PHYDES | CARSPI | -0,036 | -0,056 | -0,017 |
| 148 | PHYLUS | LUSLUS | PYRPYR | TARCYA | SYLBOR | STUVUL | 0,033 | 0,011 | 0,055 |
| 149 | PHYSIB | PYRPYR | LOXLEU | REGREG | STUVUL | JYNTOR | 0,019 | 0,002 | 0,035 |
| 150 | LULARB | ANTTRI | DENMIN | CAPEUR | LOXLEU | PICTRI | -0,027 | -0,047 | -0,007 |
| 151 | FICPAR | EMBPUS | PRUMOD | TURVIS | FICHYP | FRICOE | -0,055 | -0,080 | -0,030 |
| 152 | ORIORI | DENMIN | HIPICT | PICCAN | CUCCAN | PHOPHO | 0,026 | 0,003 | 0,049 |
| 153 | SYLATR | JYNTOR | LULARB | CAPEUR | STUVUL | FRIMON | 0,018 | 0,000 | 0,035 |
| 154 | REGREG | TURPIL | TROTRO | DRYMAR | CAPEUR | JYNTOR | 0,045 | 0,025 | 0,066 |
| 155 | COLOEN | PYRPYR | LULARB | GARGLA | COLPAL | DENMIN | 0,033 | 0,013 | 0,054 |
| 156 | DRYMAR | TURPIL | ERIRUB | LULARB | PHYBOR | EMBHOR | 0,045 | 0,024 | 0,065 |
| 157 | COLOEN | LOXCUR | JYNTOR | PICPIC | LOXPYT | LANCOL | 0,028 | 0,009 | 0,047 |
| 158 | COLPAL | TROTRO | PERINF | CARSPI | AEGCAU | FRIMON | 0,026 | 0,006 | 0,047 |

*Notes*: Parameter estimates are in log-scale and the control group with the highest parameter estimate (i.e. with the best predictive power for forest bird density) is marked in **bold** (Model 102; see a key for species codes in Table S1.1).

**Table S1.7.** List of randomly drawn species (SP. 1-6), estimates (EST.) and their 95% confidence intervals (LOW CI/UPP CI) for the effect of the control group abundance (measured in biomass) on forest bird density ${(\gamma}_{4})$ in France in all converged and statistically significant (i.e. models with estimates different from the titmouse estimate $\gamma_{1}$ at the 95% confidence level) control groups (*n* = 293). Linear effect of titmouse abundance on forest bird density ${(\gamma}_{1})$ is on the top of the table.

| MODEL | SP. 1 | SP. 2 | SP. 3 | SP. 4 | SP. 5 | SP. 6 | EST. | LOW CI | UPP CI |
| --- | --- | --- | --- | --- | --- | --- | --- | --- | --- |
| Titmice | PARATE | PARCAE | PARCRI | PARMAJ | PARMON | PARPAL | 0,174 | 0,153 | 0,195 |
|  |  |  |  |  |  |  |  |  |  |
| 1 | PYRPYR | PHYSIB | ANTTRI | PICPIC | CERFAM | STRTUR | 0,118 | 0,098 | 0,139 |
| 2 | CORMON | PICPIC | MOTCIN | SYLATR | STRTUR | GARGLA | 0,171 | 0,149 | 0,193 |
| 3 | MUSSTR | CARCHL | SYLBOR | PHYCOL | PHOOCH | PRUMOD | 0,116 | 0,094 | 0,137 |
| 4 | LANCOL | LOXCUR | CORMON | SITEUR | PHYLUS | CERBRA | 0,050 | 0,029 | 0,071 |
| 5 | SYLATR | LANCOL | CORMON | FRICOE | COCCOC | ANTTRI | 0,128 | 0,098 | 0,157 |
| 6 | STUVUL | SYLCAN | COLPAL | CUCCAN | ORIORI | CARCHL | 0,246 | 0,226 | 0,266 |
| 7 | SYLATR | TURVIS | COCCOC | CORMON | PHYBON | APUAPU | 0,096 | 0,072 | 0,119 |
| 8 | TURPHI | STUVUL | CORMON | CARCHL | TURPIL | PHOPHO | 0,140 | 0,117 | 0,163 |
| 9 | ORIORI | PHYSIB | SERSER | COLPAL | STRTUR | MOTCIN | 0,223 | 0,204 | 0,243 |
| 10 | DENMED | LUSMEG | TURMER | CORMON | DENMAJ | CUCCAN | 0,282 | 0,258 | 0,307 |
| 11 | PYRPYR | UPUEPO | DENMED | SYLCUR | APUAPU | LUSMEG | 0,080 | 0,059 | 0,101 |
| 12 | ERIRUB | CORMON | COLOEN | UPUEPO | STUVUL | APUAPU | 0,107 | 0,085 | 0,130 |
| 13 | STRTUR | COLPAL | SYLCAN | HIPPOL | CERFAM | PHOPHO | 0,219 | 0,200 | 0,239 |
| 14 | APUAPU | ORIORI | PRUMOD | SITEUR | AEGCAU | CUCCAN | 0,199 | 0,179 | 0,219 |
| 15 | PICPIC | SERSER | STUVUL | PHYSIB | APUAPU | LOXCUR | 0,109 | 0,089 | 0,129 |
| 16 | CORMON | ANTTRI | UPUEPO | MOTCIN | AEGCAU | PHOOCH | 0,044 | 0,023 | 0,065 |
| 17 | TURPIL | PHYCOL | SYLMEL | TURMER | COLPAL | PHYSIB | 0,256 | 0,234 | 0,277 |
| 18 | CUCCAN | TURPHI | PHYLUS | SYLCUR | PHOOCH | TURPIL | 0,194 | 0,174 | 0,215 |
| 19 | TURVIS | PHYBON | JYNTOR | PASMON | CERBRA | CORONE | 0,143 | 0,124 | 0,162 |
| 20 | HIPPOL | CARCHL | REGIGN | STRTUR | REGREG | PHYSIB | 0,096 | 0,075 | 0,116 |
| 21 | ERIRUB | AEGCAU | CORMON | FRICOE | COCCOC | STRTUR | 0,139 | 0,115 | 0,163 |
| 22 | JYNTOR | LULARB | SYLMEL | SYLBOR | PRUMOD | SYLCAN | 0,054 | 0,032 | 0,075 |
| 23 | REGIGN | LULARB | FRICOE | TROTRO | ERIRUB | SYLBOR | 0,239 | 0,216 | 0,263 |
| 24 | TURPHI | CERBRA | PHYSIB | UPUEPO | COLPAL | JYNTOR | 0,223 | 0,203 | 0,243 |
| 25 | CORMON | TURMER | PRUMOD | CORFRU | SYLMEL | TROTRO | 0,116 | 0,082 | 0,150 |
| 26 | SYLATR | DENMED | PHYSIB | ORIORI | HIPPOL | SITEUR | 0,222 | 0,200 | 0,244 |
| 27 | SYLMEL | SITEUR | HIPPOL | TURVIS | PYRPYR | CORONE | 0,145 | 0,126 | 0,165 |
| 28 | GARGLA | COLOEN | LULARB | JYNTOR | PHYLUS | CERFAM | 0,138 | 0,117 | 0,160 |
| 29 | SYLMEL | PHYLUS | PHYSIB | SITEUR | REGREG | UPUEPO | 0,100 | 0,079 | 0,121 |
| 30 | CORMON | CUCCAN | UPUEPO | TURMER | PHOOCH | STRTUR | 0,234 | 0,211 | 0,257 |
| 31 | STUVUL | PRUMOD | TURPIL | COCCOC | LANCOL | DRYMAR | 0,107 | 0,086 | 0,128 |
| 32 | CORFRU | CERBRA | PHOPHO | PICVIR | PHOOCH | ERIRUB | 0,037 | 0,014 | 0,061 |
| 33 | TURMER | SYLCAN | SYLCUR | REGREG | CERBRA | CORONE | 0,185 | 0,164 | 0,206 |
| 34 | LOXCUR | COLPAL | TURPHI | CARCHL | CUCCAN | UPUEPO | 0,231 | 0,211 | 0,252 |
| 35 | TURVIS | SITEUR | DENMIN | PHOPHO | LULARB | CORMON | 0,066 | 0,045 | 0,088 |
| 36 | SYLBOR | DENMED | LUSMEG | HIPPOL | PHYCOL | COLOEN | 0,111 | 0,086 | 0,136 |
| 37 | TROTRO | TURPIL | UPUEPO | PICVIR | PHYBON | CUCCAN | 0,158 | 0,137 | 0,178 |
| 38 | AEGCAU | UPUEPO | SERSER | DENMIN | PICPIC | PHYCOL | 0,086 | 0,066 | 0,106 |
| 39 | SYLBOR | ANTTRI | AEGCAU | PRUMOD | LUSMEG | PHYBON | 0,118 | 0,095 | 0,140 |
| 40 | STUVUL | SYLBOR | SYLATR | MUSSTR | PHYLUS | TURPHI | 0,225 | 0,203 | 0,248 |
| 41 | SERSER | FRICOE | PHOPHO | TURVIS | SYLCAN | ERIRUB | 0,138 | 0,115 | 0,160 |
| 42 | CORFRU | PHYCOL | PRUMOD | FRICOE | CUCCAN | LOXCUR | 0,081 | 0,050 | 0,111 |
| 43 | SYLMEL | TURMER | CUCCAN | SYLBOR | PHYCOL | LANCOL | 0,265 | 0,243 | 0,287 |
| 44 | SYLMEL | SYLCAN | PHYLUS | DENMED | STRTUR | MOTCIN | 0,094 | 0,074 | 0,115 |
| 45 | PICVIR | CORMON | CARCHL | REGREG | MUSSTR | DRYMAR | 0,089 | 0,068 | 0,109 |
| 46 | COLPAL | DENMED | UPUEPO | REGIGN | SITEUR | STRTUR | 0,225 | 0,205 | 0,245 |
| 47 | PHOPHO | DENMED | REGIGN | COLOEN | LUSMEG | LANCOL | 0,085 | 0,060 | 0,110 |
| 48 | DENMIN | ERIRUB | CUCCAN | SYLCUR | TURPHI | HIPPOL | 0,234 | 0,213 | 0,255 |
| 49 | COCCOC | SERSER | DENMAJ | MOTCIN | SYLCUR | COLOEN | 0,122 | 0,098 | 0,147 |
| 50 | REGIGN | PHOPHO | SERSER | LULARB | MOTCIN | STRTUR | 0,092 | 0,071 | 0,112 |
| 51 | COLOEN | SYLATR | CUCCAN | SYLBOR | FRICOE | MOTCIN | 0,256 | 0,232 | 0,280 |
| 52 | MOTCIN | PYRPYR | COLPAL | DENMAJ | PHOPHO | GARGLA | 0,246 | 0,226 | 0,266 |
| 53 | CORONE | PRUMOD | REGREG | PHYBON | ORIORI | COCCOC | 0,144 | 0,124 | 0,163 |
| 54 | TROTRO | LANCOL | LOXCUR | PHYBON | PYRPYR | SYLCUR | 0,136 | 0,113 | 0,159 |
| 55 | PHOPHO | REGIGN | REGREG | SERSER | DRYMAR | PASMON | 0,040 | 0,019 | 0,061 |
| 56 | ERIRUB | MUSSTR | UPUEPO | TURPHI | LOXCUR | PHYCOL | 0,184 | 0,162 | 0,205 |
| 57 | PRUMOD | MUSSTR | PHYLUS | LULARB | PHYSIB | STRTUR | 0,093 | 0,073 | 0,114 |
| 58 | PHYLUS | TURVIS | COLOEN | REGIGN | LOXCUR | PHOPHO | 0,072 | 0,049 | 0,094 |
| 59 | SERSER | PRUMOD | SYLMEL | CERFAM | PHOPHO | STRTUR | 0,093 | 0,073 | 0,113 |
| 60 | GARGLA | ANTTRI | LANCOL | PASMON | LUSMEG | DENMED | 0,131 | 0,111 | 0,152 |
| 61 | MUSSTR | COLPAL | STRTUR | CERBRA | SYLCAN | PICVIR | 0,238 | 0,218 | 0,257 |
| 62 | TURVIS | SERSER | ERIRUB | TROTRO | DENMAJ | STRTUR | 0,179 | 0,157 | 0,201 |
| 63 | SERSER | ORIORI | REGIGN | SYLBOR | CORMON | TURVIS | 0,090 | 0,068 | 0,112 |
| 64 | AEGCAU | PICVIR | ANTTRI | REGREG | PYRPYR | CERBRA | 0,089 | 0,069 | 0,109 |
| 65 | AEGCAU | HIPPOL | MOTCIN | CERBRA | MUSSTR | FRICOE | 0,174 | 0,151 | 0,196 |
| 66 | PYRPYR | CORONE | JYNTOR | ORIORI | AEGCAU | GARGLA | 0,167 | 0,148 | 0,186 |
| 67 | SYLCAN | TURPIL | SYLBOR | SYLMEL | DRYMAR | COCCOC | 0,044 | 0,023 | 0,065 |
| 68 | LOXCUR | COCCOC | TROTRO | JYNTOR | ORIORI | SYLCAN | 0,152 | 0,130 | 0,174 |
| 69 | PHYBON | CUCCAN | HIPPOL | TURMER | PHOPHO | CORONE | 0,192 | 0,171 | 0,213 |
| 70 | SYLCAN | LANCOL | PRUMOD | PHYSIB | STRTUR | DENMAJ | 0,137 | 0,116 | 0,157 |
| 71 | CERBRA | CARCHL | UPUEPO | SERSER | STRTUR | REGREG | 0,103 | 0,082 | 0,123 |
| 72 | COLOEN | STRTUR | SYLCUR | CUCCAN | UPUEPO | APUAPU | 0,147 | 0,126 | 0,167 |
| 73 | DRYMAR | TURPIL | CERFAM | DENMIN | FRICOE | LULARB | 0,092 | 0,069 | 0,114 |
| 74 | LUSMEG | SYLCAN | PICPIC | CORONE | PHYLUS | SYLCUR | 0,140 | 0,121 | 0,159 |
| 75 | LUSMEG | TROTRO | TURPHI | ERIRUB | AEGCAU | HIPPOL | 0,214 | 0,192 | 0,235 |
| 76 | PICVIR | PRUMOD | PHOPHO | TROTRO | COLPAL | ORIORI | 0,230 | 0,210 | 0,250 |
| 77 | DENMED | TURPHI | SERSER | APUAPU | STRTUR | SYLMEL | 0,167 | 0,146 | 0,188 |
| 78 | APUAPU | MOTCIN | PICPIC | ERIRUB | ANTTRI | SYLCUR | 0,094 | 0,074 | 0,114 |
| 79 | SYLBOR | HIPPOL | PHOOCH | DENMIN | REGIGN | PICVIR | 0,086 | 0,066 | 0,106 |
| 80 | SITEUR | GARGLA | FRICOE | PICPIC | LUSMEG | SYLATR | 0,187 | 0,165 | 0,209 |
| 81 | DRYMAR | CORFRU | PYRPYR | MUSSTR | SYLBOR | TURPHI | 0,046 | 0,018 | 0,075 |
| 82 | SITEUR | PHOPHO | PICVIR | PHYLUS | TROTRO | GARGLA | 0,169 | 0,148 | 0,191 |
| 83 | HIPPOL | CERBRA | TURMER | LUSMEG | PICPIC | DENMAJ | 0,255 | 0,232 | 0,278 |
| 84 | LOXCUR | SYLCAN | REGIGN | DENMED | PHOOCH | DRYMAR | 0,048 | 0,026 | 0,070 |
| 85 | GARGLA | MOTCIN | TURMER | COLOEN | SYLCUR | APUAPU | 0,234 | 0,211 | 0,256 |
| 86 | DRYMAR | DENMAJ | SITEUR | PHOOCH | JYNTOR | HIPPOL | 0,112 | 0,090 | 0,134 |
| 87 | LANCOL | UPUEPO | HIPPOL | SITEUR | ANTTRI | APUAPU | 0,094 | 0,073 | 0,115 |
| 88 | HIPPOL | DRYMAR | SITEUR | PHYCOL | DENMED | CUCCAN | 0,149 | 0,128 | 0,171 |
| 89 | TROTRO | COLPAL | SYLCUR | PYRPYR | ERIRUB | PHYLUS | 0,230 | 0,209 | 0,250 |
| 90 | MOTCIN | AEGCAU | SYLATR | CERBRA | PICVIR | SYLCAN | 0,123 | 0,103 | 0,144 |
| 91 | STRTUR | PICPIC | DRYMAR | ERIRUB | COCCOC | DENMED | 0,146 | 0,125 | 0,167 |
| 92 | TURMER | PHYCOL | TROTRO | COCCOC | PASMON | CORFRU | 0,123 | 0,088 | 0,158 |
| 93 | PYRPYR | ERIRUB | COLPAL | PASMON | GARGLA | DRYMAR | 0,241 | 0,221 | 0,261 |
| 94 | TURMER | HIPPOL | LANCOL | JYNTOR | PICPIC | PHYBON | 0,214 | 0,192 | 0,236 |
| 95 | PHOPHO | TURPHI | CORFRU | JYNTOR | LULARB | PICVIR | 0,069 | 0,040 | 0,099 |
| 96 | DENMED | MUSSTR | UPUEPO | CARCHL | CORONE | LANCOL | 0,140 | 0,121 | 0,159 |
| 97 | SYLATR | AEGCAU | COLPAL | DENMED | ORIORI | SITEUR | 0,230 | 0,210 | 0,250 |
| 98 | DENMIN | GARGLA | MUSSTR | DRYMAR | STUVUL | SYLATR | 0,180 | 0,159 | 0,202 |
| 99 | COLPAL | PHOPHO | PYRPYR | LULARB | ERIRUB | CERFAM | 0,225 | 0,205 | 0,245 |
| 100 | CERFAM | GARGLA | TURPIL | PHYLUS | DENMAJ | PHYCOL | 0,165 | 0,144 | 0,187 |
| 101 | COCCOC | PICPIC | PHYBON | TURPIL | SYLATR | GARGLA | 0,156 | 0,135 | 0,177 |
| 102 | COCCOC | JYNTOR | SITEUR | PHYBON | TURPIL | CORONE | 0,139 | 0,120 | 0,158 |
| 103 | PHYCOL | TURVIS | CORFRU | CORONE | SYLCUR | HIPPOL | 0,151 | 0,128 | 0,173 |
| 104 | DENMIN | STUVUL | CERBRA | MUSSTR | SYLCUR | REGIGN | 0,114 | 0,093 | 0,135 |
| 105 | REGREG | FRICOE | DENMAJ | ERIRUB | LANCOL | CERBRA | 0,207 | 0,184 | 0,231 |
| 106 | REGREG | CUCCAN | JYNTOR | TURMER | UPUEPO | SYLBOR | 0,262 | 0,240 | 0,284 |
| 107 | STRTUR | SYLATR | REGIGN | CORONE | DENMIN | PICVIR | 0,169 | 0,148 | 0,189 |
| 108 | PHYSIB | HIPPOL | SYLCUR | COLOEN | SYLATR | ERIRUB | 0,197 | 0,170 | 0,224 |
| 109 | PRUMOD | DENMED | ANTTRI | AEGCAU | DRYMAR | CERBRA | 0,065 | 0,043 | 0,087 |
| 110 | DENMIN | REGREG | DENMAJ | SERSER | SYLMEL | CORMON | 0,080 | 0,057 | 0,102 |
| 111 | AEGCAU | SYLATR | PRUMOD | CERFAM | ANTTRI | PHOPHO | 0,194 | 0,171 | 0,216 |
| 112 | SYLCAN | PHYSIB | REGIGN | PHYCOL | FRICOE | REGREG | 0,195 | 0,172 | 0,218 |
| 113 | TURVIS | HIPPOL | DENMED | PHYLUS | PHYCOL | SYLCAN | 0,076 | 0,056 | 0,097 |
| 114 | DENMED | SYLCUR | LULARB | STRTUR | TURPIL | SITEUR | 0,106 | 0,085 | 0,127 |
| 115 | TURVIS | TURPHI | DENMIN | PHYSIB | CORONE | PICVIR | 0,171 | 0,151 | 0,190 |
| 116 | PHOOCH | PRUMOD | COLPAL | SITEUR | ERIRUB | DENMED | 0,218 | 0,198 | 0,238 |
| 117 | DENMAJ | PYRPYR | ANTTRI | TURMER | LULARB | SYLBOR | 0,255 | 0,232 | 0,278 |
| 118 | PICPIC | SYLCUR | APUAPU | SERSER | CORMON | MOTCIN | 0,062 | 0,042 | 0,083 |
| 119 | COLOEN | PICPIC | PHYCOL | CERBRA | PHOPHO | PHOOCH | 0,090 | 0,068 | 0,111 |
| 120 | REGIGN | MOTCIN | PASMON | TURPIL | COLPAL | PICPIC | 0,221 | 0,202 | 0,241 |
| 121 | LULARB | PASMON | CORMON | CARCHL | TURPHI | CERBRA | 0,132 | 0,108 | 0,157 |
| 122 | SYLCAN | PICVIR | LULARB | COLOEN | CORONE | REGIGN | 0,149 | 0,130 | 0,168 |
| 123 | HIPPOL | CUCCAN | COLPAL | AEGCAU | LUSMEG | SYLMEL | 0,228 | 0,209 | 0,247 |
| 124 | MUSSTR | PYRPYR | CUCCAN | TURPHI | CERFAM | PHYSIB | 0,210 | 0,189 | 0,230 |
| 125 | PICPIC | JYNTOR | PYRPYR | STRTUR | HIPPOL | SERSER | 0,115 | 0,094 | 0,135 |
| 126 | REGIGN | SYLATR | PICPIC | LUSMEG | PRUMOD | PHYCOL | 0,128 | 0,107 | 0,149 |
| 127 | LUSMEG | TURMER | HIPPOL | TURPHI | FRICOE | DENMIN | 0,283 | 0,259 | 0,307 |
| 128 | STRTUR | SITEUR | ORIORI | ANTTRI | JYNTOR | COCCOC | 0,141 | 0,120 | 0,163 |
| 129 | AEGCAU | TURPHI | HIPPOL | SYLCUR | CERBRA | SYLATR | 0,216 | 0,194 | 0,238 |
| 130 | LANCOL | PASMON | COLOEN | SITEUR | PHOOCH | PHYSIB | 0,094 | 0,068 | 0,121 |
| 131 | TURMER | PRUMOD | ORIORI | LOXCUR | PASMON | APUAPU | 0,240 | 0,218 | 0,262 |
| 132 | TURMER | TROTRO | HIPPOL | APUAPU | JYNTOR | COLOEN | 0,242 | 0,218 | 0,265 |
| 133 | LULARB | SYLMEL | STUVUL | PICVIR | ANTTRI | COCCOC | 0,136 | 0,115 | 0,157 |
| 134 | CORMON | SYLATR | TURMER | COLPAL | DENMIN | DENMAJ | 0,277 | 0,255 | 0,300 |
| 135 | SYLCUR | SYLBOR | CERFAM | GARGLA | PHYCOL | CORMON | 0,129 | 0,107 | 0,150 |
| 136 | AEGCAU | PASMON | ANTTRI | CERBRA | COCCOC | PHYBON | 0,067 | 0,043 | 0,091 |
| 137 | COLOEN | PICPIC | SYLATR | MUSSTR | COLPAL | GARGLA | 0,240 | 0,219 | 0,260 |
| 138 | COLOEN | CARCHL | LOXCUR | JYNTOR | SYLATR | COCCOC | 0,162 | 0,136 | 0,188 |
| 139 | PRUMOD | SYLATR | PASMON | ORIORI | PICVIR | PHOPHO | 0,150 | 0,129 | 0,170 |
| 140 | TURPIL | PRUMOD | UPUEPO | MUSSTR | TROTRO | REGIGN | 0,103 | 0,082 | 0,125 |
| 141 | HIPPOL | ANTTRI | DENMED | STUVUL | MUSSTR | TURPHI | 0,187 | 0,166 | 0,209 |
| 142 | UPUEPO | MUSSTR | ORIORI | SYLCAN | PICPIC | ANTTRI | 0,116 | 0,096 | 0,135 |
| 143 | PYRPYR | LUSMEG | PHYBON | GARGLA | LULARB | LANCOL | 0,129 | 0,109 | 0,149 |
| 144 | TURPHI | ERIRUB | SYLATR | CORONE | PHYSIB | SYLCAN | 0,170 | 0,150 | 0,191 |
| 145 | SITEUR | COLOEN | ORIORI | CORONE | CUCCAN | CORMON | 0,155 | 0,136 | 0,175 |
| 146 | SITEUR | PHYBON | ORIORI | SYLBOR | COLPAL | REGIGN | 0,228 | 0,208 | 0,247 |
| 147 | COCCOC | TURVIS | PHOPHO | CERFAM | PHYCOL | REGREG | 0,073 | 0,052 | 0,094 |
| 148 | JYNTOR | FRICOE | GARGLA | DENMED | TURPIL | CARCHL | 0,163 | 0,141 | 0,185 |
| 149 | AEGCAU | PYRPYR | PHYCOL | CUCCAN | APUAPU | CORFRU | 0,038 | 0,015 | 0,060 |
| 150 | ORIORI | SYLCAN | REGREG | DRYMAR | PHOPHO | ERIRUB | 0,120 | 0,098 | 0,142 |
| 151 | PHYCOL | CORFRU | ANTTRI | ERIRUB | TURVIS | TURPIL | 0,031 | 0,003 | 0,059 |
| 152 | STUVUL | MOTCIN | PHOPHO | LOXCUR | SYLMEL | COCCOC | 0,106 | 0,085 | 0,127 |
| 153 | MOTCIN | APUAPU | TURPHI | SYLATR | CERFAM | LULARB | 0,203 | 0,181 | 0,225 |
| 154 | LULARB | ERIRUB | PRUMOD | SYLCUR | TURMER | CORFRU | 0,127 | 0,090 | 0,163 |
| 155 | TURPIL | DRYMAR | UPUEPO | ORIORI | PHYLUS | CERFAM | 0,093 | 0,072 | 0,114 |
| 156 | STRTUR | TROTRO | ORIORI | CERFAM | COLPAL | CORFRU | 0,202 | 0,179 | 0,224 |
| 157 | PICPIC | CORMON | CARCHL | REGREG | CORFRU | TURPIL | 0,027 | 0,006 | 0,049 |
| 158 | LOXCUR | PHYLUS | PRUMOD | STRTUR | COCCOC | PHOOCH | 0,102 | 0,082 | 0,122 |
| 159 | SYLBOR | LOXCUR | TURPHI | CERFAM | TURVIS | CUCCAN | 0,210 | 0,189 | 0,231 |
| 160 | ERIRUB | DRYMAR | TROTRO | PICVIR | AEGCAU | STUVUL | 0,170 | 0,147 | 0,192 |
| 161 | DENMAJ | PHYCOL | DENMIN | CERFAM | SYLBOR | SYLCAN | 0,138 | 0,116 | 0,161 |
| 162 | TURVIS | REGIGN | CORFRU | ERIRUB | FRICOE | GARGLA | 0,085 | 0,056 | 0,113 |
| 163 | TURPIL | CARCHL | ORIORI | CERBRA | PHYBON | PICPIC | 0,115 | 0,095 | 0,135 |
| 164 | SYLATR | TROTRO | MOTCIN | DRYMAR | APUAPU | STUVUL | 0,153 | 0,131 | 0,176 |
| 165 | SYLCAN | STUVUL | DENMED | CORFRU | STRTUR | GARGLA | 0,111 | 0,083 | 0,140 |
| 166 | TURMER | PHOOCH | PICVIR | HIPPOL | GARGLA | SYLBOR | 0,254 | 0,232 | 0,276 |
| 167 | PHYLUS | APUAPU | MUSSTR | FRICOE | CORONE | LULARB | 0,151 | 0,131 | 0,171 |
| 168 | FRICOE | COLOEN | STRTUR | JYNTOR | SYLATR | TURVIS | 0,162 | 0,140 | 0,184 |
| 169 | TURPHI | REGIGN | PHYLUS | GARGLA | LUSMEG | DENMAJ | 0,218 | 0,196 | 0,241 |
| 170 | PYRPYR | PHYBON | STRTUR | REGREG | DENMED | PASMON | 0,096 | 0,076 | 0,117 |
| 171 | SYLBOR | PICVIR | PASMON | TURVIS | SYLMEL | PYRPYR | 0,094 | 0,074 | 0,114 |
| 172 | STUVUL | PYRPYR | SYLBOR | FRICOE | SYLCAN | REGREG | 0,159 | 0,137 | 0,182 |
| 173 | TURVIS | SYLMEL | SYLCAN | UPUEPO | MUSSTR | MOTCIN | 0,058 | 0,038 | 0,078 |
| 174 | COLOEN | SITEUR | DENMAJ | PICVIR | PYRPYR | ANTTRI | 0,151 | 0,129 | 0,174 |
| 175 | DENMAJ | PYRPYR | CUCCAN | LUSMEG | PICVIR | PHYSIB | 0,178 | 0,157 | 0,198 |
| 176 | LOXCUR | ANTTRI | COLOEN | CORONE | STUVUL | ORIORI | 0,154 | 0,134 | 0,173 |
| 177 | MUSSTR | LULARB | SYLMEL | TURPHI | CORFRU | TROTRO | 0,050 | 0,020 | 0,080 |
| 178 | JYNTOR | PICVIR | PASMON | TURMER | DRYMAR | LUSMEG | 0,226 | 0,204 | 0,249 |
| 179 | CERBRA | COLOEN | DRYMAR | CORMON | ERIRUB | TURPIL | 0,079 | 0,057 | 0,102 |
| 180 | PRUMOD | PHYBON | HIPPOL | LOXCUR | PHYLUS | PHYCOL | 0,108 | 0,084 | 0,132 |
| 181 | SYLCUR | SYLATR | JYNTOR | SERSER | COLOEN | AEGCAU | 0,151 | 0,124 | 0,177 |
| 182 | ANTTRI | UPUEPO | SYLCUR | HIPPOL | DENMED | PHYCOL | 0,090 | 0,068 | 0,112 |
| 183 | LOXCUR | PYRPYR | REGREG | MUSSTR | APUAPU | PICVIR | 0,079 | 0,059 | 0,099 |
| 184 | REGREG | LANCOL | TURPHI | PHYSIB | PHYLUS | PHOPHO | 0,158 | 0,137 | 0,179 |
| 185 | PRUMOD | MUSSTR | PHYCOL | APUAPU | DENMED | REGIGN | 0,097 | 0,074 | 0,120 |
| 186 | COCCOC | MUSSTR | TURMER | HIPPOL | DENMED | COLOEN | 0,232 | 0,208 | 0,256 |
| 187 | TROTRO | DRYMAR | SITEUR | SYLMEL | CORFRU | JYNTOR | 0,027 | 0,005 | 0,049 |
| 188 | LANCOL | LULARB | DENMAJ | CORMON | PYRPYR | REGIGN | 0,085 | 0,062 | 0,107 |
| 189 | CERBRA | ERIRUB | CUCCAN | CORONE | DENMED | PHYCOL | 0,157 | 0,137 | 0,177 |
| 190 | COCCOC | COLOEN | PHYSIB | PHOOCH | TURPHI | DENMIN | 0,167 | 0,143 | 0,190 |
| 191 | CERFAM | STUVUL | REGIGN | REGREG | HIPPOL | PASMON | 0,108 | 0,087 | 0,129 |
| 192 | LULARB | PHYSIB | DENMED | LUSMEG | REGREG | TROTRO | 0,150 | 0,128 | 0,172 |
| 193 | ANTTRI | PHYBON | JYNTOR | PHOOCH | SYLATR | SERSER | 0,187 | 0,165 | 0,209 |
| 194 | TURPIL | CERBRA | LUSMEG | TURVIS | STRTUR | SYLCUR | 0,110 | 0,090 | 0,130 |
| 195 | ORIORI | PHYSIB | SYLCUR | COLOEN | CORMON | PRUMOD | 0,077 | 0,054 | 0,100 |
| 196 | PHOPHO | MUSSTR | PRUMOD | GARGLA | ORIORI | CARCHL | 0,144 | 0,123 | 0,164 |
| 197 | STUVUL | CERBRA | PHOPHO | PHYCOL | PHYLUS | SYLCUR | 0,126 | 0,104 | 0,147 |
| 198 | TURVIS | ANTTRI | LULARB | DRYMAR | PHYSIB | FRICOE | 0,109 | 0,087 | 0,132 |
| 199 | MUSSTR | LOXCUR | TURPIL | ORIORI | PHYCOL | UPUEPO | 0,129 | 0,109 | 0,150 |
| 200 | DENMIN | COLPAL | PICVIR | LULARB | SERSER | STUVUL | 0,249 | 0,229 | 0,269 |
| 201 | LULARB | UPUEPO | CERBRA | TURPIL | COCCOC | LOXCUR | 0,056 | 0,034 | 0,078 |
| 202 | PHYSIB | REGREG | SERSER | CORONE | DRYMAR | PHYBON | 0,142 | 0,123 | 0,161 |
| 203 | CERBRA | PRUMOD | PHYLUS | DRYMAR | ANTTRI | REGIGN | 0,059 | 0,038 | 0,081 |
| 204 | DENMED | LULARB | GARGLA | TURPHI | SYLCUR | SYLCAN | 0,173 | 0,152 | 0,195 |
| 205 | COCCOC | CORMON | PHYCOL | SYLCAN | PHYLUS | DENMED | 0,051 | 0,030 | 0,072 |
| 206 | TURVIS | MUSSTR | REGIGN | LUSMEG | PICVIR | JYNTOR | 0,100 | 0,081 | 0,120 |
| 207 | CORONE | SYLBOR | TURPIL | COLPAL | TURMER | TURVIS | 0,220 | 0,202 | 0,238 |
| 208 | COCCOC | MOTCIN | SITEUR | SERSER | TURPHI | CARCHL | 0,176 | 0,154 | 0,198 |
| 209 | HIPPOL | SITEUR | ANTTRI | COCCOC | MUSSTR | ORIORI | 0,159 | 0,137 | 0,181 |
| 210 | COLOEN | CORMON | PRUMOD | PYRPYR | CARCHL | PHOOCH | 0,045 | 0,023 | 0,067 |
| 211 | TURPIL | DENMED | PICVIR | SYLCUR | AEGCAU | STUVUL | 0,136 | 0,115 | 0,157 |
| 212 | APUAPU | CUCCAN | SYLMEL | HIPPOL | LUSMEG | SERSER | 0,143 | 0,123 | 0,163 |
| 213 | LANCOL | MOTCIN | PICVIR | TURVIS | AEGCAU | JYNTOR | 0,093 | 0,073 | 0,113 |
| 214 | AEGCAU | ORIORI | APUAPU | CORONE | SYLCAN | PASMON | 0,139 | 0,120 | 0,158 |
| 215 | DENMAJ | PHYSIB | CUCCAN | PRUMOD | PYRPYR | SITEUR | 0,198 | 0,178 | 0,219 |
| 216 | SYLMEL | ORIORI | CUCCAN | PHYBON | TURMER | PHYLUS | 0,276 | 0,254 | 0,299 |
| 217 | STUVUL | UPUEPO | CORMON | COLPAL | DENMIN | CUCCAN | 0,238 | 0,219 | 0,258 |
| 218 | PICVIR | CORMON | LULARB | LANCOL | APUAPU | REGIGN | 0,077 | 0,056 | 0,097 |
| 219 | PRUMOD | STRTUR | PHYBON | DENMIN | LULARB | CERFAM | 0,093 | 0,072 | 0,113 |
| 220 | PRUMOD | LULARB | STUVUL | REGIGN | CERFAM | REGREG | 0,100 | 0,080 | 0,120 |
| 221 | PICVIR | ORIORI | GARGLA | ERIRUB | STRTUR | SYLCUR | 0,209 | 0,187 | 0,230 |
| 222 | LOXCUR | PICVIR | MUSSTR | UPUEPO | AEGCAU | DENMAJ | 0,133 | 0,112 | 0,154 |
| 223 | SYLATR | COCCOC | PRUMOD | PHYLUS | DENMIN | STRTUR | 0,135 | 0,113 | 0,157 |
| 224 | PHYSIB | ORIORI | COLOEN | SYLATR | APUAPU | GARGLA | 0,186 | 0,164 | 0,208 |
| 225 | SYLMEL | PHOOCH | ANTTRI | TROTRO | PHOPHO | DENMAJ | 0,150 | 0,128 | 0,172 |
| 226 | STUVUL | SYLCAN | PRUMOD | DENMAJ | SITEUR | PHYCOL | 0,177 | 0,154 | 0,200 |
| 227 | COCCOC | DENMED | MOTCIN | JYNTOR | HIPPOL | ORIORI | 0,119 | 0,097 | 0,141 |
| 228 | TURPIL | CARCHL | SYLCUR | UPUEPO | REGREG | ORIORI | 0,114 | 0,094 | 0,135 |
| 229 | CUCCAN | AEGCAU | LOXCUR | REGREG | ANTTRI | SITEUR | 0,160 | 0,139 | 0,180 |
| 230 | SYLCUR | PYRPYR | TURPIL | GARGLA | PHOPHO | SYLCAN | 0,127 | 0,106 | 0,147 |
| 231 | PASMON | LOXCUR | JYNTOR | COLOEN | DRYMAR | PHYLUS | 0,057 | 0,034 | 0,080 |
| 232 | **TURPHI** | **FRICOE** | **TURMER** | **DENMAJ** | **CUCCAN** | **PHOOCH** | **0,343** | **0,319** | **0,367** |
| 233 | PHYSIB | SYLCAN | CARCHL | PHYCOL | TROTRO | SERSER | 0,177 | 0,155 | 0,198 |
| 234 | SYLATR | CORONE | TURPHI | STRTUR | TROTRO | ORIORI | 0,186 | 0,166 | 0,207 |
| 235 | SERSER | JYNTOR | TURVIS | DENMIN | PASMON | UPUEPO | 0,062 | 0,042 | 0,082 |
| 236 | LUSMEG | CUCCAN | AEGCAU | PHYLUS | CARCHL | SYLCUR | 0,158 | 0,138 | 0,178 |
| 237 | DENMAJ | APUAPU | DRYMAR | PHOPHO | UPUEPO | PHYSIB | 0,102 | 0,081 | 0,124 |
| 238 | FRICOE | DENMAJ | PHYSIB | SYLCUR | REGREG | STRTUR | 0,163 | 0,142 | 0,185 |
| 239 | CARCHL | COLOEN | SYLBOR | CERBRA | SITEUR | TURMER | 0,246 | 0,222 | 0,269 |
| 240 | PASMON | CORMON | FRICOE | TROTRO | ORIORI | PHYSIB | 0,138 | 0,111 | 0,165 |
| 241 | PICPIC | HIPPOL | PRUMOD | REGIGN | DENMAJ | SYLBOR | 0,133 | 0,112 | 0,153 |
| 242 | CORONE | MUSSTR | PICPIC | CORFRU | SYLCAN | COCCOC | 0,134 | 0,112 | 0,156 |
| 243 | FRICOE | LUSMEG | PRUMOD | TURVIS | DENMAJ | CORONE | 0,161 | 0,140 | 0,181 |
| 244 | UPUEPO | CERBRA | TURPHI | SYLBOR | GARGLA | DRYMAR | 0,173 | 0,151 | 0,194 |
| 245 | SYLATR | DENMED | LOXCUR | LULARB | PHOOCH | LUSMEG | 0,181 | 0,158 | 0,204 |
| 246 | DRYMAR | LULARB | DENMED | CERBRA | COLPAL | PHOOCH | 0,216 | 0,196 | 0,235 |
| 247 | COLPAL | STRTUR | DRYMAR | PYRPYR | MOTCIN | TURMER | 0,250 | 0,229 | 0,271 |
| 248 | SYLCAN | LANCOL | PHYSIB | DENMIN | CORONE | DRYMAR | 0,142 | 0,123 | 0,161 |
| 249 | COLOEN | MUSSTR | CARCHL | SYLATR | LOXCUR | FRICOE | 0,222 | 0,195 | 0,249 |
| 250 | DENMAJ | PYRPYR | SYLBOR | JYNTOR | CERBRA | TURPIL | 0,123 | 0,101 | 0,145 |
| 251 | COCCOC | APUAPU | DENMIN | SYLCAN | UPUEPO | DRYMAR | 0,055 | 0,034 | 0,076 |
| 252 | MUSSTR | SITEUR | SYLBOR | CARCHL | CERBRA | TURVIS | 0,088 | 0,068 | 0,109 |
| 253 | SYLATR | PHYLUS | PICVIR | SITEUR | DRYMAR | PHOPHO | 0,135 | 0,114 | 0,157 |
| 254 | REGREG | JYNTOR | SITEUR | DENMAJ | PICPIC | DENMIN | 0,132 | 0,112 | 0,153 |
| 255 | TURVIS | DENMIN | PHYCOL | CORMON | FRICOE | PHYSIB | 0,097 | 0,074 | 0,120 |
| 256 | SYLCAN | PICPIC | PHOOCH | PRUMOD | PHYLUS | PHYSIB | 0,060 | 0,040 | 0,080 |
| 257 | SYLATR | DENMAJ | COLPAL | CARCHL | LANCOL | LULARB | 0,238 | 0,217 | 0,259 |
| 258 | HIPPOL | PICPIC | PHYSIB | TURPIL | PICVIR | PHYCOL | 0,106 | 0,086 | 0,126 |
| 259 | SYLBOR | STRTUR | TURMER | ANTTRI | COLPAL | STUVUL | 0,261 | 0,239 | 0,283 |
| 260 | SYLCAN | PHYSIB | UPUEPO | LUSMEG | REGIGN | AEGCAU | 0,091 | 0,071 | 0,112 |
| 261 | TURVIS | CORONE | REGREG | STRTUR | ORIORI | PICVIR | 0,168 | 0,148 | 0,187 |
| 262 | PICPIC | PICVIR | CORONE | SYLCAN | LOXCUR | HIPPOL | 0,147 | 0,128 | 0,167 |
| 263 | DENMED | TROTRO | PHYLUS | JYNTOR | ERIRUB | CORFRU | 0,025 | 0,003 | 0,047 |
| 264 | GARGLA | COCCOC | MUSSTR | MOTCIN | CORFRU | LULARB | 0,055 | 0,031 | 0,079 |
| 265 | PYRPYR | SYLCAN | AEGCAU | PHYBON | SYLMEL | UPUEPO | 0,056 | 0,036 | 0,075 |
| 266 | SERSER | SYLBOR | GARGLA | PICPIC | SYLMEL | TURMER | 0,234 | 0,211 | 0,256 |
| 267 | REGIGN | AEGCAU | FRICOE | PHYSIB | SITEUR | ORIORI | 0,208 | 0,185 | 0,230 |
| 268 | TURPIL | SYLATR | PICVIR | DENMAJ | SYLCAN | PHYLUS | 0,159 | 0,137 | 0,180 |
| 269 | PASMON | HIPPOL | CORONE | PHOOCH | CORFRU | PHYSIB | 0,134 | 0,112 | 0,156 |
| 270 | DENMIN | FRICOE | AEGCAU | PHYBON | ERIRUB | ANTTRI | 0,205 | 0,182 | 0,228 |
| 271 | CORONE | MOTCIN | ANTTRI | CERBRA | DENMIN | STRTUR | 0,149 | 0,130 | 0,168 |
| 272 | PHOPHO | TURVIS | LOXCUR | PHYBON | TROTRO | ORIORI | 0,124 | 0,104 | 0,145 |
| 273 | GARGLA | ORIORI | TROTRO | JYNTOR | SYLBOR | CORFRU | 0,073 | 0,047 | 0,099 |
| 274 | DRYMAR | DENMIN | PHYLUS | LANCOL | SERSER | HIPPOL | 0,046 | 0,025 | 0,067 |
| 275 | SERSER | LULARB | CORMON | DENMIN | PHYBON | AEGCAU | 0,036 | 0,016 | 0,056 |
| 276 | ERIRUB | SITEUR | CUCCAN | DENMIN | SYLCUR | PHOPHO | 0,187 | 0,166 | 0,208 |
| 277 | MUSSTR | TROTRO | CERBRA | COLPAL | PRUMOD | CORMON | 0,211 | 0,191 | 0,230 |
| 278 | ORIORI | AEGCAU | CORFRU | PHYLUS | PHOPHO | SITEUR | 0,028 | 0,006 | 0,050 |
| 279 | LANCOL | STUVUL | PHOOCH | TURPHI | MOTCIN | PHYSIB | 0,177 | 0,156 | 0,198 |
| 280 | CERFAM | LOXCUR | CERBRA | ORIORI | ANTTRI | DENMAJ | 0,166 | 0,144 | 0,188 |
| 281 | PHOOCH | CORMON | AEGCAU | PHYBON | FRICOE | ANTTRI | 0,083 | 0,059 | 0,108 |
| 282 | SYLBOR | TURMER | MUSSTR | CORONE | ANTTRI | TURPHI | 0,203 | 0,181 | 0,224 |
| 283 | PRUMOD | CUCCAN | PHYCOL | DENMED | CERBRA | STUVUL | 0,183 | 0,162 | 0,204 |
| 284 | LANCOL | TURPHI | CERBRA | DENMED | PHYCOL | ORIORI | 0,201 | 0,180 | 0,222 |
| 285 | TROTRO | AEGCAU | SITEUR | CUCCAN | MUSSTR | ANTTRI | 0,187 | 0,166 | 0,208 |
| 286 | PICPIC | LULARB | ANTTRI | PHYLUS | DRYMAR | SYLBOR | 0,084 | 0,063 | 0,104 |
| 287 | FRICOE | LUSMEG | TURMER | MUSSTR | CORMON | JYNTOR | 0,237 | 0,211 | 0,262 |
| 288 | CUCCAN | FRICOE | SITEUR | PHYBON | TURVIS | SYLCUR | 0,188 | 0,166 | 0,210 |
| 289 | COLOEN | LUSMEG | UPUEPO | SYLBOR | STRTUR | PASMON | 0,110 | 0,089 | 0,131 |
| 290 | STUVUL | REGREG | CUCCAN | ERIRUB | CARCHL | HIPPOL | 0,197 | 0,175 | 0,218 |
| 291 | SYLATR | STRTUR | PICPIC | LULARB | COLPAL | GARGLA | 0,252 | 0,231 | 0,272 |
| 292 | PYRPYR | CORFRU | PHYSIB | PICPIC | PHYCOL | CORMON | 0,029 | 0,007 | 0,051 |
| 293 | SYLMEL | GARGLA | LUSMEG | ANTTRI | AEGCAU | FRICOE | 0,161 | 0,140 | 0,182 |

*Notes*: Parameter estimates are in log-scale and the control group with the highest parameter estimate (i.e. with the best predictive power for forest bird density) is marked in **bold** (Model 232; see a key for species codes in Table S1.1).

**
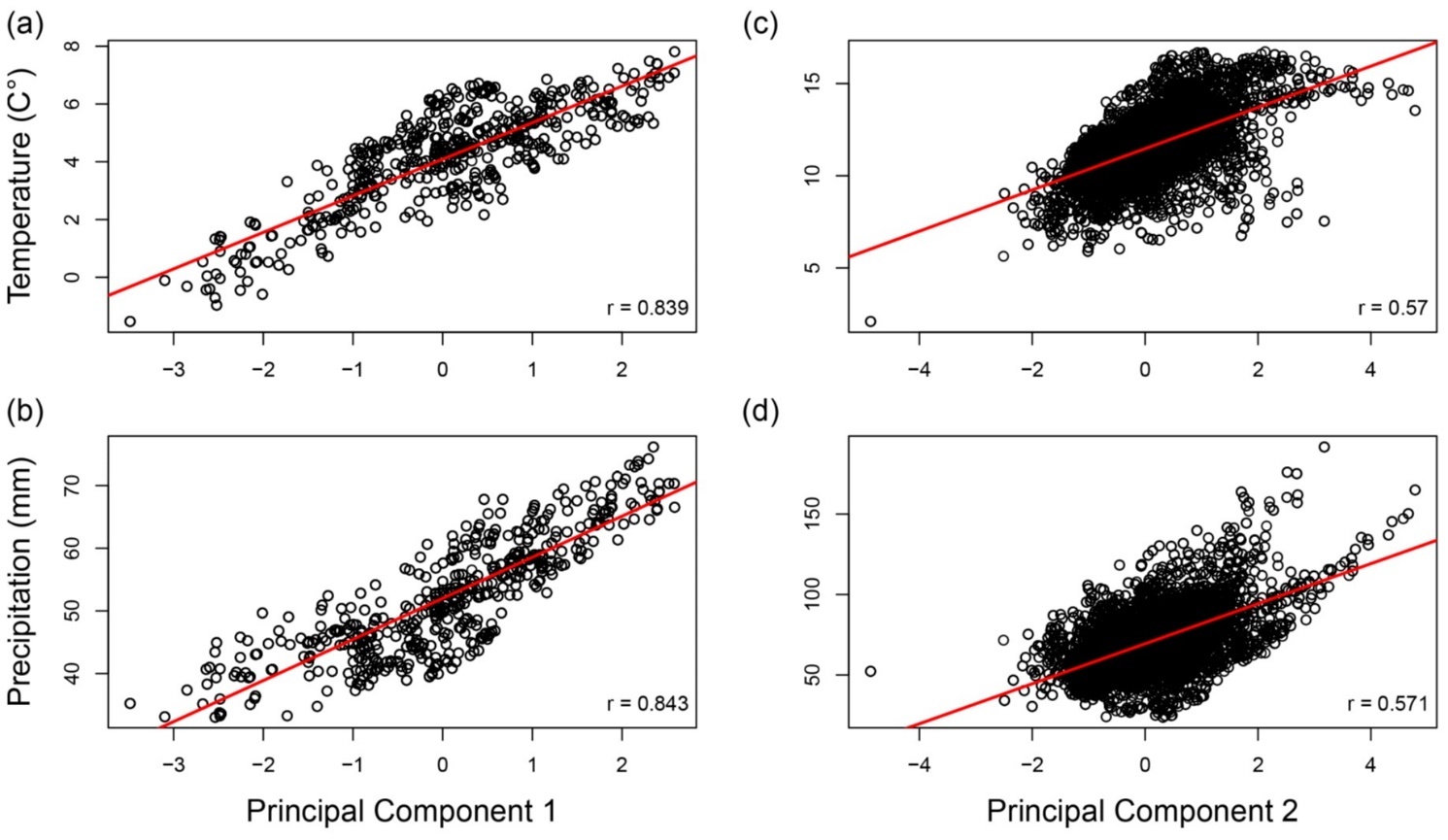
**

**Figure S1.1.** Scatterplots displaying the relationship between climate variables and the first principal component (PC1) in Finland (a-b) and the second principal component (PC2) in France (c-d). In Finland, there was a strong positive correlation between PC1 and both temperature (a) and precipitation (b). In France, a weak positive correlation was observed between PC2 and both temperature (c) and precipitation (b). The points are measured values of mean annual temperature (a and c) and sum of annual precipitation (b and d) at the sampling sites, while the line represents a fitted regression of environmental data on PC1 and PC2. See main text section 2.2. for details of the Principal Component Analysis. Correlation coefficient (Pearson correlation) is shown in the lower right corner of each panel.

**
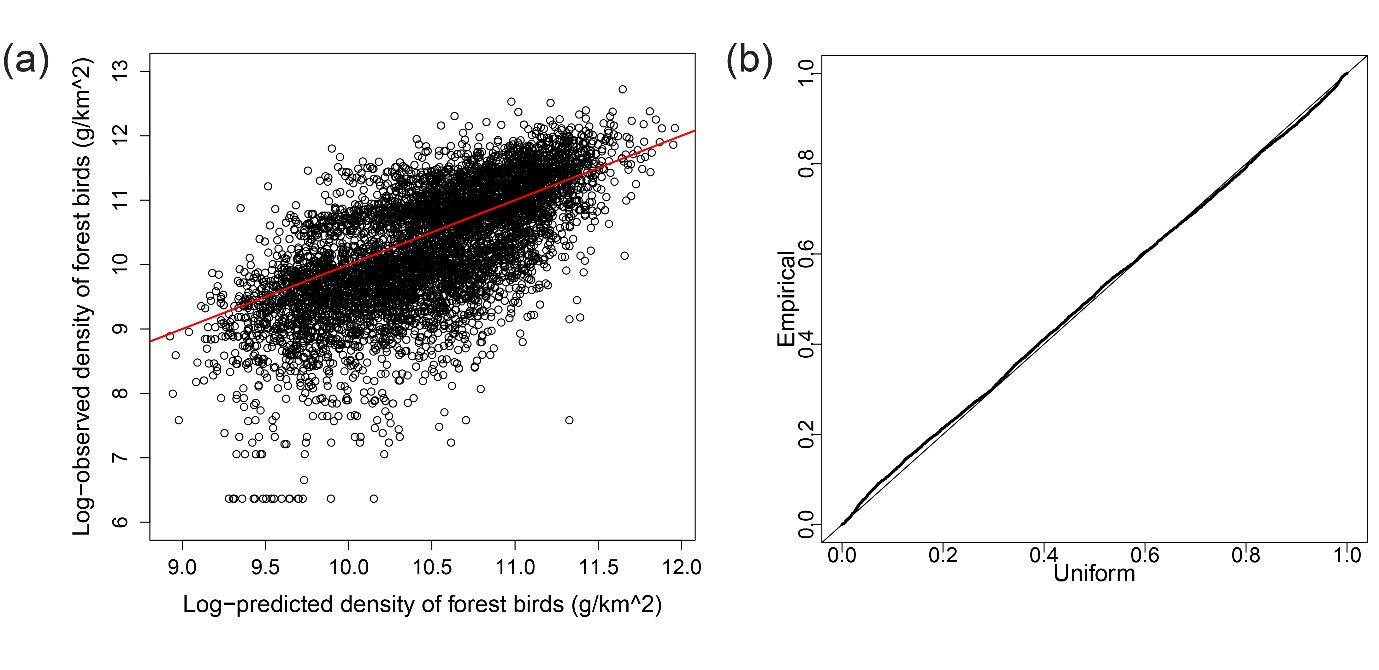
Figure S1.2**. Model diagnostics produced by VAST (Thorson and Barnett 2017, Thorson 2019) to assess the model fit for the forest bird density in Finland. The relationship between observed and predicted log-density of forest birds (g/km^2^) with the line representing relationship, where predicted log-density equals observed log-density of forest birds (x = y) (a); quantile-quantile plot showing the theoretical quantiles (Uniform) and the sample quantiles (Empirical) (b).

**
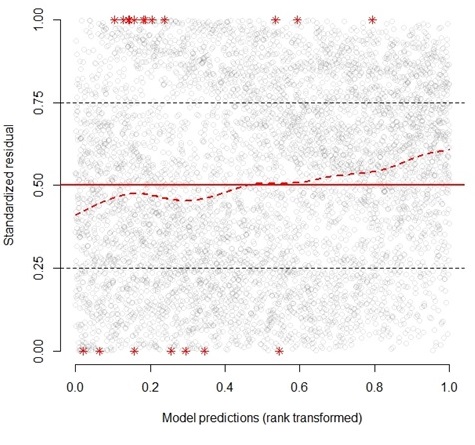
**

**Figure S1.3.** Simulated residuals in relation to model predictions produced by the function “simulateResiduals” from R package “DHARMa” (Hartig 2020) for the linear model for the forest bird density in Finland. The residuals were derived from 1000 simulated data sets from the fitted model. The plot shows the distribution of the standardized residuals against the ranked model predictions, the red stars representing outliers, 0.25 and 0.75 empirical quantiles are displayed by black dashed lines, 0.5 quantile with red solid line and the red dashed line is a smoothing spline.

**
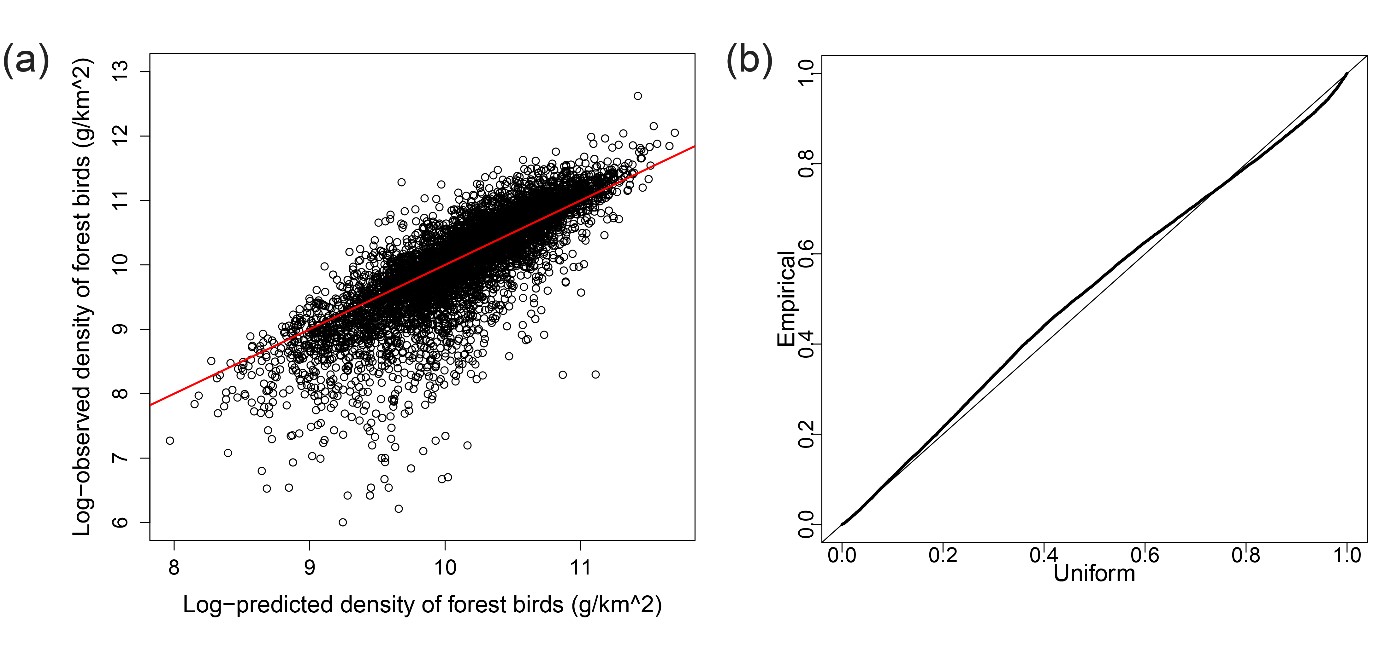
Figure S1.4.** Model diagnostics produced by VAST (Thorson and Barnett 2017, Thorson 2019) to assess the model fit for the forest bird density in France. The relationship between observed and predicted log-density of forest birds (g/km^2^) with the line representing relationship, where predicted log-density equals observed log-density of forest birds (x = y) (a); quantile-quantile plot showing the theoretical quantiles (Uniform) and the sample quantiles (Empirical) (b).

**
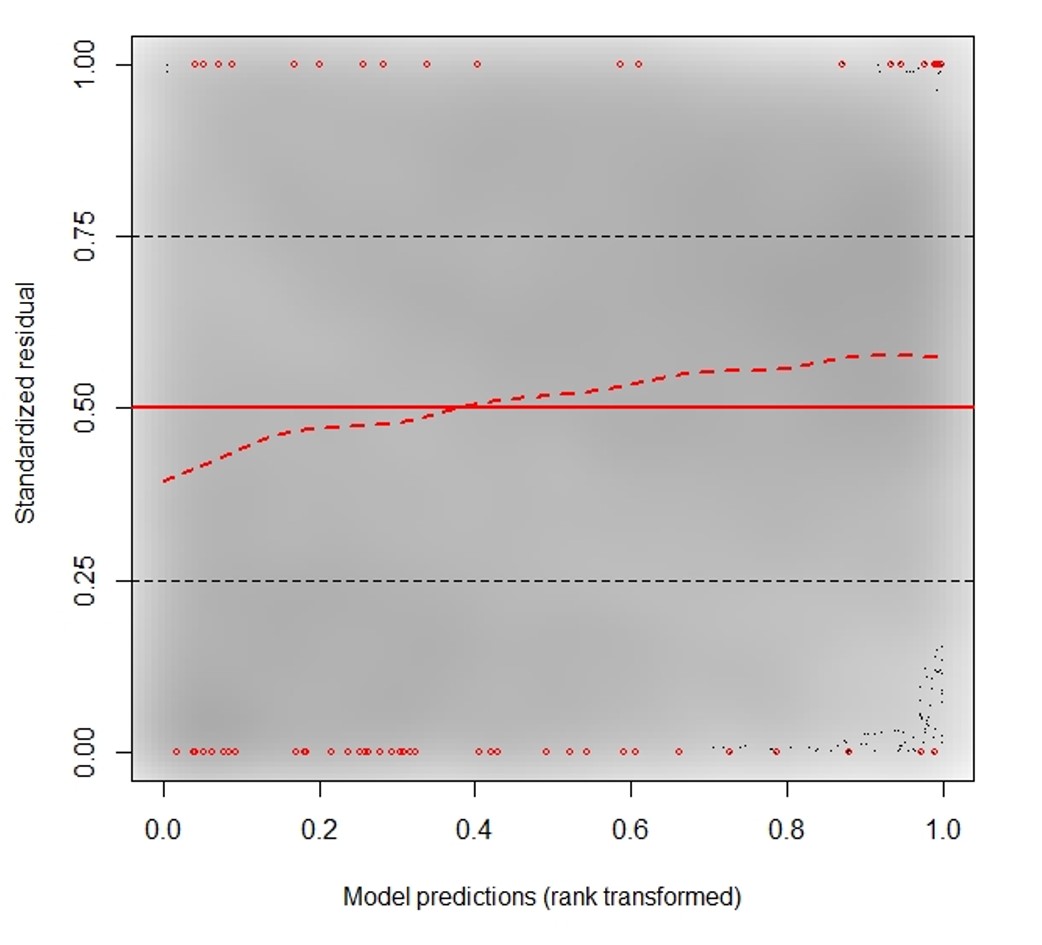
**

**Figure S1.5.** Simulated residuals in relation to model predictions produced by the function “simulateResiduals” from R package “DHARMa” (Hartig 2020) for the non-linear model for the forest bird density in France. The residuals were derived from 1000 simulated data sets from the fitted model. The plot shows the distribution of the standardized residuals against the ranked model predictions, the red circles representing outliers, 0.25 and 0.75 empirical quantiles are displayed by black dashed lines, 0.5 quantile with red solid line and the red dashed line is a smoothing spline.

**
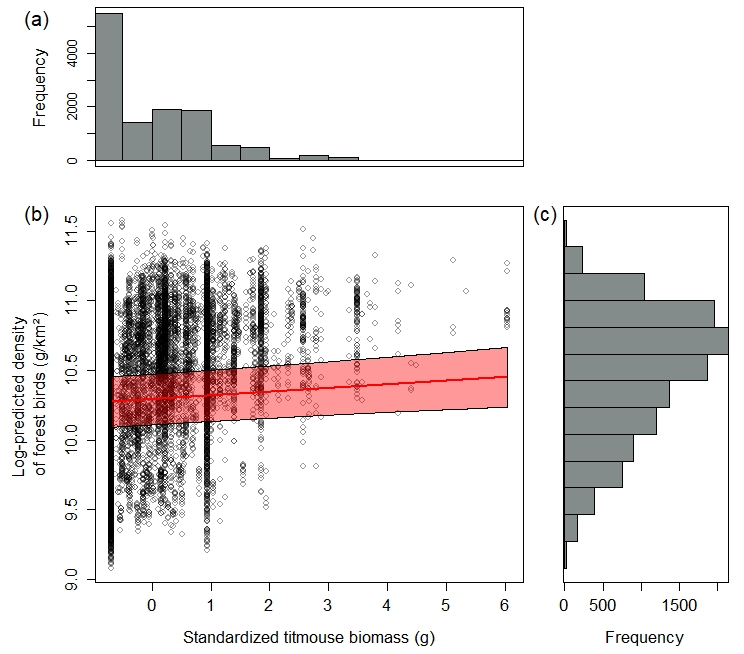
**

**Figure S1.6.** Model results for analysis of the Finnish data, where 2.5% of the lowest and the highest forest bird abundance data points have been removed. Frequency distribution of standardized titmouse abundance (given as biomass; g) in Finland (a). The relationship between log-predicted density of forest birds (g/km^2^) and standardized titmouse abundance (given as biomass; g) in Finland in 2001 (i.e. first study year; *β* = 10.293, $\gamma_{1}$ = 0.026; see Table 1 in the main text for definition of all symbols) (b). Circles are predicted forest bird densities for the sampling points and the fitted line with 95% confidence intervals derives from the spatial Gompertz model (see Methods section 2.3. in the main text for details) visualizing the linear relationship between predicted forest bird density and titmouse abundance. There was minor variance among years in the intercept (10.194 < *β* < 10.360), so the elevation of the line varies among years, but the slope remains the same. Frequency distribution of log-predicted density of forest birds (g/km^2^) in Finland (c).

**
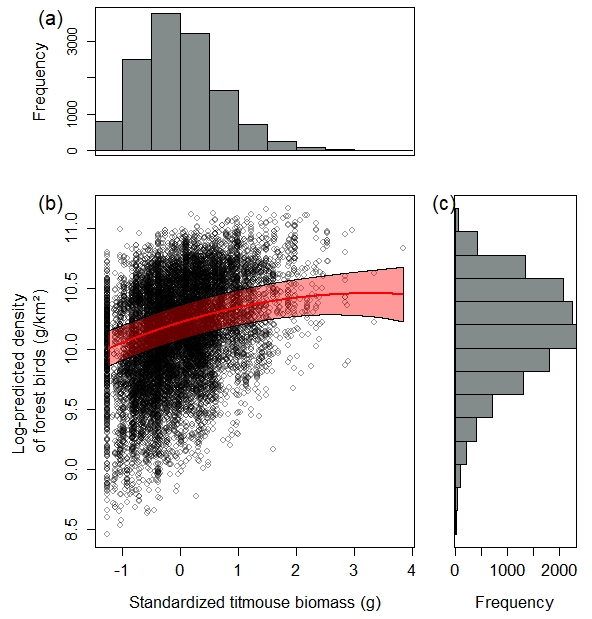
**

**Figure S1.7.** Model results for analysis of the French data, where 2.5% of the lowest and the highest forest bird abundance data points have been removed. Frequency distribution of standardized titmouse abundance (given as biomass; g) in France (a). The relationship between log-predicted density of forest birds (g/km^2^) and standardized titmouse abundance (given as biomass; g) in France in 2001 (i.e. first study year; *β* = 10.220, $\gamma_{1}$ = 0.148, $\gamma_{2}$ = -0.023; see Table 1 in the main text for definition of all symbols) (b). Circles are predicted forest bird densities for the sampling points and the fitted line with 95% confidence intervals derives from the spatial Gompertz model (see Methods section 2.3. in the main text for details) visualizing the linear relationship between predicted forest bird density and titmouse abundance. There was minor variance among years in the intercept (10.148 < *β* < 10.241), so the elevation of the line varies among years, but the slope remains the same. Frequency distribution of log-predicted density of forest birds (g/km^2^) in France (c).

**References**

Hartig, F. 2020. DHARMa: Residual Diagnostics for Hierarchical (Multi-Level / Mixed) Regression Models. R package version 0.3.0. <https://CRAN.R-project.org/package=DHARMa>

Thorson, J.T. 2019. Guidance for decisions using the Vector Autoregressive Spatio-Temporal (VAST) package in stock, ecosystem, habitat and climate assessments. Fisheries Research 210:143–161.

Thorson, J.T., and Barnett, L.A.K. 2017. Comparing estimates of abundance trends and distribution shifts using single- and multispecies models of fishes and biogenic habitat. ICES Journal of Marine Science 74:1311–1321.
