# Supplementary material for: Titmice are a better indicator of bird density in Northern European than in Western European forests
Source: Ecol Evol. 2022 Feb 12;12(2):e8479. doi: 10.1002/ece3.8479 (PMC8840900; doi:10.1002/ece3.8479)
Supplement: Supplementary file 2 — Supplementary Material [file ECE3-12-e8479-s001.docx]

**Supporting Information 2.**

Kajanus, M.H., Forsman, J.T., Vollstädt, M.G.R., Devictor, V., Elo, M., Lehikoinen, A., Mönkkönen, M., Thorson, J.T., and Kivelä, S.M. 2021. Titmice are a better indicator of bird density in Northern European than in Western European forests. *Ecology and Evolution*.

**Habitat class-specific analysis**

To avoid bias from the heterogeneity of different forest habitat types, we repeated the analysis described in the main text to habitat-specific data sets. We estimated habitat-specific Shannon-Wiener diversity indices (H’; Shannon and Weaver 1949) for each habitat that was classified in the data. We then compared the diversity indices among habitats using pairwise Wilcoxon rank sum test with Bonferroni correction in R (2019) statistical environment version 3.6.2. We first separated habitats in Finland into deciduous, spruce and pine forests as the main classes due to their distinct structural differences. We then combined the remaining habitats with one of these main classes according to a similar (*p* > 0.05) Shannon entropy estimate (see Table S2.1 and Fig. S2.1). Consequently, we defined four habitat classes in Finland, deciduous forest including deciduous and mixed forest, spruce forest including spruce and young forest, pine forest including pine forest and pine swamp, and deciduous bush. In France, we considered all the habitat types as individual data sets due to their unique forest structures (Table S2.2 and Fig. S2.2). Thus, in France we had coniferous, mixed, deciduous, and young forest, and coppice. We then repeated the analysis separately for each of the habitat class-specific subsets of the data (see Tables S2.3-S2.8 and Fig. S2.3-S2.7 for results).

**Table S2.1.** *p*-values for pairwise comparisons of Shannon entropy (H’) among all unique forest habitat types in Finland (2001-2013) with a Bonferroni corrected pairwise Wilcoxon rank sum test.

| HABITAT TYPE | deciduous bush | deciduous forest | mixed forest | pine forest | pine swamp | spruce forest |
| --- | --- | --- | --- | --- | --- | --- |
| deciduous forest | 0.021 | - | - | - | - | - |
| mixed forest | <0.001 | 0.233 | - | - | - | - |
| pine forest | 1.000 | <0.001 | <0.001 | - | - | - |
| pine swamp | 1.000 | 0.127 | <0.001 | 1.000 | - | - |
| spruce forest | <0.001 | 1.000 | 1.000 | <0.001 | 0.003 | - |
| young coniferous forest | 0.716 | 1.000 | 1.000 | 0.055 | 0.686 | 1.000 |

**Table S2.2.** *p*-values for pairwise comparisons of Shannon entropy (H’) among all unique forest habitat types in France (2001-2013) with a Bonferroni corrected pairwise Wilcoxon rank sum test.

| HABITAT TYPE | Coniferous woodland | Coppice | Deciduous woodland | Mixed woodland |
| --- | --- | --- | --- | --- |
| Coppice | 0.4613 | - | - | - |
| Deciduous woodland | <0.001 | <0.001 | - | - |
| Mixed woodland | <0.001 | <0.001 | <0.001 | - |
| Young forest | <0.001 | <0.001 | <0.001 | 1.000 |

**Table S2.3.** Parameter estimates and their 95% confidence intervals for both the model with a linear and a quadratic relationship between titmouse abundance (measured as biomass) and forest bird density in deciduous forest in Finland 2001–2013. The linear model fitted the data best (ΔAIC = 1.94 in favor of the linear model). See table 1 for other details.

| PARAMETER | ESTIMATE | LOWER 95% CI | UPPER 95% CI |
| --- | --- | --- | --- |
| Linear model |  |  |  |
| Titmouse abundance (γ_1_) | **0.050** | **0.019** | **0.080** |
| Environmental PC (γ_3_) | 0.013 | -0.085 | 0.112 |
| Standard deviation of spatial variation (*σ_ω_*) | 1.409 | 1.964 | 0.853 |
| Standard deviation of spatio-temporal variation (*σ_ε_*) | 0.257 | 0.431 | 0.083 |
| Quadratic model |  |  |  |
| Titmouse abundance (γ_1_) | **0.046** | **0.005** | **0.088** |
| [Titmouse abundance]^2^ (γ_2_) | 0.002 | -0.018 | 0.023 |
| Environmental PC (γ_3_) | 0.014 | -0.085 | 0.112 |
| Standard deviation of spatial variation (*σ_ω_*) | 1.408 | 1.964 | 0.853 |
| Standard deviation of spatio-temporal variation (*σ_ε_*) | 0.257 | 0.083 | 0.431 |

*Notes*: Parameter estimates are in log-scale and parameters that are different from zero at 95% confidence level are highlighted in **bold**. Variance components are not highlighted because they are inevitably non-negative.

**Table S2.4.** Parameter estimates and their 95% confidence intervals for both the model with a linear and a quadratic relationship between titmouse abundance (measured as biomass) and forest bird density in spruce forest in Finland 2001–2013. The linear model fitted the data best (ΔAIC = 1.11 in favor of the linear model). See table 1 for other details.

| PARAMETER | ESTIMATE | LOWER 95% CI | UPPER 95% CI |
| --- | --- | --- | --- |
| Linear model |  |  |  |
| Titmouse abundance (γ_1_) | 0.005 | -0.033 | 0.044 |
| Environmental PC (γ_3_) | 0.040 | -0.086 | 0.166 |
| Standard deviation of spatial variation (*σ_ω_*) | 1.225 | 1.656 | 0.795 |
| Standard deviation of spatio-temporal variation (*σ_ε_*) | 0.324 | 0.102 | 0.547 |
| Quadratic model |  |  |  |
| Titmouse abundance (γ_1_) | 0.025 | -0.031 | 0.082 |
| [Titmouse abundance]^2^ (γ_2_) | -0.013 | -0.041 | 0.014 |
| Environmental PC (γ_3_) | 0.039 | -0.087 | 0.165 |
| Standard deviation of spatial variation (*σ_ω_*) | 1.228 | 0.786 | 1.670 |
| Standard deviation of spatio-temporal variation (*σ_ε_*) | 0.320 | 0.093 | 0.547 |

*Notes*: Parameter estimates are in log-scale.

**Table S2.5.** Parameter estimates and their 95% confidence intervals for both the model with a linear and a quadratic relationship between titmouse abundance (measured as biomass) and forest bird density in deciduous forest in France 2001–2013. The quadratic model fitted the data best (ΔAIC = 2.5 in favor of the quadratic model). See table 1 for other details.

| PARAMETER | ESTIMATE | LOWER 95% CI | UPPER 95% CI |
| --- | --- | --- | --- |
| Linear model |  |  |  |
| Titmouse abundance (γ_1_) | **0.118** | **0.097** | **0.138** |
| Environmental PC (γ_3_) | -0.009 | -0.041 | 0.023 |
| Standard deviation of spatial variation (*σ_ω_*) | 0.594 | 0.510 | 0.679 |
| Standard deviation of spatio-temporal variation (*σ_ε_*) | -0.242 | -0.270 | -0.213 |
| Quadratic model |  |  |  |
| Titmouse abundance (γ_1_) | **0.128** | **0.106** | **0.150** |
| [Titmouse abundance]^2^ (γ_2_) | **-0.016** | **-0.031** | **-0.001** |
| Environmental PC (γ_3_) | -0.008 | -0.040 | 0.024 |
| Standard deviation of spatial variation (σ_ω_) | 0.594 | 0.679 | 0.510 |
| Standard deviation of spatio-temporal variation (σ_ε_) | 0.242 | 0.270 | 0.213 |

*Notes*: Parameter estimates are in log-scale and parameters that are different from zero at 95% confidence level are highlighted in **bold**. Variance components are not highlighted because they are inevitably non-negative.

**Table S2.6.** Parameter estimates and their 95% confidence intervals for both the model with a linear and a quadratic relationship between titmouse abundance (measured as biomass) and forest bird density in coniferous forest in France 2001–2013. The linear model fitted the data best (ΔAIC = 2.0 in favor of the linear model). See table 1 for other details.

| PARAMETER | ESTIMATE | LOWER 95% CI | UPPER 95% CI |
| --- | --- | --- | --- |
| Linear model |  |  |  |
| Titmouse abundance (γ_1_) | **0.086** | **0.052** | **0.119** |
| Environmental PC (γ_3_) | -0.011 | -0.084 | 0.063 |
| Standard deviation of spatial variation (*σ_ω_*) | 0.962 | 0.722 | 1.202 |
| Standard deviation of spatio-temporal variation (*σ_ε_*) | 0.447 | 0.336 | 0.557 |
| Quadratic model |  |  |  |
| Titmouse abundance (γ_1_) | **0.086** | **0.046** | **0.126** |
| [Titmouse abundance]^2^ (γ_2_) | -0.001 | -0.022 | 0.021 |
| Environmental PC (γ_3_) | -0.011 | -0.084 | 0.063 |
| Standard deviation of spatial variation (σ_ω_) | 0.962 | 0.722 | 1.202 |
| Standard deviation of spatio-temporal variation (σ_ε_) | 0.447 | 0.336 | 0.557 |

*Notes*: Parameter estimates are in log-scale and parameters that are different from zero at 95% confidence level are highlighted in **bold**. Variance components are not highlighted because they are inevitably non-negative.

**Table S2.7.** Parameter estimates and their 95% confidence intervals for both the model with a linear and a quadratic relationship between titmouse abundance (measured as biomass) and forest bird density in mixed forest in France 2001–2013. The linear model fitted the data best (ΔAIC = 0.92 in favor of the linear model). See table 1 for other details.

| PARAMETER | ESTIMATE | LOWER 95% CI | UPPER 95% CI |
| --- | --- | --- | --- |
| Linear model |  |  |  |
| Titmouse abundance (γ_1_) | **0.102** | **0.076** | **0.127** |
| Environmental PC (γ_3_) | -0.028 | -0.078 | 0.022 |
| Standard deviation of spatial variation (*σ_ω_*) | 0.763 | 0.621 | 0.905 |
| Standard deviation of spatio-temporal variation (*σ_ε_*) | 0.212 | 0.130 | 0.294 |
| Quadratic model |  |  |  |
| Titmouse abundance (γ_1_) | **0.109** | **0.080** | **0.138** |
| [Titmouse abundance]^2^ (γ_2_) | -0.009 | -0.026 | 0.008 |
| Environmental PC (γ_3_) | -0.028 | -0.078 | 0.023 |
| Standard deviation of spatial variation (σ_ω_) | 0.762 | 0.903 | 0.621 |
| Standard deviation of spatio-temporal variation (σ_ε_) | 0.212 | 0.129 | 0.294 |

*Notes*: Parameter estimates are in log-scale and parameters that are different from zero at 95% confidence level are highlighted in **bold**. Variance components are not highlighted because they are inevitably non-negative.

**Table S2.8.** Parameter estimates and their 95% confidence intervals for both the model with a linear and a quadratic relationship between titmouse abundance (measured as biomass) and forest bird density in young forest in France 2001–2013. The quadratic model fitted the data best (ΔAIC = 3.32 in favor of the quadratic model). See table 1 for other details.

| PARAMETER | ESTIMATE | LOWER 95% CI | UPPER 95% CI |
| --- | --- | --- | --- |
| Linear model |  |  |  |
| Titmouse abundance (γ_1_) | **0.113** | **0.059** | **0.167** |
| Environmental PC (γ_3_) | -0.088 | -0.188 | 0.013 |
| Standard deviation of spatial variation (*σ_ω_*) | 0.864 | 0.679 | 1.049 |
| Standard deviation of spatio-temporal variation (*σ_ε_*) | 0.304 | 0.183 | 0.426 |
| Quadratic model |  |  |  |
| Titmouse abundance (γ_1_) | **0.158** | **0.093** | **0.224** |
| [Titmouse abundance]^2^ (γ_2_) | **-0.041** | **-0.076** | **-0.007** |
| Environmental PC (γ_3_) | -0.089 | -0.189 | 0.011 |
| Standard deviation of spatial variation (σ_ω_) | 0.857 | 1.040 | 0.674 |
| Standard deviation of spatio-temporal variation (σ_ε_) | 0.303 | 0.424 | 0.183 |

*Notes*: Parameter estimates are in log-scale and parameters that are different from zero at 95% confidence level are highlighted in **bold**. Variance components are not highlighted because they are inevitably non-negative.

**
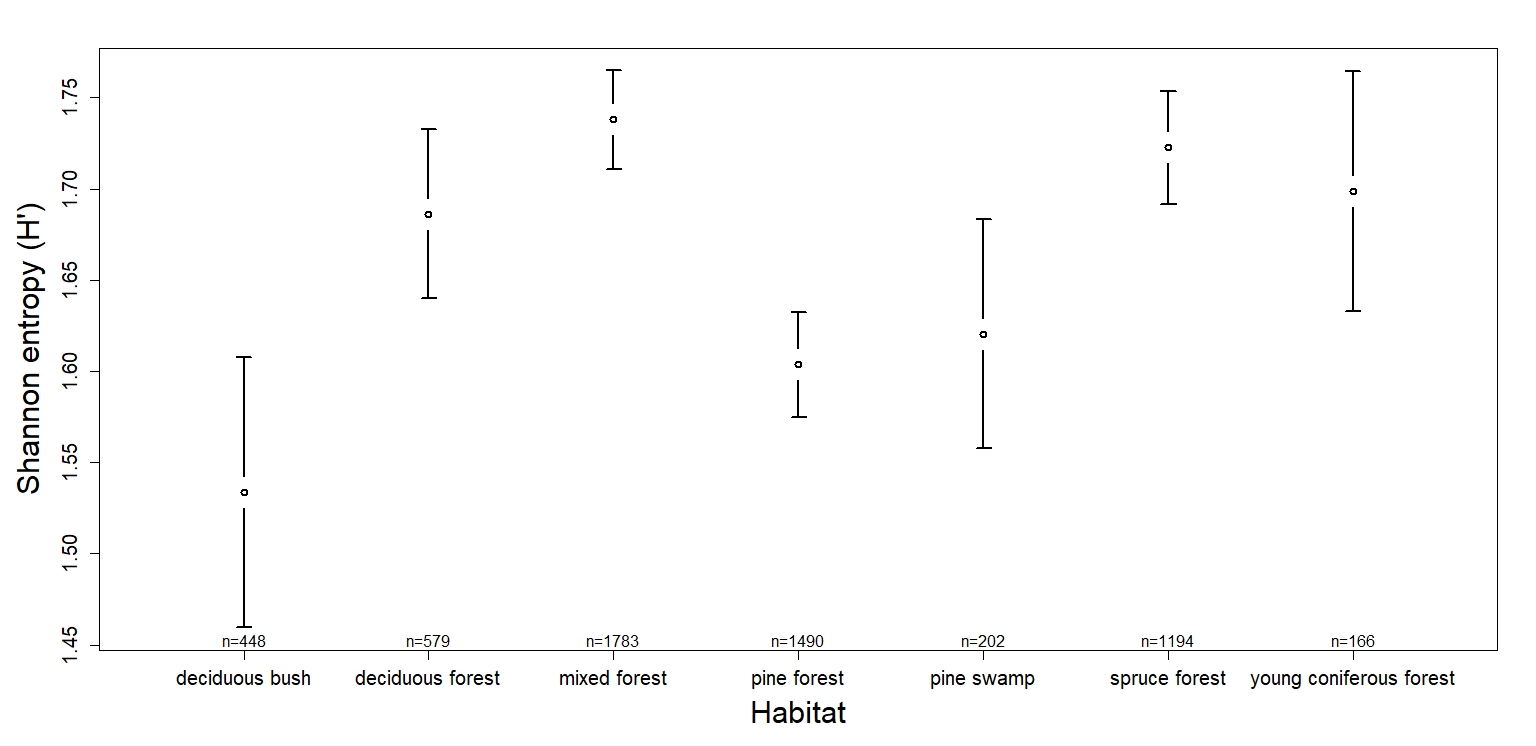
Figure S2.1.** Mean of Shannon entropy (H’) and Bonferroni corrected 95% confidence intervals (i.e. 99.29% CIs) for each forest habitat type in Finland (2001-2013). Sample size (*n*; the number of point counts) is given for each habitat over the entire study period.


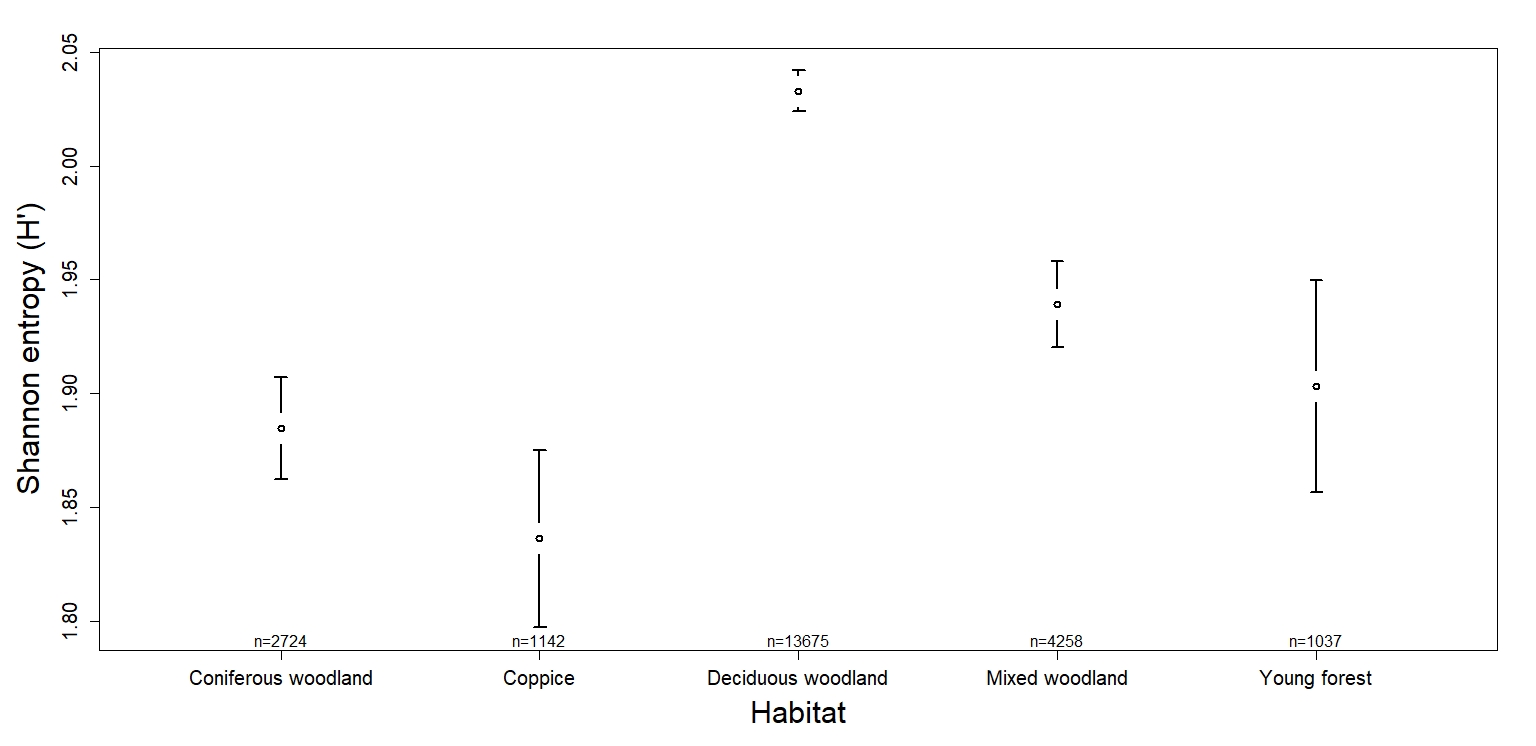
**Figure S2.2.** Mean of Shannon entropy (H’) and Bonferroni corrected 95% confidence intervals (i.e. 99% CIs) for each forest habitat type in France (2001-2013). Sample size (*n*; the number of point counts) is given for each habitat over the entire study period.

**
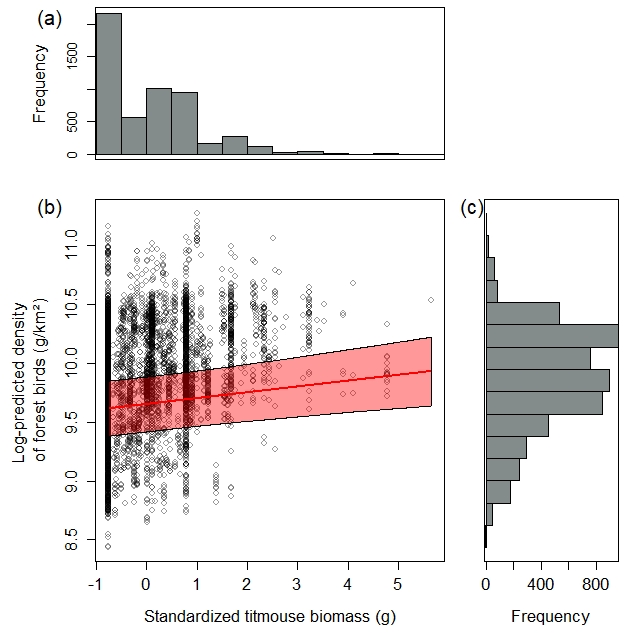
**

**Figure S2.3.** Frequency distribution of standardized titmouse abundance (given as biomass; g) in deciduous forest in Finland (a). The relationship between log-predicted density of forest birds (g/km^2^) and standardized titmouse abundance (given as biomass; g) in deciduous forest in Finland in 2001 (i.e. first study year; *β* = 9.652, $\gamma_{1}$ = 0.050; see Table 1 in the main text for definition of all symbols) (b). Circles are predicted forest bird densities for the sampling points and the fitted line with 95% confidence intervals derives from the spatial Gompertz model (see Methods section 2.3. in the main text for details) visualizing the quadratic relationship between predicted forest bird density and titmouse abundance. There was minor variance among years in the intercept (9.602 < *β* < 9.850), so the elevation of the line varies among years, but the slope remains the same. Frequency distribution of log-predicted density of forest birds (g/km^2^) in deciduous forest in Finland (c).

**
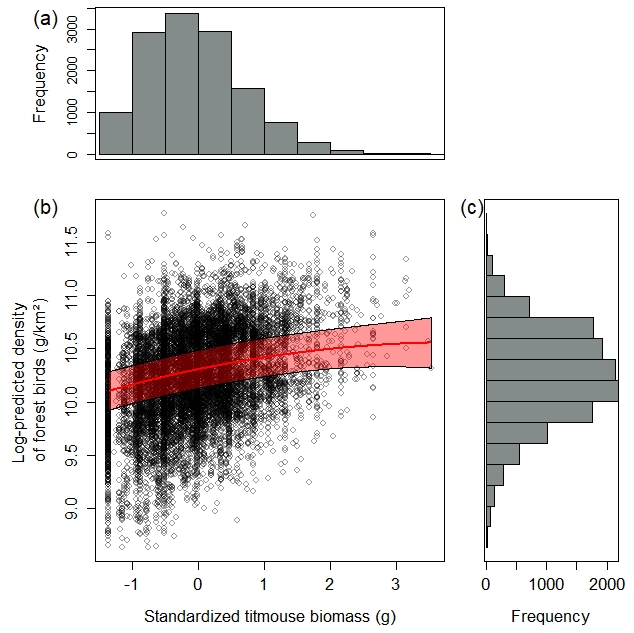
**

**Figure S2.4.** Frequency distribution of standardized titmouse abundance (given as biomass; g) in deciduous forest in France (a). The relationship between log-predicted density of forest birds (g/km^2^) and standardized titmouse abundance (given as biomass; g) in deciduous forest in France in 2001 (i.e. first study year; *β* = 10.306, $\gamma_{1}$ = 0.128, $\gamma_{2}$ = -0.016; see Table 1 in the main text for definition of all symbols) (b). Circles are predicted forest bird densities for the sampling points and the fitted line with 95% confidence intervals derives from the spatial Gompertz model (see Methods section 2.3. in the main text for details) visualizing the quadratic relationship between predicted forest bird density and titmouse abundance. There was minor variance among years in the intercept (10.150 < *β* < 10.306), so the elevation of the line varies among years, but the curve remains the same. Frequency distribution of log-predicted density of forest birds (g/km^2^) in deciduous forest in France (c).

**
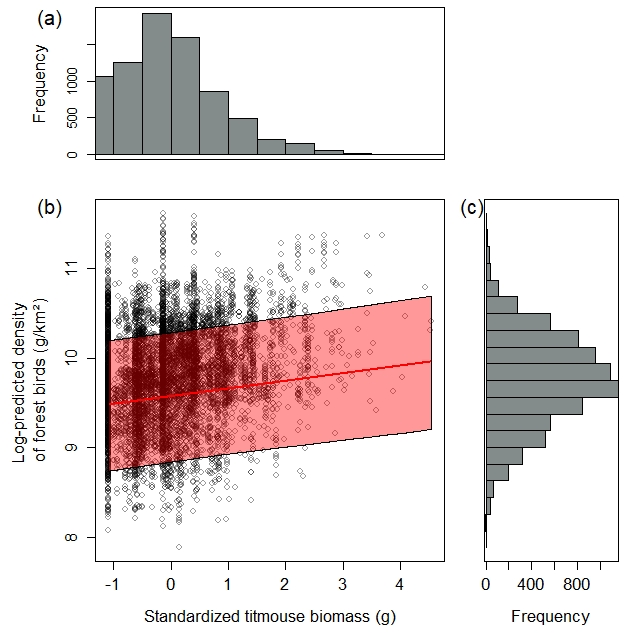
**

**Figure S2.5.** Frequency distribution of standardized titmouse abundance (given as biomass; g) in coniferous forest in France (a). The relationship between log-predicted density of forest birds (g/km^2^) and standardized titmouse abundance (given as biomass; g) in coniferous forest in France in 2001 (i.e. first study year; *β* = 9.573, $\gamma_{1}$ = 0.086; see Table 1 in the main text for definition of all symbols) (b). Circles are predicted forest bird densities for the sampling points and the fitted line with 95% confidence intervals derives from the spatial Gompertz model (see Methods section 2.3. in the main text for details) visualizing the quadratic relationship between predicted forest bird density and titmouse abundance. There was minor variance among years in the intercept (9.573 < *β* < 10.096), so the elevation of the line varies among years, but the slope remains the same. Frequency distribution of log-predicted density of forest birds (g/km^2^) in coniferous forest in France (c).

**
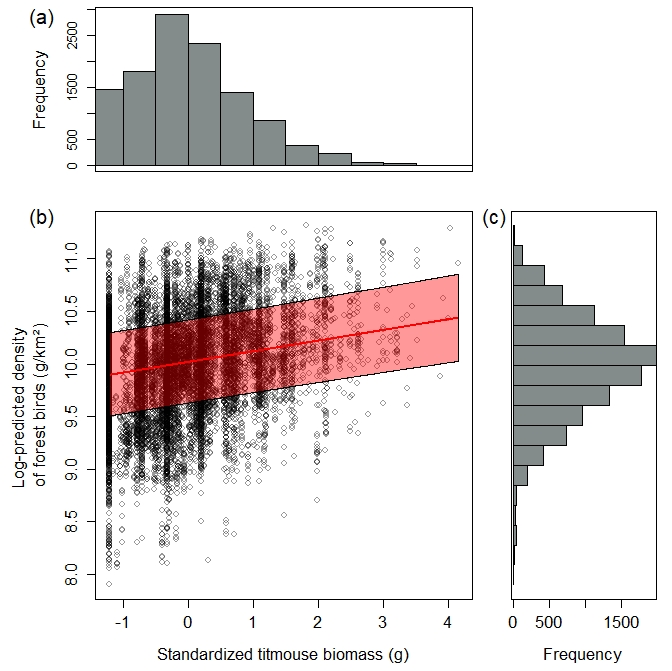
**

**Figure S2.6.** Frequency distribution of standardized titmouse abundance (given as biomass; g) in mixed forest in France (a). The relationship between log-predicted density of forest birds (g/km^2^) and standardized titmouse abundance (given as biomass; g) in mixed forest in France in 2001 (i.e. first study year; *β* = 10.016, $\gamma_{1}$ = 0.102; see Table 1 in the main text for definition of all symbols) (b). Circles are predicted forest bird densities for the sampling points and the fitted line with 95% confidence intervals derives from the spatial Gompertz model (see Methods section 2.3. in the main text for details) visualizing the quadratic relationship between predicted forest bird density and titmouse abundance. There was minor variance among years in the intercept (9.914 < *β* < 10.194), so the elevation of the line varies among years, but the slope remains the same. Frequency distribution of log-predicted density of forest birds (g/km^2^) in mixed forest in France (c).

**
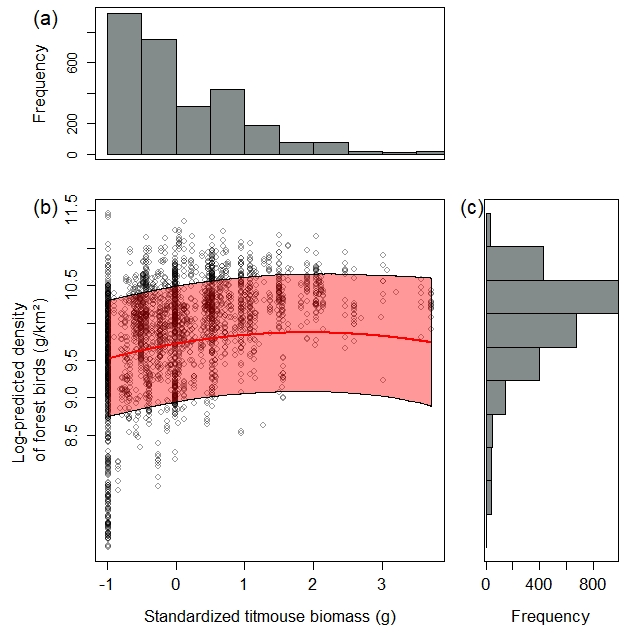
**

**Figure S2.7.** Frequency distribution of standardized titmouse abundance (given as biomass; g) in young forest in France (a). The relationship between log-predicted density of forest birds (g/km^2^) and standardized titmouse abundance (given as biomass; g) in young forest in France in 2001 (i.e. first study year; *β* = 9.720, $\gamma_{1}$ = 0.158, $\gamma_{2}$ = -0.041; see Table 1 in the main text for definition of all symbols) (b). Circles are predicted forest bird densities for the sampling points and the fitted line with 95% confidence intervals derives from the spatial Gompertz model (see Methods section 2.3. in the main text for details) visualizing the quadratic relationship between predicted forest bird density and titmouse abundance. There was minor variance among years in the intercept (9.720 < *β* < 10.234), so the elevation of the line varies among years, but the curve remains the same. Frequency distribution of log-predicted density of forest birds (g/km^2^) in young forest in France (c).

**References:**

R Core Team (2019). R: A language and environment for statistical computing. R Foundation for Statistical Computing, Vienna, Austria. URL <https://www.R-project.org/>

Shannon, C.E., and Weaver, W. (1949). The mathematical theory of communication. Univ. Ilion’s. Press Urbane, 117p.
