# Supplementary material for: Titmice are a better indicator of bird density in Northern European than in Western European forests
Source: Ecol Evol. 2022 Feb 12;12(2):e8479. doi: 10.1002/ece3.8479 (PMC8840900; doi:10.1002/ece3.8479)
Supplement: Supplementary file 6 — Supplementary Material [file ECE3-12-e8479-s003.docx]

**Appendix 1.**

Kajanus, M.H., Forsman, J.T., Vollstädt, M.G.R., Devictor, V., Elo, M., Lehikoinen, A., Mönkkönen, M., Thorson, J.T., and Kivelä, S.M. 2021. Titmice are a better indicator of bird density in Northern European than in Western European forests. *Ecology and Evolution*.

**Table A1.** Parameter estimates and their 95% confidence intervals for the model including a quadratic relationship between titmouse abundance (measured as biomass) and forest bird density in Finland 2001–2013; parameter estimates and their 95% confidence limits (LOWER/UPPER 95% CI) for the effects of titmouse abundance ($\gamma_{1}$; see Table 1), quadratic term of titmouse abundance ${(\gamma}_{2})$, environmental PC $(\gamma_{3})$, standard deviation of spatial variation (*σ_ω_*) and spatio-temporal variation (*σ_ε_*) on forest bird density.

| PARAMETER | ESTIMATE | LOWER 95% CI | UPPER 95% CI |
| --- | --- | --- | --- |
| Titmouse abundance (γ_1_) | 0.025 | -0.002 | 0.053 |
| [Titmouse abundance]^2^ (γ_2_) | 0.000 | -0.013 | 0.012 |
| Environmental PC (γ_3_) | 0.005 | -0.073 | 0.083 |
| Standard deviation of spatial variation (*σ_ω_*) | 1.810 | 1.489 | 2.131 |
| Standard deviation of spatio-temporal variation (*σ_ε_*) | 0.447 | 0.336 | 0.557 |

*Notes*: Parameter estimates are in log-scale.

**Table A2.** Parameter estimates and their 95% confidence intervals for the model including only a linear relationship between titmouse abundance (measured as biomass) and forest bird density in France 2001–2013; parameter estimates and their 95% confidence limits (LOWER/UPPER 95% CI) of the effects of titmouse abundance ($\gamma_{1}$; see Table 1), environmental PC $(\gamma_{3})$, standard deviation of spatial variation (*σ_ω_*) and spatio-temporal variation (*σ_ε_*) on forest bird density.

| PARAMETER | ESTIMATE | LOWER 95% CI | UPPER 95% CI |
| --- | --- | --- | --- |
| Titmouse abundance (γ_1_) | **0.174** | **0.153** | **0.195** |
| Environmental PC (γ_3_) | -0.020 | -0.048 | 0.007 |
| Standard deviation of spatial variation (*σ_ω_*) | 0.647 | 0.585 | 0.709 |
| Standard deviation of spatio-temporal variation (*σ_ε_*) | 0.295 | 0.270 | 0.321 |

*Notes*: Parameter estimates are in log-scale and parameters that are different from zero at 95% confidence level are highlighted in **bold**. Variance components are not highlighted because they are inevitably non-negative.

**
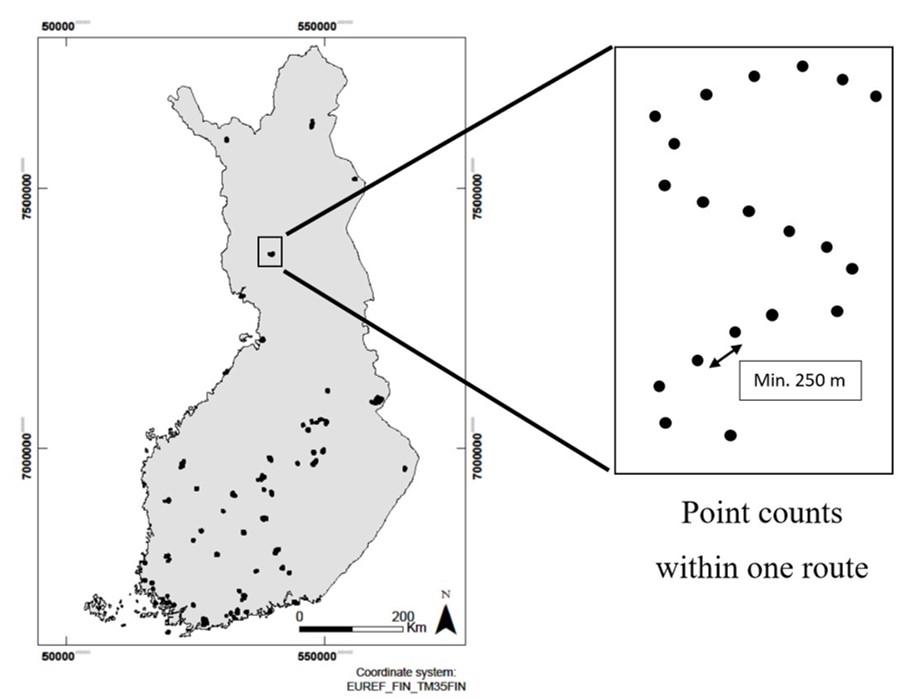
**

**Figure A1**. Map of Finland displaying the sampling points (i.e. point counts) as solid black circles (*n* = 939; points of the same route are nearly overlaid) for all forest bird observations (*N* = 63,156 breeding pairs). The magnified window represents an example layout of the 20 point counts on a sampled route with a minimum distance of 250 m from each other.

**
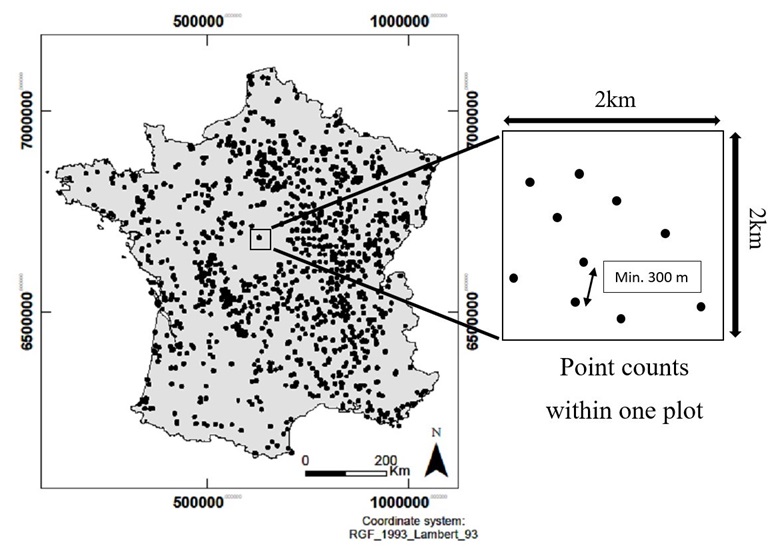
**

**Figure A2.** Map of France displaying the sampling points (i.e. point counts) as solid black circles (*n* = 4342; points of the same plot are nearly overlaid) for all forest bird observations (*N* = 349,886 individuals). The magnified window represents an example layout of the 10 point counts on a sampled plot, with a minimum distance of 300 m from each other.

**
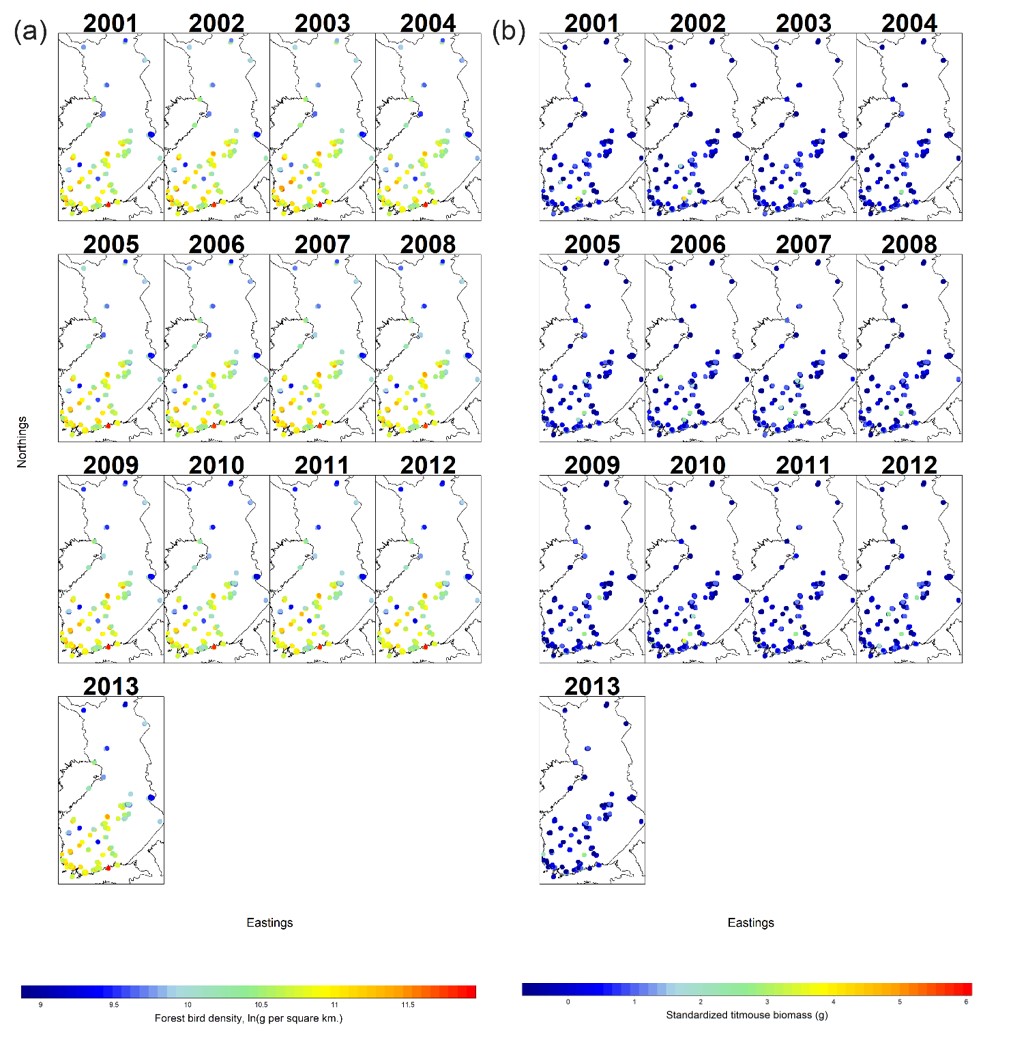
Figure A3.** Forest bird log-predicted density (g/km^2^) (a); and standardized titmouse abundance (measured as biomass; g) (b) for each year (2001-2013) in Finland. Both variables, forest bird predicted density and titmouse abundance are shown by filled circles at the sampling points, where blue indicates low bird density/abundance and red indicates high bird density/abundance.


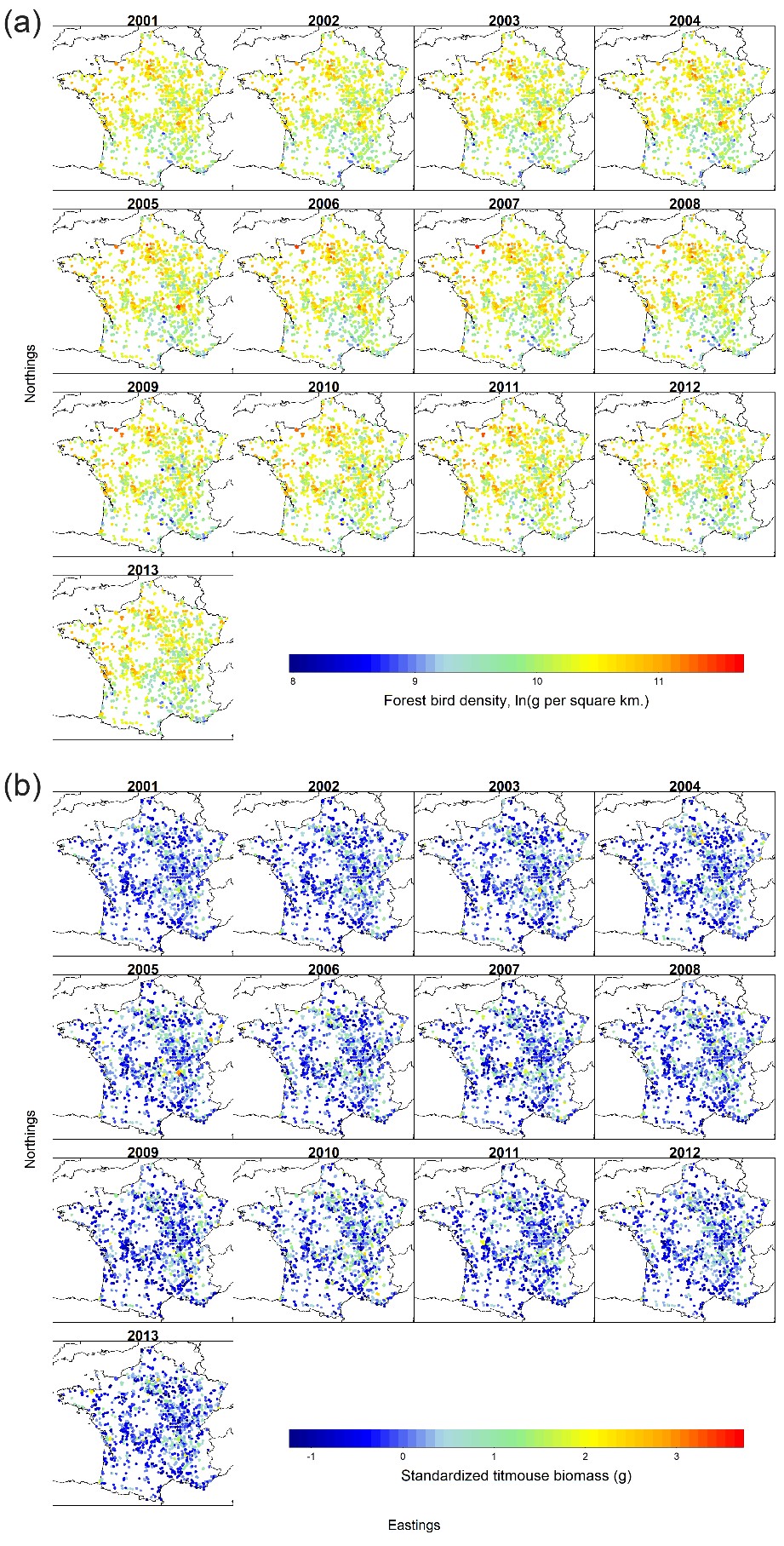


F**igure A4.** Forest bird log-predicted density (g/km^2^) (a); and standardized titmouse abundance (measured as biomass; g) (b) for each year (2001-2013) in France. Both variables, forest bird predicted density and titmouse abundance are shown by filled circles at the sampling points, where blue indicates low bird density/abundance and red indicates high bird density/abundance.
